# A data-driven computational model enables integrative and mechanistic characterization of dynamic macrophage polarization

**DOI:** 10.1016/j.isci.2021.102112

**Published:** 2021-01-29

**Authors:** Chen Zhao, Thalyta X. Medeiros, Richard J. Sové, Brian H. Annex, Aleksander S. Popel

**Affiliations:** 1 Department of Biomedical Engineering, Johns Hopkins University School of Medicine, 720 Rutland Avenue, 613 Traylor Bldg, Baltimore, MD 21205, USA; 2 Department of Medicine, Medical College of Georgia at Augusta University, Augusta, GA 30912, USA

**Keywords:** cell biology, systems biology, in silico biology

## Abstract

Macrophages are highly plastic immune cells that dynamically integrate microenvironmental signals to shape their own functional phenotypes, a process known as polarization. Here we develop a large-scale mechanistic computational model that for the first time enables a systems-level characterization, from quantitative, temporal, dose-dependent, and single-cell perspectives, of macrophage polarization driven by a complex multi-pathway signaling network. The model was extensively calibrated and validated against literature and focused on in-house experimental data. Using the model, we generated dynamic phenotype maps in response to numerous combinations of polarizing signals; we also probed into an *in silico* population of model-based macrophages to examine the impact of polarization continuum at the single-cell level. Additionally, we analyzed the model under an *in vitro* condition of peripheral arterial disease to evaluate strategies that can potentially induce therapeutic macrophage repolarization. Our model is a key step toward the future development of a network-centric, comprehensive “virtual macrophage” simulation platform.

## Highlights

• A large-scale, mechanistic computational model of macrophage polarization
• Model enables quantitative, temporal, dose-dependent, and single-cell simulations
• Unprecedented predictive resolution empowered by extensive model calibration
• Model analyses provide new directions for therapeutic macrophage repolarization

## Graphical Abstract

**Figure:**
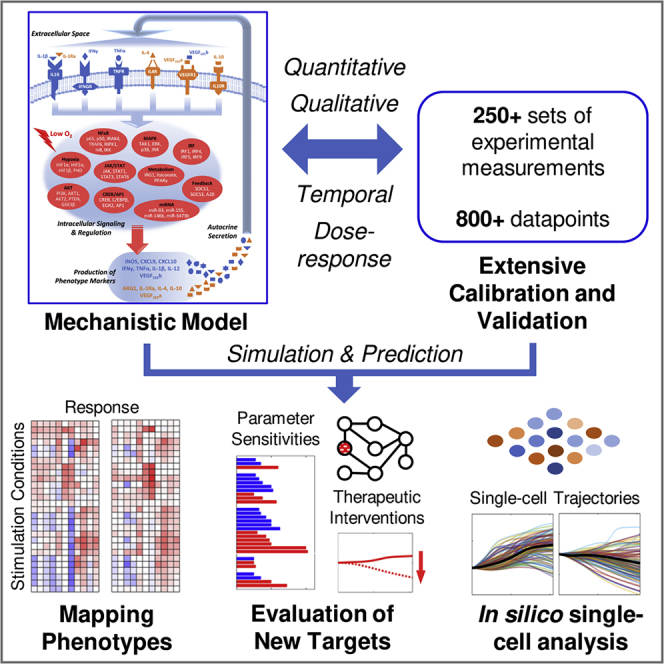

## Introduction

Macrophages are considered a critical component of immune homeostasis and a multifaceted mediator of innate and adaptive immunity. Extensive evidence has shown that these myeloid lineage cells can be dynamically educated by diverse signals in the tissue microenvironment to broadly regulate a variety of cell- and tissue-level processes such as antigen presentation, foam cell formation, inflammation, angiogenesis, tissue remodeling, allergy, infection, and tumorigenesis, in addition to their well-established role as a class of professional phagocytes (Sica and Mantovani, 2012; Mosser and Edwards, 2008). In 2000, the conceptual framework of M1-M2 macrophage polarization was first introduced by Mills et al. to describe the differential phenotypic activation of iNOS (inducible nitric oxide synthase) and arginase pathways observed in strain-selected macrophages in response to certain stimuli *in vitro*, mirroring the classical T helper type-1/type-2 dichotomy (Mills et al., 2000). Over the years, accumulating evidence especially a number of single-cell profiling studies have suggested that the M1 (or canonically activated) and M2 (or alternatively activated) macrophages rather represent two extremes in the entire spectrum of macrophage polarization and activation, and that *in vivo* macrophage phenotypes in health and disease tend to be more continuous and less exclusive: the M1 (and M1-like) macrophages are generally associated with microbicidal, pro-inflammatory, and tumoricidal activities, whereas the M2 (and M2-like) macrophages are shown to possess reparative, immune-suppressive, and angiogenic functions (Xue et al., 2014; Sica and Mantovani, 2012; Mosser and Edwards, 2008; Li et al., 2019; Muñoz-Rojas et al., 2020). Therefore, given the high plasticity and context-dependent functions of macrophages as well as their significant presence and infiltration in pathological tissues observed clinically in many major human diseases, therapeutic strategies that are designed to alter the pathology-driven macrophage polarization have been widely explored and tested in cancer (Cassetta and Pollard, 2018), cardiovascular (Barrett, 2020; Peet et al., 2020; Lindsey et al., 2016) and metabolic diseases (Kazankov et al., 2019), central nervous system disorders (Mammana et al., 2018), and autoimmune diseases (Ma et al., 2019).

From a systems-level standpoint, the M1-M2 macrophage polarization spectrum can be interpreted as the dynamic outcome of a complex network composed of a multitude of driving signaling pathways and intracellular regulatory mechanisms. For example, M1 (and M1-like) polarization can be induced, through respective receptors, by bacterial infection and bacterial products (e.g., lipopolysaccharides); a number of endogenous pro-inflammatory cytokines and growth factors such as IFN-γ (interferon gamma), TNFα (tumor necrosis factor alpha), IL-1β (interleukin-1 beta), GM-CSF (granulocyte-macrophage colony-stimulating factor); and also endogenous danger and stress signals such as HMGB1 (high-mobility group box 1) and HSPs (heat shock proteins) (Atri et al., 2018; Martinez and Gordon, 2014). Likewise, another diverse set of biomolecules including but not limited to IL-4/10/13, transforming growth factor β, VEGF (vascular endothelial growth factor), and prostaglandin E2 are shown to drive M2 (and M2-like) polarization (Wang et al., 2014; Wheeler et al., 2018; Luan et al., 2015). Downstream of these M1-M2 driving factors are highly interactive signal transduction cascades connecting to numerous modules of transcription (e.g., by nuclear factor kappa B [NF-κB], interferon regulatory factors [IRFs]) and post-transcriptional regulation (e.g., by microRNAs), and these modules are known to work in time-dependent, cooperative, or antagonistic fashions to dynamically control the expression of an array of M1 and M2 phenotype readouts (Tugal et al., 2013; Lawrence and Natoli, 2011; Li et al., 2018). Common M1 readouts include high production and secretion of various pro-inflammatory cytokines (e.g., TNFα, IL-1β, IL-12) and chemokines (e.g., C-X-C motif chemokine ligands [CXCLs]), high levels of synthesized reactive oxygen and nitrogen species, increased phagocytosis, and antigen presentation. M2 macrophages are often characterized by significant production of anti-inflammatory cytokines (e.g., IL-10), low secretion of pro-inflammatory cytokines, increased expression of arginase (ARG) proteins, and induction of various cell-surface receptors and markers (e.g., mannose receptor) (Wang et al., 2014; Martinez and Gordon, 2014). In agreement with the systems-level, network-centric view of macrophage polarization, increasing evidence has suggested that under many circumstances the directional changes of isolated M1-M2 readouts may not be definitive or mutually exclusive in terms of understanding macrophage functional phenotypes. For example, a number of canonical M1 stimuli can also induce delayed but significant production of certain M2 markers (e.g., IL-10) *in vitro* (Chang et al., 2007; Hu et al., 2006). In addition, it has been observed that individual tumor-associated macrophages in many cancer types can express both M1 and M2 markers at the same time, possibly owing to the diverse signals in the tumor microenvironment *in vivo* (Reinartz et al., 2014; Helm et al., 2014; Pettersen et al., 2011; Gionfriddo et al., 2020); unique macrophage subpopulations with unconventional marker expression profiles (e.g., distinguishable from M1 or M2) have also been discovered in many disease areas (Tatano et al., 2014; Colin et al., 2014; Chavez-Galan et al., 2015). Therefore, to better integrate the decades of experimental knowledge obtained from traditional “one stimulus/one pathway/one marker” approaches, the systems-level modeling approach that mechanistically incorporates aspects of multi-pathway signal transduction and cross talk, multi-modal modulation of a panel of M1-M2 markers, as well as temporal autocrine/paracrine and feedback regulation is emerging as a new frontier in the investigation of the macrophage polarization spectrum in health and disease.

Following this idea, several systems-level computational models with distinctive features have been formulated to investigate this complex polarization process at the cell level. Palma et al. and Ramirez et al. both used a semi-mechanistic approach based on Boolean gene regulatory networks to identify macrophage activation patterns (Ramirez et al., 2019; Palma et al., 2018). Two more models by Rex et al. and Liu et al. both employed logic-based modeling and ordinary differential equations complemented by calibration against selected experimental datasets of M1-M2 marker profiles to simulate macrophage marker expression signatures in response to exogenous stimuli (Rex et al., 2016; Liu et al., 2019). In comparison, here we have developed and analyzed a multi-pathway computational model formulated based on mass-action and Hill-type kinetics to more accurately characterize the biology and mechanistic regulation in macrophage signaling and polarization. To that end, we have incorporated over 200 sets of quantitative experimental measurements, including over 800 data points obtained from the literature and in-house experiments, into the model calibration and validation steps (achieving a much higher level of predictive capacity and comprehensiveness than any prior macrophage modeling studies have done). To the best of our knowledge, our model is the first large-scale mathematical model that can predictively simulate, from temporal, dose-dependent, quantitative, and single-cell perspectives, both the expression of a panel of macrophage phenotype markers and the dynamic activities of essential transcription factors, intermediate regulators, and signaling hubs, driven by a complex network consisting of seven high-importance macrophage pathways spanning the M1-M2 spectrum. Using this model, we generated comprehensive phenotype maps detailing how the M1-M2 paradigm would respond to single and combinatorial polarizing signals; we also created a population of “*in silico* macrophages” through re-parameterization and probed into their dynamic responses to mechanistically examine the diversity and continuity of macrophage polarization at the single-cell level. Furthermore, we analyzed the model under a specific *in vitro* condition that is used to mimic PAD (peripheral arterial disease) to screen for potential strategies that can induce therapeutic macrophage repolarization (Ganta et al., 2017). Our mechanistic model presented here serves as an important proof-of-concept advancement toward a more comprehensive, kinetics modeling-based realization of an executable network-centric “virtual macrophage” (Wentker et al., 2017).

## Results

### Overview of model scope and formulation

**Figure 1:**
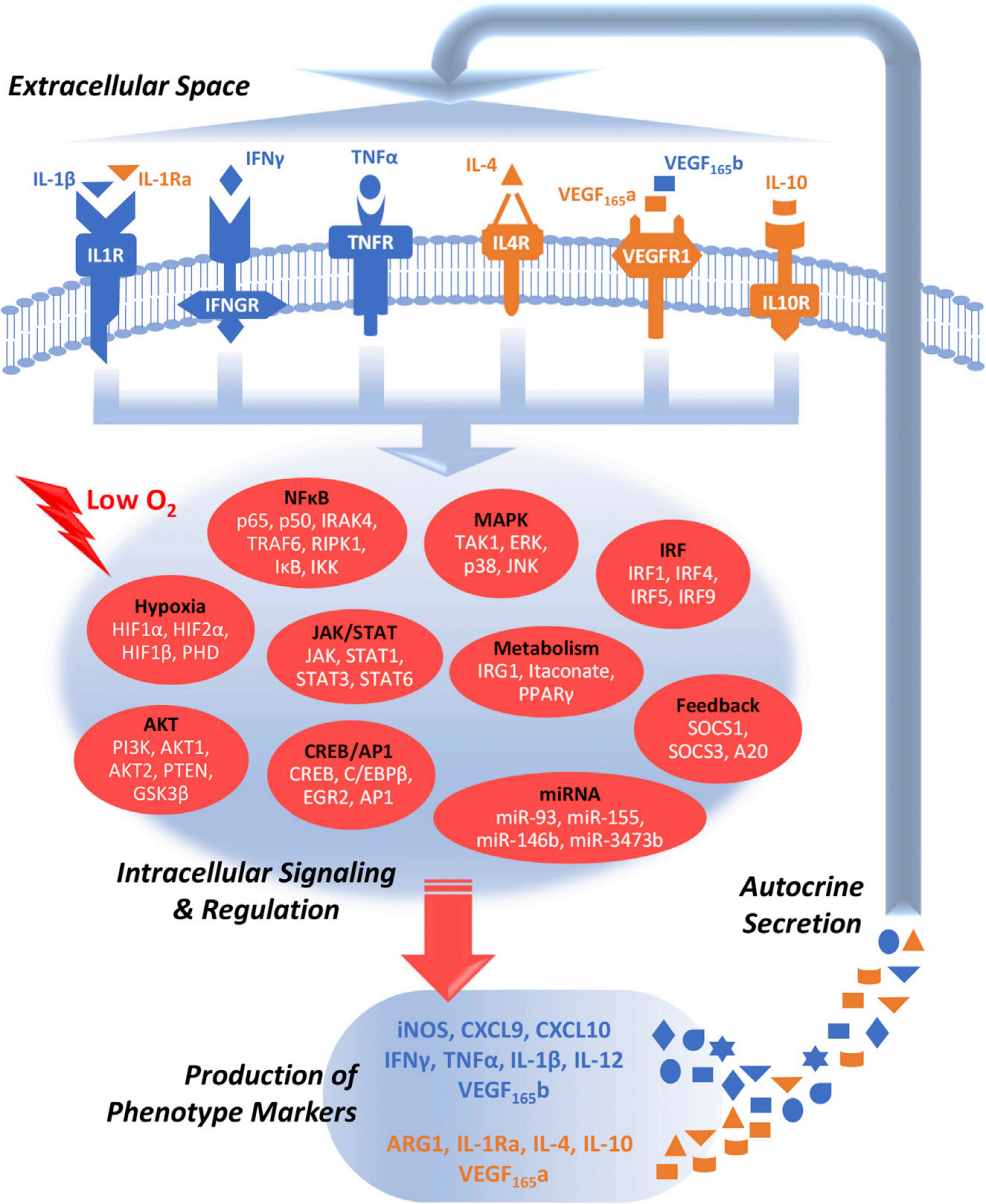
An overview diagram of the mechanistic computational model The model system we developed is composed of seven essential pathways (six receptor-mediated pathways plus an oxygen-sensing pathway) with profound influences on macrophage activation and M1-M2 polarization in health and disease. When this system is perturbed by external driving signals (e.g., cytokine stimuli, hypoxia), the polarizing effects are integratively computed through a complex wired network of intracellular signaling and regulation (highlighted as different modules in red) that then leads to the production and secretion of various M1-M2 markers (blue, M1-associated; orange, M2-associated). Some of these markers themselves are cytokines that drive macrophage polarization and thus can initiate multiple rounds of signaling on macrophages in an autocrine/paracrine manner to produce a highly diverse spectrum of phenotypes. A more detailed model diagram is shown in Figure S16 in Supplemental Information.

A total of seven driving pathways were included in this model (Figure 1) based on a manual curation of the macrophage literature: five pathways are relatively well-established M1 (IFNγ, TNFα, IL-1β) and M2 (IL-4, IL-10) stimuli, and the remaining two pathways (hypoxia, VEGF) were selected given their significance in PAD, which is our primary disease area of interest, as both factors are known to be critical drivers and regulators of PAD pathophysiology (Martinez and Gordon, 2014; Ouma et al., 2012). The general mechanistic framework of this model, which uses mass-action and Hill-type kinetics to capture biochemical details in pathway signal transduction, follows the same literature-based, data-driven model formulation logic as described in a previous modeling study from our group, and from there the IL-4/IFNγ/hypoxia pathways were further enriched with new signaling mechanisms and then implemented in a similar manner as reported before (Zhao et al., 2019). For the IL-10 pathway, a previous model by Braun et al. (2013) was taken as a basis from which we further added corresponding mechanisms to describe the activation of PI3K/AKT (phosphoinositide 3-kinase/protein kinase B) and ERK (extracellular signal-regulated kinase) by IL-10 and also the inhibitory effect of SOCS1 (suppressor of cytokine signaling 1) on IL-10/STAT3 (signal transducer and activator of transcription 3) signaling (Xiao et al., 2019; Zhu et al., 2015; Niemand et al., 2003). The VEGF pathway in the model focuses on the interactions between two functionally distinct VEGF isoforms (165a and 165b) and VEGFR1, which is the major VEGF receptor expressed on macrophages and that VEGFR1 can signal through various modules including PI3K/AKT, STAT3, and ERK to regulate downstream targets (Weddell et al., 2018; Ganta et al., 2019; Boscolo et al., 2011). For TNFα and IL-1β pathways, both stimuli, through different intermediates, can induce the MAPK (mitogen-activated protein kinase) cascades including phosphorylation of p38, ERK, and JNK (c-Jun N-terminal kinase); turn on transcription factors such as CREB (cAMP response element-binding protein), C/EBPβ (CCAAT/enhancer-binding protein beta), and AP-1 (activator protein 1); and drive the canonical activation of NF-κB (Hu et al., 2005; Ermolaeva et al., 2008); here the mechanistic pathway structures were adapted from the toll-like receptor map and also structures proposed by several published models of NF-κB signaling (Werner et al., 2008; Caldwell et al., 2014; Salim et al., 2016; Oda and Kitano, 2006). In addition to the pathway modules, a number of inter-pathway cross talk and feedback mechanisms (e.g., through SOCSs, microRNAs [miRNAs]) were modeled explicitly, and further downstream are the signaling network-mediated differential regulation of a diverse M1-M2 marker panel, while autocrine/paracrine regulation was incorporated for the 6 ligand/receptor pathways (covering 8 secreted M1-M2 cytokine markers). More mechanistic details of model formulation are described in Transparent Methods and Tables S1 and S2 (full description of all model mechanisms, reactions, species, initial conditions, and parameters).

### Model calibration and validation against experimental data

**Figure 2:**
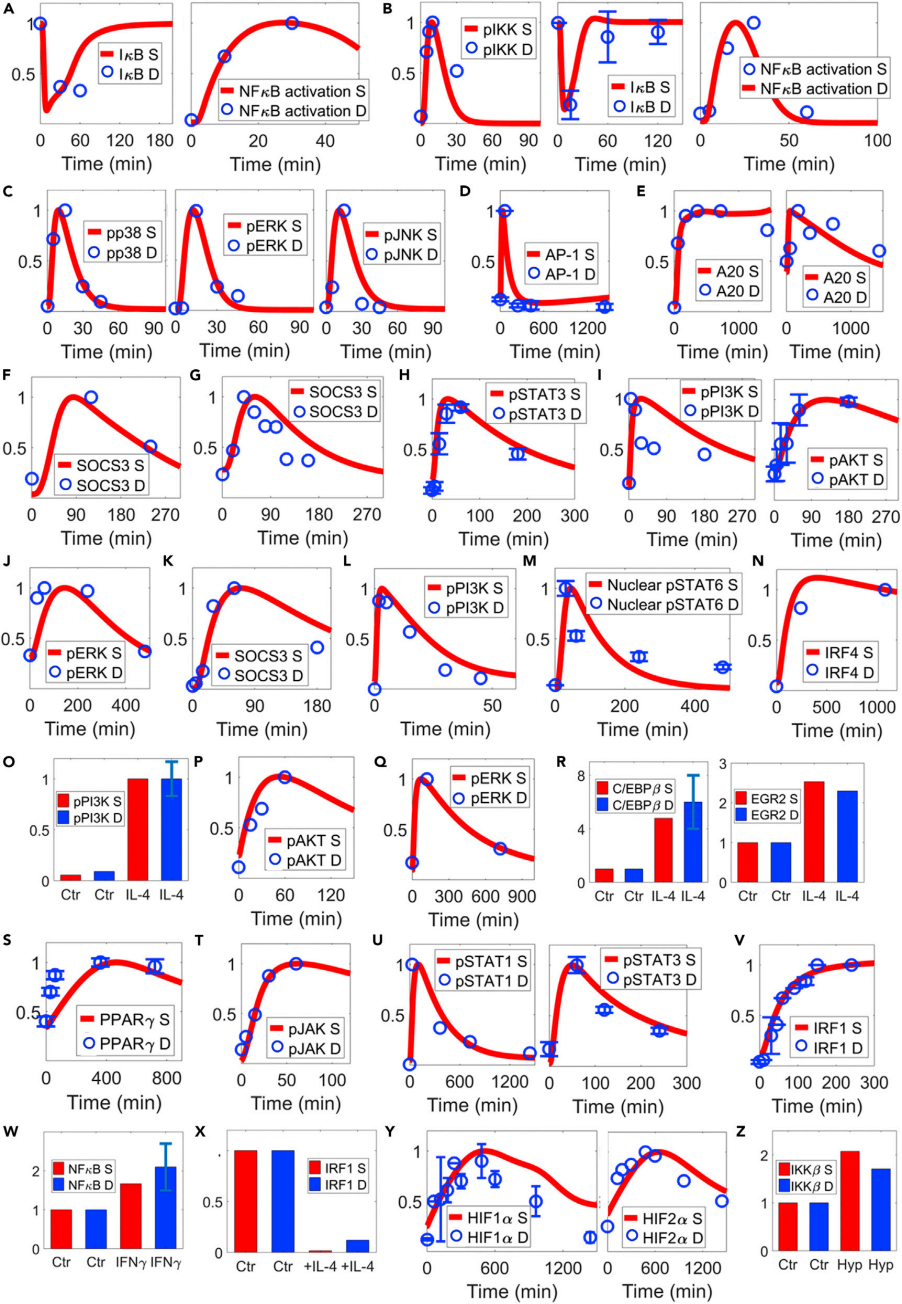
Model calibration of macrophage pathway signal transduction (part 1); parts 2–4 are shown in Figures S2–S4 Model simulations and corresponding experimental data obtained from macrophages are shown together (references are listed in the order of the data mentioned). (A) IκB expression (Suzuki et al., 2002) and NF-κB activation (Hu et al., 2005) in response to IL-1β. (B–D) (B) TNFα can induce activation of IKK/IκB/NF-κB axis (Zhang et al., 2016; Etemadi et al., 2015; Lo et al., 2011), (C) MAPKs (Ermolaeva et al., 2008), and (D) AP-1 (Yarilina et al., 2011). (E–G) (E) Both IL-1β (left) and TNFα (right) can induce A20 (Hu et al., 2014); (F and G) both IL-1β and TNFα can induce SOCS3 (Wong et al., 2006; Ehlting et al., 2007). (H–J) IL-10 activates STAT3, PI3K/AKT (Zhu et al., 2015), and ERK (Xiao et al., 2019). (K) IL-10 also upregulates SOCS3 (Zhu et al., 2015). (L) VEGF induces PI3K activation (Weddell et al., 2018). (M–S) (M) IL-4 induces STAT6 activation (Sheldon et al., 2013), (N) IRF4 expression (El Chartouni et al., 2010), (O and P) PI3K/AKT (O'connor et al., 2007; Covarrubias et al., 2016), and (Q) ERK activation (Wang et al., 2010); (R) IL-4 also upregulates C/EBPβ, EGR2 (Veremeyko et al., 2018), and (S) PPARγ (Yao et al., 2016) expression. (T–W) (T) IFNγ activates JAK (Blanchette et al., 2003) and downstream (U) STAT1 (Majoros et al., 2016) and STAT3 (original data, see Figure S9B) and induces (V) IRF1 expression (Ramsauer et al., 2007) and (W) NF-κB activation (Vila-Del Sol et al., 2007). (X) IFNγ-induced IRF1 expression can be repressed by IL-4 pretreatment (Coccia et al., 2000). (Y) Hypoxia stabilizes cellular HIF1α and HIF2α (Ortiz-Masia et al., 2014; Frede et al., 2006) and (Z) enhances IKKβ expression (Fang et al., 2009). (A–Z) All values are for protein levels and normalized (y axes are relative expression levels). For normalization of results (simulation and data in A–Z): IκB in (A and B, R, W, and Z) normalized to the respective t = 0 (Ctr) values; (N, T, V), NF-κB in (A), and AKT in (I and P) normalized to their respective values at the last experimental time points; (O) normalized to the values at 15 min of IL-4 treatment; (X) normalized to the Ctr (IFNγ-treated) condition; all others normalized to their respective maximum values. S, simulation; D, experimental data; Hyp, hypoxia; Ctr, control/untreated condition (except in X as described above).

**Figure 3:**
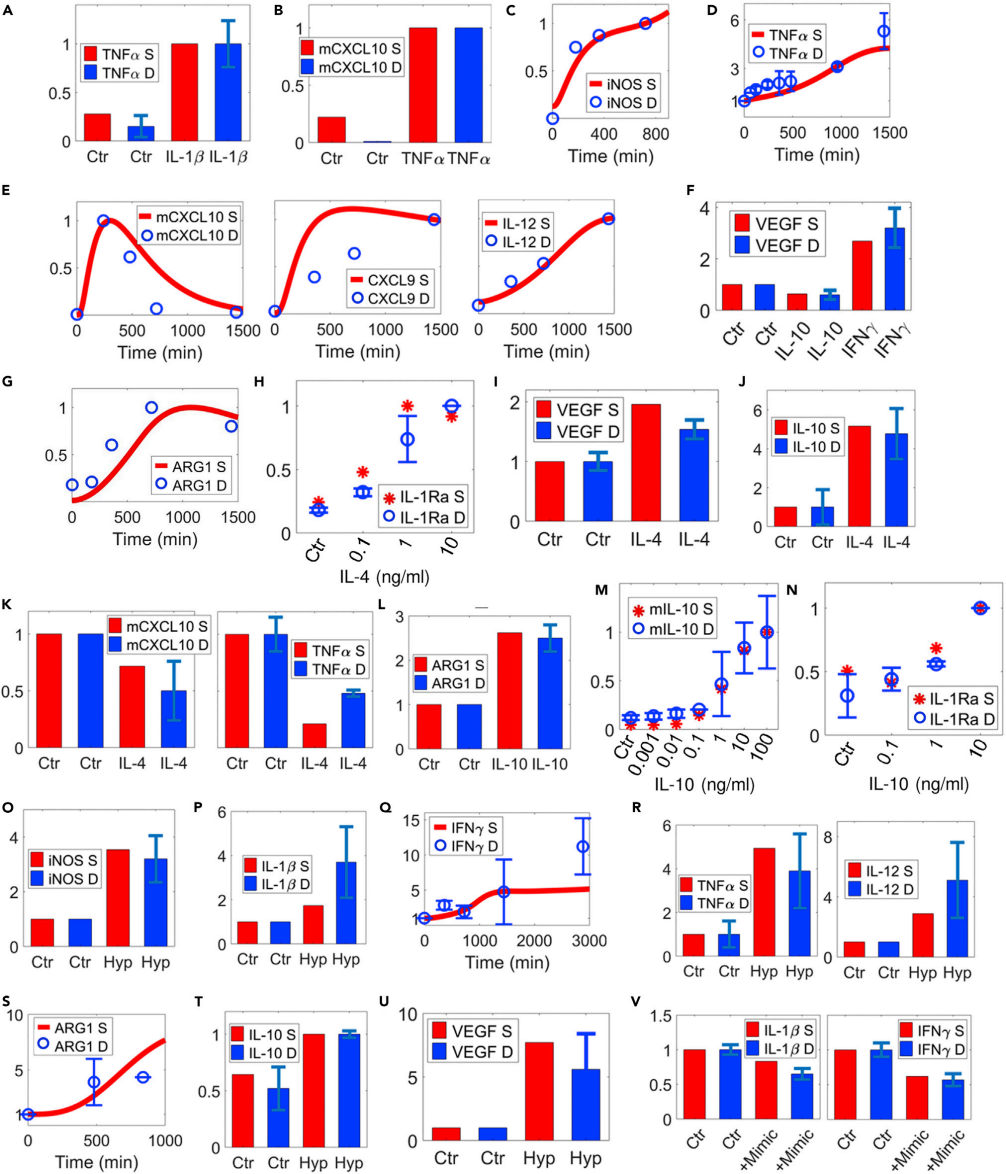
Model calibration of macrophage phenotype marker regulation (part 1); part 2 is shown in Figure S5. Model simulations and corresponding macrophage experimental data are shown together (references are listed in the order of the data mentioned). (A) IL-1β induces TNFα secretion (Sato et al., 2012). (B) TNFα induces CXCL10 (mRNA) expression (Rauch et al., 2015). (C–E) (C) IFNγ induces iNOS expression (Lin et al., 2008), (D) TNFα secretion (Vila-Del Sol et al., 2008; Vila-Del Sol et al., 2007), (E) CXCL10 (mRNA) (Rauch et al., 2015), and secretion of CXCL9 and IL-12 (Su et al., 2015). (F) IFNγ also promotes VEGF secretion, whereas IL-10 represses it (Wu et al., 2010). (G–J) (G) IL-4 induces the expression of ARG1 (Schleicher et al., 2016) and production of (H) IL-1Ra (dose response) (Joyce et al., 1996) and (I and J) VEGF and IL-10 (Lee et al., 2017). (K) IL-4 also inhibits CXCL10 (mRNA) (Piccolo et al., 2017) and TNFα production (Lee et al., 2017). (L–R) (L–N) IL-10 induces ARG1 expression (Nakamura et al., 2015) and production of itself (mRNA, dose response) (Staples et al., 2007) and IL-1Ra (dose response) (Joyce et al., 1996). Hypoxia upregulates the production and secretion of multiple M1 markers including (O) iNOS (Gao et al., 2017), (P) IL-1β (Fang et al., 2009), (Q) IFNγ (Carta et al., 2001; Acosta-Iborra et al., 2009), and (R) TNFα (Hempel et al., 1996) and IL-12 (Acosta-Iborra et al., 2009). (S–U) Hypoxia also promotes M2 markers including (S) ARG1 (Takeda et al., 2010; Gao et al., 2017), (T) IL-10 (Dace et al., 2008), and (U) VEGF (Ramanathan et al., 2003). (V) Overexpression of miR-93 (through miR mimics) decreases hypoxia-induced IL-1β and IFNγ secretion (Ganta et al., 2017). (A–V) All values are normalized (y axes are relative expression levels) and are for proteins unless noted otherwise. For normalization of results (simulation and data in A–V): (D, F, I–L, O–S, U) normalized to their respective t = 0 (Ctr) values; (V) normalized to the Ctr (hypoxia) condition; (A, B, and T) normalized to their respective values at 24 h of IL-1β/TNFα/hypoxia treatments; (C) CXCL9 and IL-12 in (E) normalized to their respective values at the last experimental time points; all others normalized to their respective maximum values. S, simulation; D, experimental data; Hyp, hypoxia; Ctr, control/untreated condition (except in V as described above).

**Figure 4:**
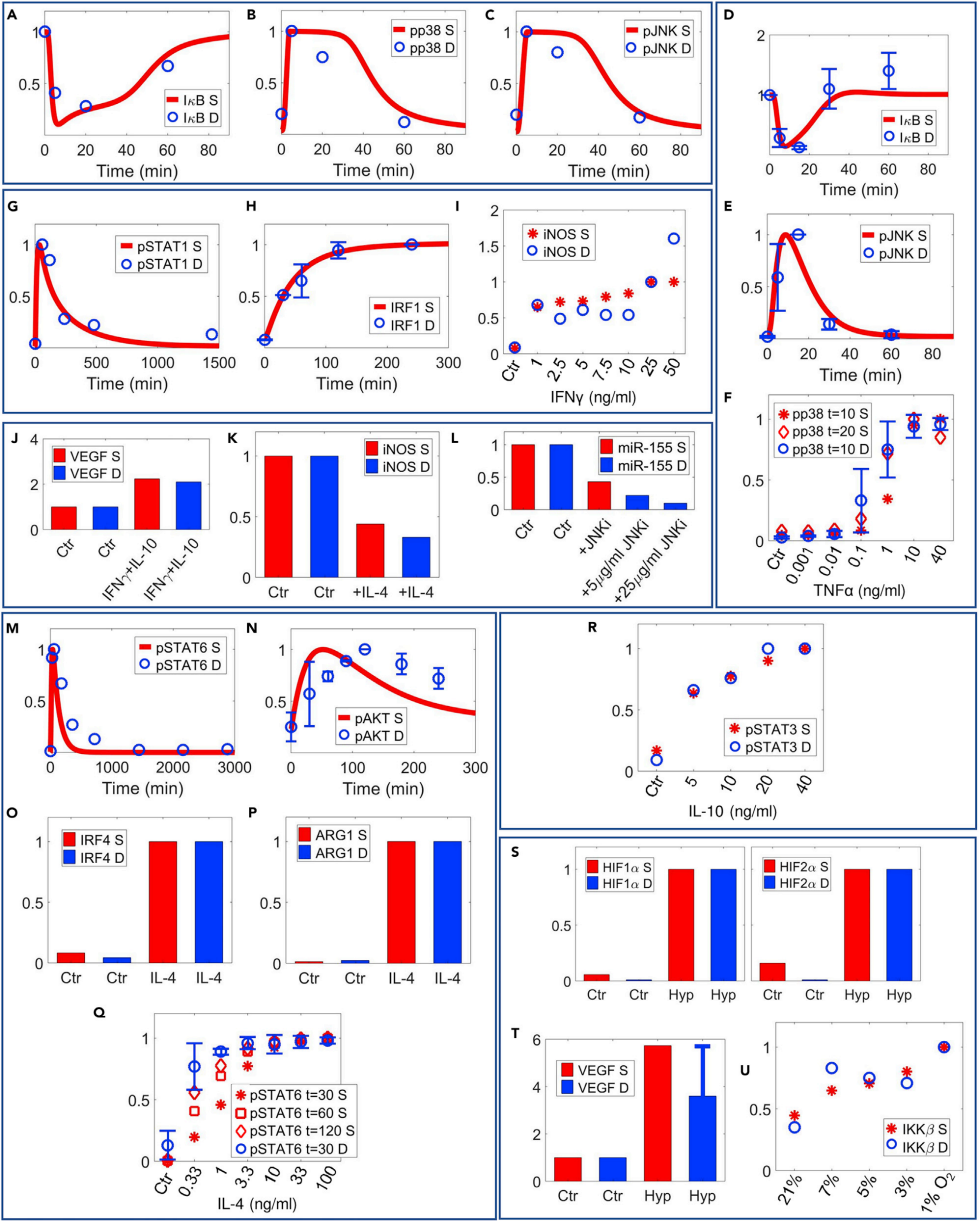
Model validation of essential pathway and marker signatures against untrained experimental data Model simulations and corresponding macrophage experimental data (not included in calibration) are shown together (references are listed in the order of the data mentioned). (A–C) IL-1β induces transient IκB degradation and activation of p38 and JNK (Hu et al., 2005). (D–F) TNFα also induces IκB degradation and JNK activation (Pobezinskaya et al., 2008), and dose-dependent activation of p38 (Winston et al., 1997). (G–I) IFNγ activates STAT1/IRF1 axis (Piccolo et al., 2017) and dose-dependently induces iNOS expression (Vila-Del Sol et al., 2007). (J) Simultaneous treatment of IFNγ and IL-10 moderately upregulates VEGF secretion (Wu et al., 2010). (K) IL-4 pretreatment decreases IFNγ-induced iNOS (Coccia et al., 2000). (L) Inhibition of JNK decreases TNFα-induced upregulation of miR-155 (O'connell et al., 2007). (M–P) IL-4 induces time course activation of STAT6 (Zhu et al., 2014) and AKT (Mccormick et al., 2016) and downstream expression of IRF4 and ARG1 (Binder et al., 2013). (Q and R) (Q) Dose response of IL-4-mediated STAT6 activation (Heller et al., 2008); (R) dose response of IL-10-mediated STAT3 activation (Naiyer et al., 2013). (S–U) Hypoxia stabilizes HIF1α/HIF2α and induces VEGF secretion (Fang et al., 2009); hypoxia also dose-dependently induces IKKβ expression (Cummins et al., 2006). (A–U) All values are normalized (y axes are relative expression levels) and are for proteins unless noted otherwise. For normalization of results (simulation and data in A–U): (A, D, J, and T) normalized to their respective t = 0 (Ctr) values; (K) normalized to the Ctr (IFNγ-treated) condition; (L) normalized to the Ctr (TNFα-treated) condition; (O and P) normalized to their respective values at 24 h of IL-4 treatment; (S) normalized to the respective values at 18 h of hypoxia; (H) normalized to the values at the last experimental time point; (I) normalized to the values at 25 ng/mL of IFNγ; all others normalized to their respective maximum values. S, simulation; D, experimental data; Hyp, hypoxia; Ctr, control/untreated condition (except in K and L as described above).

Details of calibration (Figures 2 and 3, and S2–S5) and validation (Figure 4) results are presented and discussed in the following sections and in Supplemental Information. Overall, this model contains 67 “unique” species (e.g., functionally unique proteins, mRNAs, miRNAs, and non-gene compounds) and their quantitative initial conditions (in terms of absolute copy numbers), which together represent the resting states of unpolarized macrophages were calibrated against respective quantitative measurements the in literature (see Transparent Methods and Table S2 for more details regarding the initial condition calibration procedure). From there, different stimulation conditions were converted to quantitative inputs into the model to generate a large number of simulations, which were simultaneously calibrated, using a global optimization approach, against extensive experimental data from both literature and in-house experiments (including over 200 sets of time course, single time point, and dose-response measurements). Our final model was then validated against an independent set of data (not included in calibration) that reflects the dynamic regulation of key signaling axes in the model. A comparison between our model and previous studies that modeled macrophage polarization is shown in Figure S1.

### Calibration of multi-pathway signal transduction

The signal transduction of IL-1β initiates from receptor-ligand binding and travels downstream through the IRAK/TRAF6 (interleukin 1 receptor associated kinase/TNFR-associated factor 6) axis (Figures S2B–S2D) to activate IKK (IκB kinase, Figure S2E), which results in IκB (inhibitor of κB) degradation and subsequent nuclear translocation of NF-κB complex (Figures 2A and S2F). IL-1β also activates the PI3K/AKT axis (Figure S2G) and MAPKs (Figures S2H–S2J), in addition to several other aforementioned transcription factors. Compared with IL-1β, TNFα signaling, through an axis controlled by RIP1 (receptor interacting protein 1, Figure S2O), activates a similar group of effector molecules (cascades of NF-κB [Figures 2B and S2Q and S2T], MAPKs [Figures 2C and S2U–S2W], AP-1 [Figure 2D], CREB [Figures S3A and S3B], C/EBPβ [Figure S3C]). Both IL-1β and TNFα can induce the expression of various negative feedback regulators, such as A20 (Figures 2E and S3D, also known as TNFAIP3 [TNFα-induced protein 3]), SOCS3 (Figures 2F and 2G), and SOCS1 (Figures S2K and S3E). Furthermore, they also upregulate miR-155 (Figures S3F–S3H), a canonical M1-inducible miRNA, and downregulate miR-93 (Figures S2L and S3K), which targets IRAK4 (Figure S3L), IRF9 (Figure S4Y), and VEGF (Figure S5Y) in the model. For the M2 side, IL-10 is known to induce phosphorylation and activation of STAT3 (Figures 2H and S3M–S3O), PI3K/AKT (Figure 2I), and ERK (Figure 2J). SOCS3, a negative regulator of JAK (Janus kinase)/STAT signaling, is also inducible by IL-10 (Figure 2K). As an M2 stimulus, IL-10 represses miR-155 (Figure S3P) and induces miR-146b (Figure S3Q), which can target STAT1 (Figure S3R) to limit pro-inflammatory signaling. IL-10 also directly interacts with the IL-4 pathway by upregulating the production of IL-4 receptors (Figure S3S). Another novel M2 axis included in the model is the VEGF/R1 pathway; upon binding with the pro-angiogenic VEGF isoform (e.g., VEGF_165_a), VEGFR1 can initiate receptor phosphorylation (Figure S3U) and downstream signal activation (Figures 2L and S3V).

The pathway structures of IL-4/IFNγ/hypoxia were initially taken from our previous model and then further enriched with additional mechanistic details of pathway feedback and cross talk (Zhao et al., 2019). Ligation of IL-4 receptors will lead to subsequent activation of STAT6 (Figures 2M and S3X), IRF4 (Figures 2N and S4A), PI3K/AKT (Figures 2O–2P, and S4B), and ERK (Figure 2Q) and induce the expression of EGR2 (early growth response 2) and C/EBPβ (Figures 2R and S4F) as well as PPARγ (peroxisome proliferator activated receptor gamma), which is a signature gene in macrophage lipid metabolism (Figures 2S and S4C). For the IFNγ pathway, in addition to the canonical JAK/STAT1/IRF1 axis (Figures 2T–2V, S4J, and S4M and S4N), we further modeled the effect of IFNγ-induced activation of STAT3 (Figures 2U and S4K) and its functional antagonism against STAT1, as well as the direct and indirect effects of IFNγ on IL-1β signaling (Figures 2W and S4L). In terms of cross talk, both IL-4 and IFNγ can selectively induce SOCS proteins (Figures S4D and S4O) and miRNAs (Figures S4E and S4P–S4Q) to influence other pathway modules, and IL-4 represses IFNγ-induced IRF1 expression (Figure 2X). The hypoxia pathway centers on the oxygen-dependent stabilization of HIF1α (hypoxia-inducible factor 1 alpha) and HIF2α (Figures 2Y and S4R–S4U); the impact of hypoxia on IRF1 activation (Figure S4V), IKK expression (Figures 2Z and S4W), and miR-93 and its downstream target regulation (Figures S4X and S4Y) were also included. Besides, the cellular abundance of HIF1/2α can be controlled by signaling activities downstream of IL-4 and IFNγ in a mutually antagonistic manner (Figures S4G and S4I).

Besides the intracellular signal transductions, each cytokine-activated pathway in our model has a corresponding module that is calibrated to mechanistically describe the dynamic events of ligand-receptor binding, receptor activation and internalization, recycling, and degradation (e.g., IL-1β [Figure S2A], TNFα [Figures S2M and S2N], VEGF [Figure S3T], IL-4 [Figure S3W], IFNγ [Figure S4H]).

### Calibration of M1-M2 marker regulation

The three pro-inflammatory drivers in the model, IL-1β, TNFα, and IFNγ, are known to induce macrophage expression and secretion of an array of typical M1 markers: themselves such as IL-1β and TNFα (Figures 3A, 3D, S5A–S5C, and S5I), other pro-inflammatory chemokines and cytokines such as CXCL9-10 and IL-12 (Figures 3B, 3E, and S5J), canonical M1 intracellular marker iNOS (Figures 3C and S5F–S5H), and also M1-associated metabolite such as itaconate (Figure S5K). In parallel with their pro-inflammatory functions, these M1 drivers can also induce the expression of certain anti-inflammatory and pro-angiogenic molecules such as IL-1Ra (IL-1 receptor antagonist, Figure S5D) and VEGF (Figures 3F and S5E), as a potential mechanism to limit excessive inflammation. On the other side, anti-inflammatory drivers such as IL-4 and IL-10 can produce strong M2 responses including upregulation of the canonical M2 intracellular marker ARG1 (Figures 3G and S5L–S5N) and secretion of anti-inflammatory factors such as IL-10 (Figures 3J, 3M, and S5Q) and IL-1Ra (Figures 3H, 3N, S5P, and S5S). In addition, IL-4 and IL-10 can differentially regulate VEGF production (Figures 3F, 3I, and S5O) and downregulate the production and secretion of multiple M1 cytokines (Figures 3K and S5O) and iNOS (Figure S5R). Hypoxia, through HIFs and other transcriptional and post-transcriptional regulators, induces both M1 markers such as iNOS (Figure 3O), IL-1β (Figure 3P), IFNγ (Figure 3Q), TNFα, and IL-12 (Figure 3R) and M2 markers such as ARG1 (Figure 3S) and IL-10 (Figures 3T and S5W), in addition to VEGF (Figures 3U and S5T–S5U), which is a master pro-angiogenic factor downstream of HIFs. An example of hypoxia-mediated post-transcriptional regulation of macrophage polarization is through miR-93, which is downregulated by hypoxia in macrophages (Figure S4X), whereas its overexpression can indirectly suppress the macrophage production of pro-inflammatory cytokines such as IL-1β, IFNγ, and TNFα (Figures 3V and S5X). In addition to these quantitative data, we also incorporated a number of qualitative experimental observations relating to specific signaling/marker regulation within our multi-pathway model scope to further constrain our model behaviors (presented in Figure S6).

### Quantitative model validation

For model validation, a separate quantitative dataset (not included in calibration) was compiled using the criterion as described in Transparent methods. Modular comparisons between experimental results (e.g., macrophages treated with high concentrations of stimuli *in vitro*) and corresponding model simulations suggested that our model can correctly predict, from quantitative and temporal aspects, the activation of essential signal transduction cascades and marker expression for the individual pathways modeled (Figures 4A–4E, 4G, 4H, 4M–4P, and 4S–4T). Moreover, the potential of model applications and analyses beyond isolated *in vitro* conditions was again demonstrated by the validation against numerous experimental dose-response curves (Figures 4F, 4I, 4Q, 4R, and 4U) as well as data of combination treatments (Figures 4J–4L) within our model scope.

### Generation and interpretation of dynamic macrophage polarization maps

**Figure 5:**
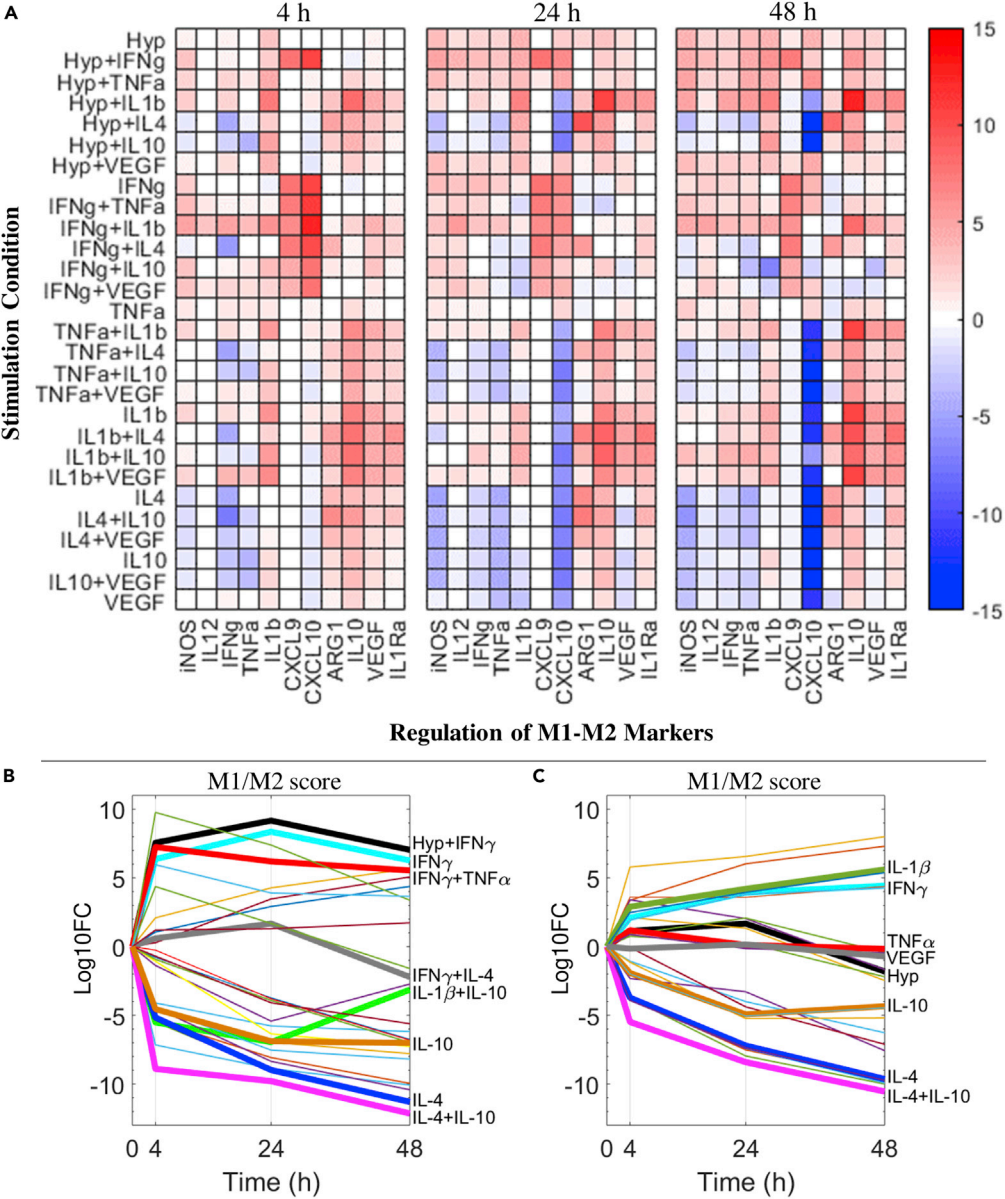
Macrophage polarization map and time course M1-M2 profiles under single and combined stimulation (A) A model-generated map of M1-M2 marker regulation (at 4, 24, and 48 h of stimulation) by macrophages under different stimulation conditions *in vitro* (7 cases of single stimulus, 21 cases of pairwise combined stimuli). Among the M1-M2 markers described, iNOS, ARG1, IL-12, IL-1Ra, and CXCL9 are protein levels; CXCL10 is mRNA level; and the remaining ones are the respective protein production rates calculated by the model. All results are normalized to the untreated/t = 0 values and then log2 transformed. (B) Time course trajectories of relative dominance of M1-M2 features (in terms of M1/M2 scores; positive, M1-like; negative, M2-like) in all the *in vitro* stimulation conditions. (C) Trajectories from the same analyses but under simulated *in vivo* stimulus concentrations. (B and C) All results are normalized to the M1/M2 score calculated at untreated/t = 0 and then log10 transformed. Some of the trajectories are bolded and labeled for better data illustration. Hyp, hypoxia (2% O_2_).

Using our mechanistic model, we have constructed detailed activation maps (for all single and pairwise stimuli described by our model) to comprehensively characterize the dynamic activities of macrophage intracellular signal transduction and the resulting regulation of phenotype markers under typical *in vitro* conditions (e.g., cytokine concentrations in the ng/mL range). Comparisons between the relative fold changes of different markers at early (4 h), delayed (24 h), and late (48 h) time points (Figure 5A) revealed that the strength of the polarization response is highly time dependent at the level of individual markers, although such M1-M2 markers do respond accordingly to the canonical M1-M2 drivers during the simulated time span; at the level of transcription factor activations, the time dependence is, as expected, much stronger as shown in Figure S8A. In addition, our simulations in Figure 5A suggest that the expression of M1-M2 markers are often not mutually exclusive even under single stimuli conditions (e.g., TNFα, hypoxia, IL-1β), and for stimulus combinations the resulting response landscape would very likely differ from the simple qualitative addition of the effects from individual pathways (e.g., IL-1β+IL-10, IFNγ + IL-10). We also calculated the M1/M2 scores (metrics used to estimate the relative dominance of M1 versus M2 response, see Transparent methods for detailed definition) for all single and pairwise stimulus conditions at the three time points evaluated in the activation map. Analyses of time course trajectories (Figure 5B) further indicate that the polarization process can be non-monotonic as seen from examples of self-promoting (a strong overall phenotype response that is augmenting over time, e.g., by IL-4, IL-4+IL-10), self-limiting (a strong response followed by gradual decay or stabilization, e.g., by IFNγ, IFNγ+TNFα), and self-repolarizing (one phenotype response in the beginning and later significantly shifts toward the opposite phenotype, e.g., by IFNγ+IL-4, IL-1β+IL-10). The same analyses were also performed for cytokine stimuli at 100-fold lower concentrations (in the high pg/mL range) to simulate the potential influence of upregulated cytokines *in vivo* on macrophage polarization (Stenken and Poschenrieder, 2015). Our modeling results suggest that for most cases, even modest upregulation of cytokines *in vivo* can still induce differential regulation of transcriptional activities (Figure S8B) in macrophages (although different from their respective *in vitro* patterns) and elicit detectable responses of M1-M2 markers over time (Figure S7), whereas the intensities of the *in vivo* phenotype responses (Figure 5C) are generally much weaker than those under *in vitro* conditions (Figure 5B). These findings again reinforce the argument that macrophage polarization in health and disease should be integratively evaluated as a multi-pathway, multi-marker, and time-dependent response, and that system-level modeling is an effective tool to enable quantitative and mechanistic understanding of the full picture of this response, especially in settings of pathophysiological tissue microenvironments, which are complex and multifactorial by nature.

### Model-based sensitivity analyses suggest potential strategies to induce therapeutic macrophage polarization in PAD

**Figure 6:**
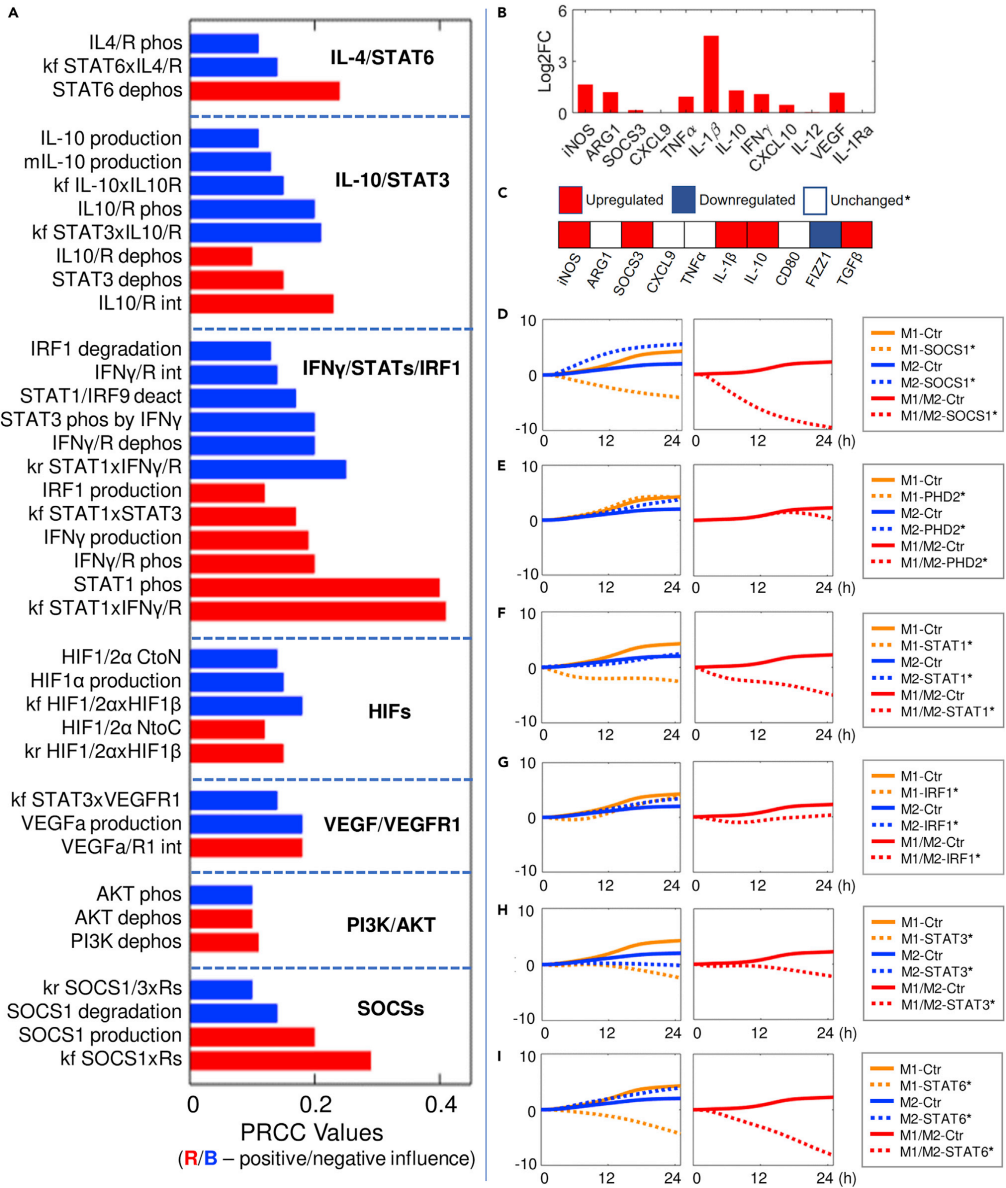
Macrophage response under hypoxia/HSS and evaluation of sensitivity analysis-derived macrophage repolarization strategies (A) Sensitivity indices (red, positive; blue, negative) of the most influential model parameters (e.g., absolute PRCC values great than 0.1) that control M1 and M2 marker expression (in terms of the 24 h time course integral of M1/M2 score) in hypoxia (2% O_2_). The identified parameters are categorized into seven modules, and their functions are briefly described. (B) Simulated profile of macrophage M1-M2 marker expression at 8 h under hypoxia/HSS (represented by model as 2% O_2_). Expression levels are normalized to the t = 0 (control condition) values and then log2 transformed. (C) Summary of experimentally measured directional regulation of multiple macrophage M1-M2 markers under HSS based on our original *in vitro* data (experimental results of individual markers are shown in detail in Figures S9 and S10). The category of unchanged also includes markers that are experimentally undetectable. (D–I) Simulated M1 (orange), M2 (blue) profiles (e.g., M1 and M2 scores) and overall M1/M2 scores (red) over time under hypoxia with various targeted interventions (label as “species∗”) proposed by the sensitivity analysis. Results are normalized to the respective values at t = 0 and then log10 transformed (y axes). Implementation of targeted interventions: (D) SOCS1 inhibition, 10x k101; (E) PHD2 inhibition, 0.1x kf62; (F) STAT1 inhibition, 0.1x kf3; (G) IRF1 inhibition, 10x k85; (H) STAT3, 0.1x k127; (I) STAT6, 0.1x k26. (A) Details of the listed parameters are described in Table S1 (parameter labels from top to bottom: kf102, kf24, k26; k156, k44, kf144, kf145, kf146, kr145, k127, k148; k85, k12, k117, k134, kr2, kr3, k52, kf131, k73, kf2, k4, kf3; kf58, k54, kf60, kr58, kr60; kf207, k72, k201; k138, k140, k137; kr11, k101, k135, kf11).

We then analyzed the model under a simulated experimental condition of PAD, which is a highly prevalent cardiovascular disease characterized by reduced lower limb blood flow and ischemia, for which therapeutic macrophage polarization has been recently proposed as an emerging route to enhance tissue regeneration and perfusion recovery (Ganta et al., 2017, 2019; Gotze et al., 2020). Hypoxia serum starvation (HSS) is the chosen *in vitro* representation of PAD (Ganta et al., 2017, 2019), and we further simplified it and considered only hypoxia as the essential input for our primary model analyses, because the proliferative effect of serum was so variable and thus was not taken into account during the model formulation. Our model simulations (Figures 6B and S11B) as well as our original experimental data summarized from western blot, qPCR, and flow cytometry analyses (Figures 6C, S9, S10, S14, and S15) together revealed a noncanonical phenotype under HSS that showed selective and time-dependent induction of both M1 and M2 markers. Therefore, to identify potential targets that can effectively reprogram macrophages into less pro-inflammatory and more pro-angiogenic states under HSS, we performed global sensitivity analysis using the PRCC (partial rank correlation coefficient) algorithm to search for model parameters that can most significantly influence the time course macrophage polarization profiles (see Transparent methods for more details) (Marino et al., 2008). From the results in Figure 6A, we see that the most influential parameters can be categorized into seven signaling modules described in our model, and based on that we then simulated a number of targeted interventions that are potentially feasible in experiments and assessed the resulting time course M1-M2 profiles. Among the STAT/IRF targets, we see that targeting STAT1 activation (through inhibition of its association with IFNGR, Figure 6F) or STAT6 (through inhibition of its deactivation, Figure 6I) can both lead to potent repolarization toward M2 (anti-inflammatory, pro-angiogenic) phenotypes, whereas the effect of targeting STAT3 (through inhibition of its deactivation, Figure 6H) or IRF1 (through increased degradation, Figure 6G) or inhibiting STAT1 dimerization (Figure S11D) is either less ideal or ineffective. Enhancing HIF1/2α expression (through inhibition of PHD activities, Figure 6E) or AKT activation (Figure S11E) is also suggested to be ineffective overall as both strategies fail to downregulate the pro-inflammatory M1 response, although they are able to enhance the production of anti-inflammatory and pro-angiogenic factors. Interestingly, our simulations showed that inhibition of SOCS1 (through increased degradation, Figure 6D) could also be an effective strategy to drive the M1-to-M2 repolarization under HSS, which is not readily apparent given the negative feedback function of SOCS1 in both canonical M1 (e.g., LPS) and M2 (e.g., IL-4) signaling, but this prediction is in agreement with the finding from a previous study showing that SOCS1 silencing can increase the ratio of anti-inflammatory to pro-inflammatory features in polarized macrophages (Whyte et al., 2011). At the level of cytokines, direct inhibition of endogenous IFNγ production as well as promotion of VEGF (the pro-angiogenic isoform) or IL-10 production are all suggested to effectively induce M1-to-M2 repolarization (Figures S11F–S11H). The predicted effect of targeting IL-10 to improve M2-like macrophage polarization and potentially ischemic tissue perfusion has been recently confirmed in a mouse model of PAD (Gotze et al., 2020).

We also performed the same set of analyses for another potential model representation of HSS (hypoxia plus reduced protein and RNA synthesis, see Transparent Methods for more details) (Zetterberg and Skold, 1969), and the overall results (Figure S11A) were similar to the hypoxia-only representation. In addition to sensitivity analysis, we performed model uncertainty quantification using the bootstrap method (see Transparent methods for more details) with a focus on the identifiability of a set of most influential parameters. The results suggested relatively robust clustering of these parameters against our calibration datasets (Figure S12).

### *In silico* single-cell analysis of model-based virtual macrophages

**Figure 7:**
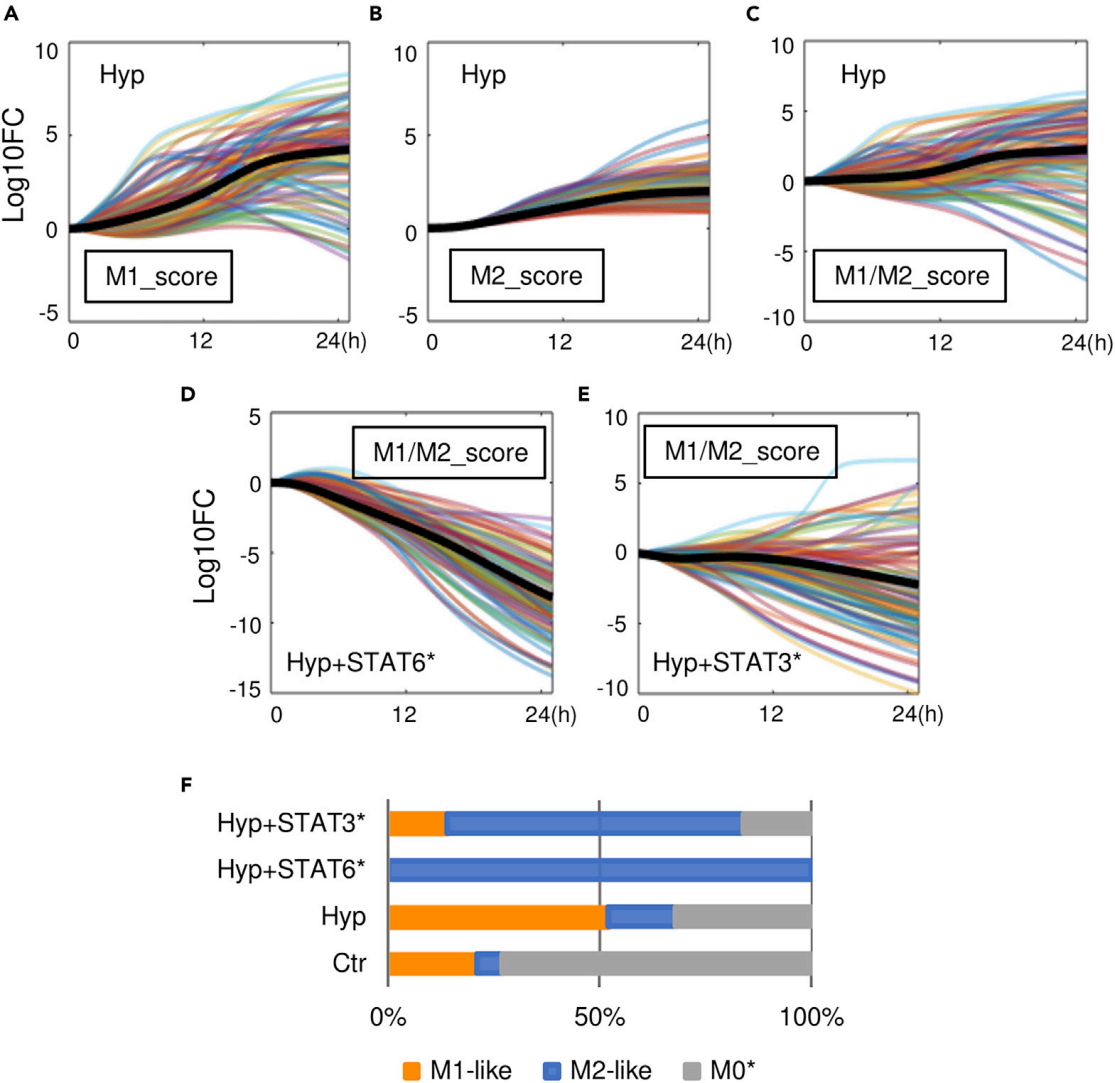
*In silico* single-cell analysis of model-derived virtual macrophage populations (A–E) Simulated time course responses of 100 model-generated virtual macrophages in terms of (A) relative M1 scores and (B) M2 scores under hypoxia (2% O_2_); (C) relative M1/M2 scores under hypoxia, (D) under hypoxia plus intervention targeting STAT6 (dephosphorylation rate x0.1), and (E) under hypoxia plus intervention targeting STAT3 (dephosphorylation rate x0.1). (A–E) Results are normalized to the respective values at t = 0 and then log10 transformed (y axes). Black bolded lines are the trajectories of the reference model. (F) Percentages of M1-like, M2-like, and insignificantly polarized cells (M0∗) under the above simulated conditions evaluated at 24 h (detailed definitions of M1-like, M2-like, and M0∗ are described in Transparent methods). Ctr, control/untreated condition; Hyp, hypoxia.

To further explore the variability in the macrophage polarization spectrum, we generated 100 digital alternative versions of our model (using the method described in Transparent methods), although each alternative version can be considered as an individual macrophage with consistent mechanisms and physiology but different innate biochemical reaction rates compared with the others, to approximate physiological cell-to-cell heterogeneity within a general macrophage population (Altschuler and Wu, 2010). We simulated this virtual macrophage population under hypoxia and observed a widespread spectrum of overall M1-M2 phenotypes, which are not uniform in terms of polarization intensities or directions (Figures 7A–7C), and such diversity likely originated from variabilities in both the intermediate signal transduction cascades (Figures S13A–S13F) and the downstream mechanistic regulation of M1-M2 markers (Figures S13G–S13J) in each individual cell. The impact of such a spectrum-like response is more evident under scenarios of simulated therapeutic interventions: although inhibiting STAT6 or STAT3 deactivation (as discussed in the previous section) under hypoxia can both induce apparent M1-to-M2 repolarization in an average macrophage (e.g., the reference model behavior in bold black lines in Figures 7D and 7E), in the simulated population only a portion of the cells would respond and be repolarized to the M2-like phenotypes upon STAT3 targeting, whereas almost all cells were successfully polarized to the M2-like phenotype transition upon STAT6 targeting (Figures 7D–7F). This simulation-derived phenomenon of partial response due to cell-level macrophage heterogeneity could have important implications for therapies that aim to treat diseases by modulating macrophages in the microenvironment (e.g., targeting tumor-associated macrophages to treat cancer), as the remaining macrophages that are unresponsive to targeted therapies may still possess sufficient functions to drive disease progression and hamper the overall treatment efficacies, and this proposed idea is supported by a recent study that examined macrophage-targeted therapies in colon cancer (Zhang et al., 2020). Furthermore, based on the simulation of our virtual macrophage population, we find that a small portion of cells under control condition (no external stimulation) can already exhibit M1-or M2-like phenotypes, whereas most cells are at unpolarized states (Figure 7F), and this phenomenon has also been confirmed *in vitro* by recent single-cell studies (Muñoz-Rojas et al., 2020; Li et al., 2019). In summary, the simulations presented here suggest that our systems-level model can be exploited in highly flexible and efficient ways to enable *in silico* investigation of macrophage polarization from the single-cell perspective, while offering extra mechanistic insights regarding its temporal, dose-dependent, and quantitative features.

## Discussion

We have here developed and presented a novel large-scale mechanistic computational model that enables, for the first time, a systems-level description (from quantitative, temporal, dose-dependent, and single-cell perspectives) of macrophage polarization under a complex M1-M2 multi-pathway network. Compared with previous models on this topic (Figure S1), the comprehensive model calibration and validation we implemented using an unprecedentedly large amount of experimental data further allows us to probe into and predictively simulate several essential features that constitute the spectrum-like physiology of macrophage polarization, such as its integrative multi-pathway signal transduction and feedback, multi-modal transcriptional and post-transcriptional target regulation, dynamic production of phenotype markers, and fine-tuned self-modulation via autocrine signaling. Although in our analyses we selected an *in vitro* experimental condition of PAD to demonstrate model utility and its potential translational application, the mechanistic setup of our model in terms of the pathways and mechanisms included is equally important for macrophages under general as well as other disease-specific scenarios. Still, the model certainly does not account for the full biology (an innate limitation even for large-scale models), as it is practically infeasible, due to relatively limited experimental data and high computational cost, to detail and calibrate every known pathway and mechanism that regulate macrophage polarization within a single modeling study while maintaining a high degree of model performance accuracy (Frohlich et al., 2018; Bouhaddou et al., 2018; Schmiester et al., 2020). Thus, our current model and its formulation can instead serve as a high-quality mechanistic computational platform that can be accordingly and continuously expanded and enriched with additional pathway details to further investigate macrophage functions in specific disease areas of interest, e.g., TLR pathways in various infectious disease settings (O'neill et al., 2009), CD47/SIRPα axis in macrophage-mediated cancer immunotherapy (Weiskopf, 2017), and cellular metabolic pathways in nonalcoholic fatty liver disease (Oates et al., 2019).

As our model was formulated and calibrated based on a large number of experimental data derived from independent studies using different macrophage cell lines, a major limitation is the unifying assumption we made that these data all come from consistent *in vitro* differentiation and culture conditions before stimulation and that all data were used to model the behavior of an “average” macrophage culture. In fact, technical differences in macrophage culture protocols can notably influence the resulting macrophage behaviors. It has been shown that GM-CSF and M-CSF, two commonly used factors in the experimental differentiation of macrophages from monocytes, can differentially regulate M1-M2 marker expression at baseline and also predispose macrophages to enhanced M1 and M2 responses, respectively, when further challenged by other stimuli (Hamilton et al., 2014; Fleetwood et al., 2007). Different concentrations and durations of PMA (phorbol 12-myristate-13-acetate), which is widely used to condition the human monocytic THP-1 cells, also results in wide variations of downstream protein expression and ultimately nontrivial differences in their polarization responses (Pinto et al., 2020). In addition, innate genetic differences between human and mouse macrophage cell lines (Thomas and Mattila, 2014), between cell lines of human origin (Mendoza-Coronel and Castanon-Arreola, 2016; Shiratori et al., 2017), and between mouse cell lines derived from different strains (Santos et al., 2006) can all lead to differential macrophage phenotype outcomes (e.g., inconsistent marker expression patterns) when these cells are treated with the same stimulation. While this evidence may suggest that the qualitative instead of quantitative aspects of certain model predictions would be more meaningful when tested experimentally across cell lines, our mechanistic model formulation (compared to other logic-based models) can in fact readily incorporate cell line-specific genomic and proteomic data, if available, to simulate the spectrum of response driven by those innate differences (as partially reflected by our *in silico* single-cell analysis). Another limitation resulted from our unifying assumption is that the model does not explicitly consider any potential effects of serum starvation, a commonly used experimental protocol, in its simulations. Although serum composition is poorly defined and also highly variable across studies, which makes it a possible factor that could complicate experimental results and reproducibility, serum depletion/starvation, apart from being a procedure to eliminate that factor, has been also shown to alter basal signaling, transcriptional patterns, and energy metabolism in several cell types, and the combinations of serum starvation with hypoxia or low glucose are considered appropriate *in vitro* models for certain ischemic diseases and tumors (Pirkmajer and Chibalin, 2011; Williams et al., 2016; Zheng et al., 2016; Golpour et al., 2014). As a complete mechanistic description of serum/starvation may be inherently infeasible, future efforts can focus on the most significant serum starvation-induced signaling and metabolic changes in macrophages, using semi-mechanistic or phenomenological methods, to better model its impact when more data becomes available. Another technical limitation is about the uniqueness of model parameterization. Although the large amount of calibration data and initial condition passing criteria we used have managed to confine the value distribution of the high-sensitivity parameter subset, it is still possible that our full model parameterization is associated with practical unidentifiability to some extent, as the parameter space has not been comprehensively explored here due to the limited degree of freedom allowed in the uncertainty analysis.

The model-based macrophage polarization maps under simulated *in vitro* and *in vivo* conditions revealed a wide space of heterogeneous macrophage functional phenotypes in terms of the dynamic expression and activation (e.g., up/downregulation, expression intensities) of an array of pro- and anti-inflammatory markers as well as transcription factors. This is based on the reasoning that macrophage functions cannot be reliably defined by only one or two phenotype markers, as these individual markers can likely be induced under multiple conditions, and that markers with opposing functions (e.g., M1 versus M2) may also co-express in non-mutually exclusive manners in the polarization spectrum (both observations were reflected in our simulations). Indeed, reliability and reproducibility of these individual markers has been a long-time issue in the field and more experimental studies now have shifted from the single-marker approach (e.g., iNOS versus ARG1, CD86 versus CD163) to the multi-marker approach that encompasses M1-M2 markers of different classes (e.g., cytokine, chemokine, intracellular protein, surface receptor, transcription factor) to better correlate phenotypes with the pleiotropic regulatory functions of macrophages (Murray et al., 2014; Gerrick et al., 2018; Chazaud, 2020; Jayasingam et al., 2019). In addition to the phenotype heterogeneity driven by complex combinations of stimulating signals, our model demonstrated that such heterogeneity could also occur at the single cell-level response even under a uniform polarizing condition. This could be highly relevant for the *in vivo* functional interpretation of macrophages under physiological and pathological settings, as they naturally exist in a continuum of functional states possibly due to the presence of diverse signals that can vary spatiotemporally in the microenvironment as well as normal cell-to-cell variations in gene and protein expression (Martins et al., 2017; Das et al., 2015; Jayasingam et al., 2019). Therefore, it is also of significant research and translational values to integrate mechanistic cell-level macrophage models (like ours) into higher-level tissue and whole-body scales to systematically simulate and investigate molecular-level target regulation, pathway-level signal transduction, and dynamic cell-cell communications that integratively define the underlying biological processes behind the macroscopic macrophage-environment-disease linkages (Martin et al., 2016; Sove et al., 2020; Norton et al., 2019). Interestingly, a recent modeling study by Cess et al. has in fact incorporated a previous cell-level macrophage model developed by us (which was the basis for the model presented here) into their multiscale system to simulate cell-cell interactions within the tumor microenvironment and has provided mechanistic insights regarding the optimal modalities of macrophage-based immunotherapies against tumors (Cess and Finley, 2020). In summary, the development of our model marks a significant step forward in the data-driven systems biology representation of the “virtual macrophage” concept through which heterogeneous datasets and new discoveries can be continuously and efficiently integrated to enable mechanistic, systems-level investigation of novel emergent properties in macrophage polarization and physiology.

### Limitations of the study

Several limitations of the study, including the assumption used in model development that all calibration data were derived from the same culture conditions, the simplification regarding the effects of serum and serum starvation during simulation, uniqueness of model parametrization, and the completeness of model biology, have been discussed in the Discussion section. In addition, it should be noted that although the model can efficiently simulate the dynamic and quantitative aspects of macrophage polarization in terms of the response of individual phenotype markers and a high-level metric (M1/M2 score), there is still a link to be made between the model-generated marker phenotypes and the ultimate functional phenotypes in terms of how macrophages actually regulate other cells in inflammation and angiogenesis. For example, the net regulatory effect of polarized macrophages (driven by a stimulus) on endothelial cell proliferation and tube formation would be a key readout of the macrophage functional phenotype in angiogenesis, although the correlation between this experimental readout and model-simulated marker response still remains to be mechanistically elucidated by future studies through a combination of experimental and computational approaches. For the exploratory *in silico* single-cell analysis that we performed using this model, although we generated a number of virtual single cells based on some mechanistic aspects of cell-to-cell heterogeneity and these cells can together produce a reasonable distribution of response to reflect certain physiological features, future efforts shall further refine the methodology used to formulate model-based single cells so that such virtual cells can be enriched with quantitative inputs from experimentally measured single-cell RNA and protein data, to better enable generation of model predictions with high translational values.

### Resource availability

#### Lead contact

Further information and requests for resources and data should be directed to the Lead Contact, Chen Zhao (czhao22@jhmi.edu).

#### Materials availability

This study did not generate new unique reagents.

#### Data and code availability

All relevant data are described within the manuscript and its Supporting Information files. Details of all model reactions, equations, parameters, initial conditions, and data are summarized in Table S1. Complete list of model reactions and parameter values; related to figure 1, Table S2. Differential equations and initial conditions of all model nodes; related to figure 1, Table S3. All quantitative data used in model calibration and validation; related to figures 2–4 and S2–S5. The complete model coded in SBML format (.xml file) and executable MATLAB scripts (.m files) that can run the model to generate sample simulations and analysis are provided in the Supplemental Information files.

## Methods

All methods can be found in the accompanying Transparent Methods supplemental file.

## Supplemental information

Document S1. Transparent methods and figures S1–S16

Table S1. Complete list of model reactions and parameter values; related to figure 1

Table S2. Differential equations and initial conditions of all model nodes; related to figure 1

Table S3. All quantitative data used in model calibration and validation; related to figures 2–4 and S2–S5

Data S1. Model SBML code and sample MATLAB scripts (compiled in a.zip file) for model setup, simulation, and analyses; related to figures 1–7

## Footnotes

Supplemental information can be found online at https://doi.org/10.1016/j.isci.2021.102112.
