# Supplementary material for: A data-driven computational model enables integrative and mechanistic characterization of dynamic macrophage polarization
Source: iScience. 2021 Jan 29;24(2):102112. doi: 10.1016/j.isci.2021.102112 (PMC7895754; doi:10.1016/j.isci.2021.102112)
Supplement: Document S1. Transparent methods and figures S1–S16 [file mmc1.pdf]

iScience, Volume *24*

Supplemental Information

A data-driven computational model enables integrative and mechanistic characterization of dynamic macrophage polarization

Chen Zhao, Thalyta X. Medeiros, Richard J. Sové, Brian H. Annex, and Aleksander S. Popel

Items included in this file:

Transparent Methods

Figure S1: Model specifics compared to previous mathematical multi-pathway models of macrophage polarization; related to Figure 1

Figure S2: Additional quantitative model calibration of pathway signal transduction (part 2); related to Figure 2

Figure S3: Additional quantitative model calibration of pathway signal transduction (part 3); related to Figure 2

Figure S4: Additional quantitative model calibration of pathway signal transduction (part 4); related to Figure 2

Figure S5: Additional quantitative model calibration of M1-M2 marker regulation (part 2); related to Figure 3

Figure S6: Constraining the model with additional qualitative experimental data; related to Figure 3

Figure S7: Macrophage polarization map under simulated *in vivo* stimulation conditions; related to Figure 5

Figure S8: Macrophage transcriptional regulation map under simulated *in vitro* and *in vivo* stimulation conditions; related to Figure 5

Figure S9: Experimental analysis of STAT signaling and HSS-induced M1-M2 marker regulation; related to Figures 2 and 6

Figure S10: Flow cytometry analysis of macrophage markers; related to Figure 6

Figure S11: Macrophage response under HSS and *in silico* targeted interventions to promote M2-like phenotypes; related to Figure 6

Figure S12: Parameter estimate distributions after bootstrapping; related to Figure 6

Figure S13: *In silico* analysis of hypoxia-driven transcriptional and marker regulation at the single-cell level; related to Figure 7

Figure S14: Raw data for qPCR analysis; related to Figure 6

Figure S15: Raw data for Western Blot analysis; related to Figures 2 and 6

Figure S16: Detailed model diagram; related to Figure 1

<u>Other supplemental items as individual files:</u>

Table S1 (Excel spreadsheet): Complete list of model reactions and parameter values; related to Figure 1

Table S2 (Excel spreadsheet): Differential equations and initial conditions of all model nodes; related to Figure 1

Table S3 (Excel spreadsheet): All quantitative data used in model calibration and validation; related to Figures 2-4 and S2-S5

Data S1: Model SBML code and sample MATLAB scripts (compiled in a .zip file) for model setup, simulation and analyses; related to Figures 1-7

Transparent Methods

Summary of Model Formulation, Simulation, Calibration and Analysis

Our mechanistic systems-level model was constructed based on ODEs with a total number of 166 model nodes (from the 67 "unique species") and 258 reactions (details regarding all reaction descriptions, equations, parameter values and initial conditions are summarized in Tables S1-S2). The general ideas and methodologies employed during model formulation (e.g. implementation of pathway structures, mechanisms of regulation, external and internal perturbations) follow a similar logic as reported in a previous modeling study from us (Zhao et al., 2019) and are described in more detail in the modular sections below. We used the same unit conversion method as described in (Zhao et al., 2019) to compute the corresponding molecular initial conditions for the model to simulate the different stimulus concentrations used in *in vitro* experiments. All model reactions and data (e.g. reaction rules of modeled species and nodes, parameter values, initial conditions) were compiled in MATLAB SimBiology Toolbox (MathWorks, Natick, MA) and the ode15s solver in MATLAB was used for model simulations. Model calibration was done at two levels: dynamic model behavior and model initial conditions. The rules we used for the initial condition calibration, in order to capture the quantitative states of unpolarized macrophages before any external driving stimuli or internal perturbations were applied, were designed based on the experimental measurements (compiled from literature, in terms of absolute concentrations or copy numbers) of the quantitative levels of the unique species modeled (all rules are summarized in Table S2). Calibration of dynamic model behaviors was implemented using global optimization (*patternsearch* function) in MATLAB. Full details of model calibration (for both dynamic behavior and initial conditions) are described in the "Model Calibration and Validation" section below. ImageJ software (NIH) was used for the blot densitometry analysis and other image measurements during quantification of experimental data. All quantitative data used in model calibration and validation were summarized in Table S3 (in addition to mean values, SEM or SD values are also included in figure displays if available). Model SBML code (in .xml format) and sample MATLAB scripts (.m files) for model simulation and analyses are also provided in Supplemental Information (Data S1 file) to ensure reproducibility

Sensitivity analyses were performed using the PRCC algorithm as described in (Marino et al., 2008) and in the "Model Sensitivity and Uncertainty Analyses" section below. For the primary outputs of interest in sensitivity analysis and in the analyses described in Figs.5-7 (and Figs.S7-8, S11-13), we introduced numerical metrics (M1-, M2- and M1/M2 scores) that consider major M1-M2 markers modeled to quantitatively characterize the relative dominance of M1 or M2 (pro-inflammatory or anti-inflammatory) phenotypes for stimulated macrophages. M1-score is the multiplication of the levels (e.g. protein/mRNA copy numbers) of 7 modeled pro-inflammatory markers iNOS, IL-12, IFNγ, TNFα, IL-1β, CXCL9 and CXCL10, while M2-score is the multiplication of 4 modeled anti-inflammatory markers ARG1, IL-10, IL-1Ra and VEGF (only the pro-angiogenic isoform VEGF165a is considered for M2-score). The overall M1/M2 score is the division of M1-score by M2-score and in the analyses they were further divided by the M1/M2 score under the baseline (control) condition to reflect directional regulation (for better display, the results were then log10 transformed, and more positive/negative would indicate more M1-/M2-like respectively). For the analyses in Figs.5B-C, simulated protein production rates instead of absolute levels of IL-1β, TNFα, IFNγ, IL-10 and VEGF (pro-angiogenic isoform) were used to calculate M1/M2 scores as these markers themselves were also stimuli (whose initial conditions would be manually modified to reflect *in vitro* and *in vivo* stimulation). For the categorization presented in Fig.7F, based on the number of markers we considered for M1 (7) versus M2 (4), we then assumed that log10 transformed relative M1/M2 scores greater than 2 and less than -1 would correspond to M1-like and M2-like respectively, while values in the remaining range (from -1 to 2) correspond to M0*.

Summary of In-house Experimental Data

Our experimental time-course expression data of pSTAT1/STAT1 and pSTAT3/STAT3 were used for model calibration; our experimental data of the response of iNOS, ARG1, CXCL9, TNFα, IL-1β, IL-10, TGFβ, SOCS3, FIZZ1 and CD80 under HSS were summarized in a qualitative manner and then compared with model simulations in Fig.6. The processed (and raw) experimental data for each individual gene/protein measured are shown in Figs.S9, S10, S14 and S15. Detailed experimental protocols are described in the sections below.

Cell Culture

Raw 264.7 murine macrophages were purchased (Millipore Sigma, Sigma-Aldrich, St Louis, MO, Cat # 91062702) and grown in standard Dulbecco's Modified Eagle Medium (DMEM) with 10% FBS (Thermo-Fisher Scientific, Cat # 11965092). Cells were exposed to hypoxia (2% oxygen, BioSpherix, Lacona, NY) with serum starvation (Cell Applications Inc, Cat # 209-250) to simulate ischemia *in vitro* or treated with 50 ng/mL of recombinant IFNγ (Shenandoah Biotechnology Inc., Cat # 200-16) or 10 ng/mL of recombinant IL-4 (Shenandoah Biotechnology Inc., Cat # 200-18) to induce M1 and M2 phenotypes.

RNA Isolation & Quantitative PCR

Total RNA was extracted using PureLink RNA Mini Kit (Ambion by Life Technologies, Cat # 12183025) according to manufacturer's protocol. cDNA was synthesized from 311.2 ng of RNA using SuperScript III first strand synthesis super mix (Thermo Fisher, Cat # 11752-250) according to the manufacturer's protocol. qPCR was performed using the Taqman Gene Expression Mastermix (Applied Biosystems), cDNA, and Taqman assay (Applied Biosystems) probes with FAM labels for murine TNF-α, TGF-β, IL-10, IL-1β, and CXCL9, and VIC labels for murine GAPDH and Rplp0 from Thermo-Fisher Scientific (Cat # Mm00443258_m1, Mm00237725_cn, Mm00039670_cn, Mm00434228_m1, Mm00434946_m1, Mm00186822_cn, Mm00725448_s1). Data were collected and analyzed using the Biorad CFX96 Real Time System with C1000 Touch Thermal Cycler instrument with GAPDH and Rplp0 as internal expression controls.

Western Blotting

Levels of target protein were analyzed by western blotting using antibodies to iNOS (BD Biosciences, Woburn, MA, Cat # 610432) and Arg1, pSTAT1, STAT1, pSTAT3 and STAT3 (Cell Signaling Technology, Danvers, MA, Cat # 93668s, 9167s, 14994s, 9145L, 12640s). Western blots were analyzed by Odyssey Infrared Imaging System (LI-COR Biosciences, NE).

Flow Cytometry

For flow cytometry staining, macrophages were dissociated from T75 flasks using cold PBS and ice for 0.5–1h. Then, about 10^6 cells were placed in individual 5mL round-bottom tubes (Falcon, Cat # 352058), stained with appropriate membrane antibody mixtures (CD80 FITC Biolegend Cat # 104705, F4/80 APC Fire 750 Biolegend Cat # 123151) or permeabilized with BD Cytofix/Cytoperm Fixation and Permeabilization Solution (BD Biosciences, Cat # 554722) and stained for intracellular markers with anti-SOCS3 (Biolegend, Cat # 626602) or anti-FIZZ1 (Novus Biologicals, Cat # NBP229355), washed with BD Perm/Wash buffer (BD Biosciences, Cat # 51-2091KZ) and finally stained with secondary antibodies (Invitrogen, Alexa-Fluor 488 Cat # A11001 and Alexa-Fluor 647 Cat # A21244). Percentages of parent events presenting target proteins were sorted by flow cytometry using BD FACSCanto Flow Cytometer (BD Biosciences, MA).

Statistical Analysis

Statistical analysis was performed with GraphPad Prism software. An unpaired t test was used for comparison between 2 groups, and comparisons in experiments with ≥3 groups were performed with one-way ANOVA. Statistical significance was set at $p<0.05$.

Additional Details of Model Formulation and Analysis

The 7-pathway model (including 6 receptor-mediated pathways and oxygen-sensing pathway) presented in this paper is a continuation of a 3-pathway model previously developed by our group (Zhao et al., 2019). Therefore, in a similar manner, the major mechanistic regulations and biochemical reactions in the current model were formulated based on findings in the literature (as shown in Figs.1-4 and S2-S6) and also certain assumptions we made. In the below sections, we describe additional details in the formulation and implementation of several model components that were not explicitly discussed in the calibration and validation sections (a complete list of all species and reactions can be found in Tables S1-S2). We also elaborate further on the mathematical methodologies used in the analyses of our simulation results.

Activation/Deactivation Cycle of STATs

The STATs (STAT1, STAT3, STAT6) in our model follow a canonical activation and deactivation process: monomer phosphorylation, dimerization of phosphorylated monomers (reversible), dimer translocation to nucleus, dimer deactivation (e.g. dephosphorylation) in nucleus, dissociation of deactivated dimers into monomers in nucleus, monomer export from nucleus to cytoplasm (in addition, phosphorylated monomers can also be deactivated in cytoplasm) (Mitchell and John, 2005). We also modeled the mutual sequestration between STAT1 and STAT3 that phosphorylated STAT1 and STAT3 monomers can form a heterodimer (reversible), which can then translocate to the nucleus but is nonfunctional (according to our assumption) so the heterodimers would later be deactivated and broken down to STAT1 and STAT3 monomers. Such a mutual sequestration phenomenon has been reported as a potential mechanism to limit the signaling of STAT1 by STAT3 (and vice versa) (Delgoffe and Vignali, 2013; Ho and Ivashkiv, 2006). For the interactions between STAT1 and IRF9, we assumed that activated STAT1/IRF9 complexes in nucleus can be formed in two ways: by the association (reversible) between IRF9 and activated STAT1 dimers in nucleus, and by the same reactions happening in cytoplasm followed by nuclear translocation (Suprunenko and Hofer, 2016). The activated STAT1/IRF9 complexes can also be deactivated and dissociate in the nucleus (assumed to be a one-step process that gives IRF9 proteins and deactivated STAT1 dimers).

Receptor/Ligand Kinetics

All receptors in the model were regulated by constitutive production and degradation, in addition to ligand-mediated internalization and degradation. Bound ligand-receptor complexes that were internalized can enter pre-degradation states (along with the dissociation of other accessory proteins, e.g. JAK, SOCS) and then undergo either degradation of both ligands and receptors, or degradation of ligands only and recycling of the receptors (Casaletto and McClatchey, 2012). Among the 6 receptor-mediated pathways in the model, only IL-4 signaling was shown to require receptor internalization (Kurgonaite et al., 2015; Cendrowski et al., 2016; Wei et al., 2006; Hansen et al., 2013; Blouin and Lamaze, 2013; Green et al., 2017).

JAK proteins (e.g. JAK1, JAK2, JAK3) were shown to be associated with different cytokine receptors in a partially redundant manner, so we made a simplification and merged them together into one JAK species that binds all IL-4/IL-10/IFNγ receptors (O'Shea et al., 2015). In our model, the majority of IL-4, IL-10 and IFNγ receptors were pre-associated with JAK proteins before binding their respective ligands (Marchetti et al., 2006). JAK can also be targeted by SOCS proteins (e.g. SOCS1, SOCS3) for direct degradation (Croker et al., 2008).

In our model, the quantitative copy numbers of ligands in different stimulation conditions were calculated using their respective doses (e.g. in ng/ml) found in literature studies and molecular weights (e.g. in kilodalton) together with the assumption that every 10^6 cells share 1 mL of culture media *in vitro* (Zhao et al., 2019). Hypoxia was simulated as variations in the O_2 initial conditions by assuming that an O_2 concentration of 200 μM (then converted to absolute copy numbers) reflects normoxia (21% O_2) (Tuckerman et al., 2004). Apart from the 6 activating ligands (IL-1β, IL-4, IL-10, IFNγ, TNFα, $VEGF_{165}a$) in the model, the autocrine effect of IL-12 (modeled as one single species instead of a heterodimer) was implicitly captured in terms of its ability to upregulate IFNγ production (Munder et al., 1998). In addition, there were two ligands that potentially act as signal inhibitors in the model. IL-1Ra can bind to IL-1 receptors but cannot induce any downstream signaling or receptor internalization (Dripps et al., 1991). For $VEGF_{165}b$, it was assumed that it binds VEGFR1 and induces receptor internalization, degradation and recycling (similar to $VEGF_{165}a$) (Boucher et al., 2017); however, binding of $VEGF_{165}b$ would not induce any signaling events. We also assumed that only $VEGF_{165}a$ (but not $VEGF_{165}b$) production is directly upregulated by hypoxia through HIFs (Varey et al., 2008). When 'VEGF' is mentioned in the manuscript as a stimulus, it refers to pro-angiogenic isoforms only unless otherwise noted; when 'VEGF' is mentioned in the manuscript as a product, it refers to the sum of both pro-angiogenic and anti-angiogenic isoforms.

AKT Isoforms

In our model, we included two isoforms of AKT, AKT1 and AKT2. Both isoforms can be activated by PI3K and were assumed to regulate downstream targets in a similar manner, except for miR-155 as AKT1 inhibits miR-155 production and AKT2 has the opposite effect (Arranz et

al., 2012). To compare with literature results where only a single AKT species was considered, the sum of activated (e.g. phosphorylated) AKT1 and AKT2 was used.

IRFs

IRF1, a master regulator downstream of the IFNγ/STAT1 axis, was assumed to be the essential effector (instead of STAT1, as suggested by optimization results) for two IFNγ-regulated effects: inhibition of miR-3473b and IL1R production (Wu et al., 2014; Hu et al., 2005). In our model, the expression levels of IRF1, IRF4 and IRF9 can be up- and down-regulated by external stimuli to reflect activation and repression, while the level of IRF5 was assumed to be constant so IRF5 was dynamically regulated between inactive and active states (Ren et al., 2014). The inhibitory effect of IRF4 on IRF5 was modeled as an IRF-4-mediated decrease in the rate of IRF5 activation (Negishi et al., 2005). Another well-studied IRF member, IRF3, was not included here as it is not significantly regulated by any of the model pathways (Endo et al., 2014; Wang et al., 2016).

MAPKs

In our model, the life cycles of three MAPKs (p38, ERK, JNK) followed a simple canonical cascade: activation (e.g. by phosphorylation) in cytoplasm, translocation of activated MAPKs to nucleus, deactivation (e.g. by dephosphorylation) of activated MAPKs in nucleus and export to cytoplasm (merged into a one-step reaction). In addition, activated MAPKs can also be deactivated directly in cytoplasm. We also assumed that their regulatory functions are only determined by the levels of activated MAPKs in nucleus (Plotnikov et al., 2011). One of their major downstream targets in the model, transcription factor AP-1, was simplified as one single species (instead of protein dimers) whose production was jointly regulated by the three MAPKs (Lopez-Bergami et al., 2010). For the activation of ERK in response to IL-4 and IL-10, we semi-mechanistically introduced two species (named INT1/2, refer to Table S1 for more details) to represent the coarse-grained signal transduction processes between respective receptor ligation and downstream ERK activation, and this has significantly improved the model calibration.

Protein Production, Secretion and Degradation

For most species (e.g. those whose mRNAs were not explicitly modeled), protein production (and subsequent secretion, if applicable) is simplified as a one-step process. All species (e.g. mRNA, protein) whose expression can be induced in the model have a corresponding degradation reaction (mostly intracellular). Among all the secreted M1-M2 markers modeled, we assumed that CXCL9, IL-12 and IL-1Ra only undergo extracellular degradation; the other secreted M1-M2 markers only undergo ligand-mediated internalization and subsequent degradation.

Hypoxia Serum Starvation (HSS)

HSS was simplified as hypoxia only (2% O_2) in our primary model analyses in Fig.6. Additional analyses were also performed for an alternative simplified representation which is hypoxia plus 25% reduction in all RNA/protein synthesis rates (denoted as HSS* in Fig.S11) based on findings from (Zetterberg and Skold, 1969).

Model Calibration and Validation

Model calibration was done in a combination of manual tuning and computer optimization. We first searched the literature and were able to derive values (from both experimental data and published models) for a subset of the model parameters; for the remaining ones without literature reference, we put in tentative values to start with. Then we manually tuned the model parameter space until the model simulations achieved good visual agreements with all the corresponding calibration datasets simultaneously (the data are summarized in Figs.2-3 and S2-S5). In the meantime, we ensured that the initial conditions of all "unique" species in the model stayed in quantitative predefined ranges as estimated from literature (Table S2); we also ensured that the time-course levels of all model nodes with initial conditions greater than 20 did not display significant fluctuations (within $\pm$15% from the initial condition) over a span of 10000 minutes (~7 days) under the resting (no-treatment) condition, which were meant to indicate that these unpolarized, untreated macrophages in culture have achieved a relative equilibrium.

We then performed preliminary sensitivity analysis using the PRCC algorithm under seven stimulation conditions (e.g. by the 7 stimuli one at a time) and selected the top-ranked free parameters (e.g. those with absolute PRCC indices larger than 0.1) from each run. This gave a total of 80 parameters, which were then input as parameters to be optimized into a global optimization procedure in MATLAB (using *patternsearch* function, with 0.5x-2x as the allowed ranges for parameter value variations) with respect to all the calibration datasets presented in this paper. The objective function to be minimized in optimization is the weighted sum of squared errors (computed from the differences between simulated and experimental values, and normalized by the respective SD/SEM when applicable). For the weighting of the experimental data, individual time-course (and dose response) data series from macrophages was given a weight of 2 (1 for data series from non-macrophages); individual single-timepoint data from macrophages was given a weight of 1 (0.5 for single-timepoint data from non-macrophages). The computer optimization process also checked the initial condition bounds (using a similar method as described in (Zhao et al., 2019) but this time initial conditions were evaluated at 10000, 50000 and 100000 minutes of simulation under no-treatment condition to find any set of values that can satisfy the bounds) and fluctuation bounds at every iteration. The final parameter values generated by MATLAB were then rounded to 5 significant digits.

Overall, there are two main reasons that we chose this two-step approach (manual tuning then computer optimization) over the *patternsearch* only approach for model calibration and optimization. First reason is that the compilation of all calibration data from many disparate sources is a continuous process done over many months, given the large scope of model and the large number of data potentially available in the literature from different sources (instead of in one or two curated databases which there were none). Thus, if we were to do a new round of *patternsearch* every time we found new data, the overall process would be infeasible and would cost significantly more time. Second reason is that our model has a unique feature which is its background nonzero initial condition to represent unpolarized macrophages with autocrine signaling (plus the "fluctuation" constraint as mentioned above), and *patternsearch* only with unselected large starting ranges seems to perform very poorly on this, which may be due to many reasons, e.g. too many long-range autocrine feedbacks in the model that influence the initial conditions, poorly defined starting ranges.

The dataset used in model validation (Fig.4) was compiled in the following ways. If at least two cases (e.g. different stimulation doses) of “A regulates B” have been included in calibration, then a third case of “A regulates B” (e.g. stimulated at higher dose, or dose response curve) will be included in the validation set (single literature sources that contain multiple pieces of qualified data were also preferentially selected). In addition, three scenarios of combination treatment were included in the validation set. All data in the validation set were obtained from macrophage cell lines, while a small portion of data in the calibration set were derived from non-macrophage cell lines.

Generation of in silico Macrophages

We generated 100 digital alternative versions of our model (“*in silico* macrophages”) to represent the diversity in the macrophage phenotype response at the single-cell level. This is under the assumption that each individual macrophage can be educated only by the secreted signals produced by itself (e.g. autocrine effect only, no paracrine effect). For these 100 *in silico* macrophages, we used the parameters obtained from model calibration (e.g. the reference model) as the basis and further varied the production/degradation (or activation/deactivation) of 5 selected pathway regulators (RIP1, IRF1, IRF4, TRAF6, SOCS1) and 5 autocrine cytokines (TNFα, IL-1β, IFNγ, IL-10, $VEGF_{165}$a). To do that, we varied the Hill constants or normalization factors (randomly within the range of 0.1x-10x) used in the production/degradation (or activation/deactivation) of these molecules and then computed the new rates so that their overall resting state reaction fluxes are the same as in the reference model. Then these 100 *in silico* macrophages were simulated using the same initial conditions under various stimulation conditions as described in the manuscript main text.

Model Sensitivity and Uncertainty Analyses

Model sensitivity analyses were performed based on the algorithm and code as published in (Marino et al., 2008). For the algorithm settings, we used Latin Hypercube Sampling (LHS), p=0.05 as the cutoff for statistical significance, 5000 iterations for each run, evaluation time integral of t=0 to 24 h, and 0.5x-2x as the allowed ranges for parameter value variations (0.2x-5x

yielded similar results). The M1/M2 score, which is the multiplication (in terms of time integrals of relative fold changes) of 7 M1 markers ([iNOS]*[IL12]*[TNFα]*[IFNγ]*[CXCL9]*[mCXCL10]*[IL1β], M1 score) divided by the multiplication of 4 M2 markers ([ARG1]*[IL10]*[V165a]*[IL1RA], M2 score), was chosen as the output of interest for all PRCC calculations (and also other analyses in this study). During the preliminary sensitivity analysis (as described in the Calibration/Validation section), when the stimulus itself is a marker, then in that particular run of sensitivity analysis this stimulus was removed from the calculation of M1/M2 score. For the results displayed in Fig.6 in main text, we performed sensitivity analysis first (as described above) and then removed the parameters that directly control the production/degradation of single markers that has no autocrine mechanisms in the model, since targeting these processes are less meaningful as they can only regulate one out of the many macrophages response markers; parameters that are Hill constants were also removed for similar reasons.

For uncertainty analysis, we selected the top-ranked free parameters from seven runs (10 parameters were selected from each run) of sensitivity analyses (under the 7 stimuli, one at a time) and this collective set contained 25 distinct parameters after removing the duplicates. The complete calibration datasets were resampled 100 times. For each datapoint in the calibration datasets, we assumed that its value during resampling would randomly fall within a normal distribution with the mean and standard deviation (or standard error, if applicable) values that we directly obtained from the corresponding literature study. And for datapoints with only mean values available, we assumed that their standard deviations equal to 10% of the mean values. Finally, the 100 resampled datasets were fed into the optimization algorithm (as described above) to obtain 100 sets of new parameter estimates; during bootstrapping, parameter values were allowed to vary from 0.1x to 10x (of their reference values). The final readout of the uncertainty analysis is the relative value distribution of these 25 parameters.

Figure S1

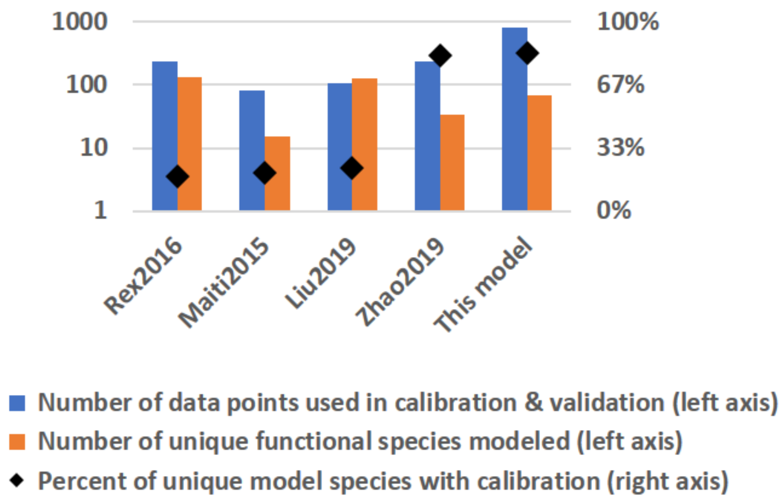

Figure S1. Model specifics compared to previous mathematical multi-pathway models of macrophage polarization; related to Figure 1. Compared to previous modeling studies on macrophage polarization (Rex et al., 2016; Maiti et al., 2015; Liu et al., 2019; Zhao et al., 2019), the current model was able to incorporate a significantly larger amount of quantitative experimental data (blue bars) for its calibration and validation, while it enables users to dynamically simulate a very high degree of mechanistic complexity in terms of pathway details, marker regulation and diverse application scenarios.

Figure S2

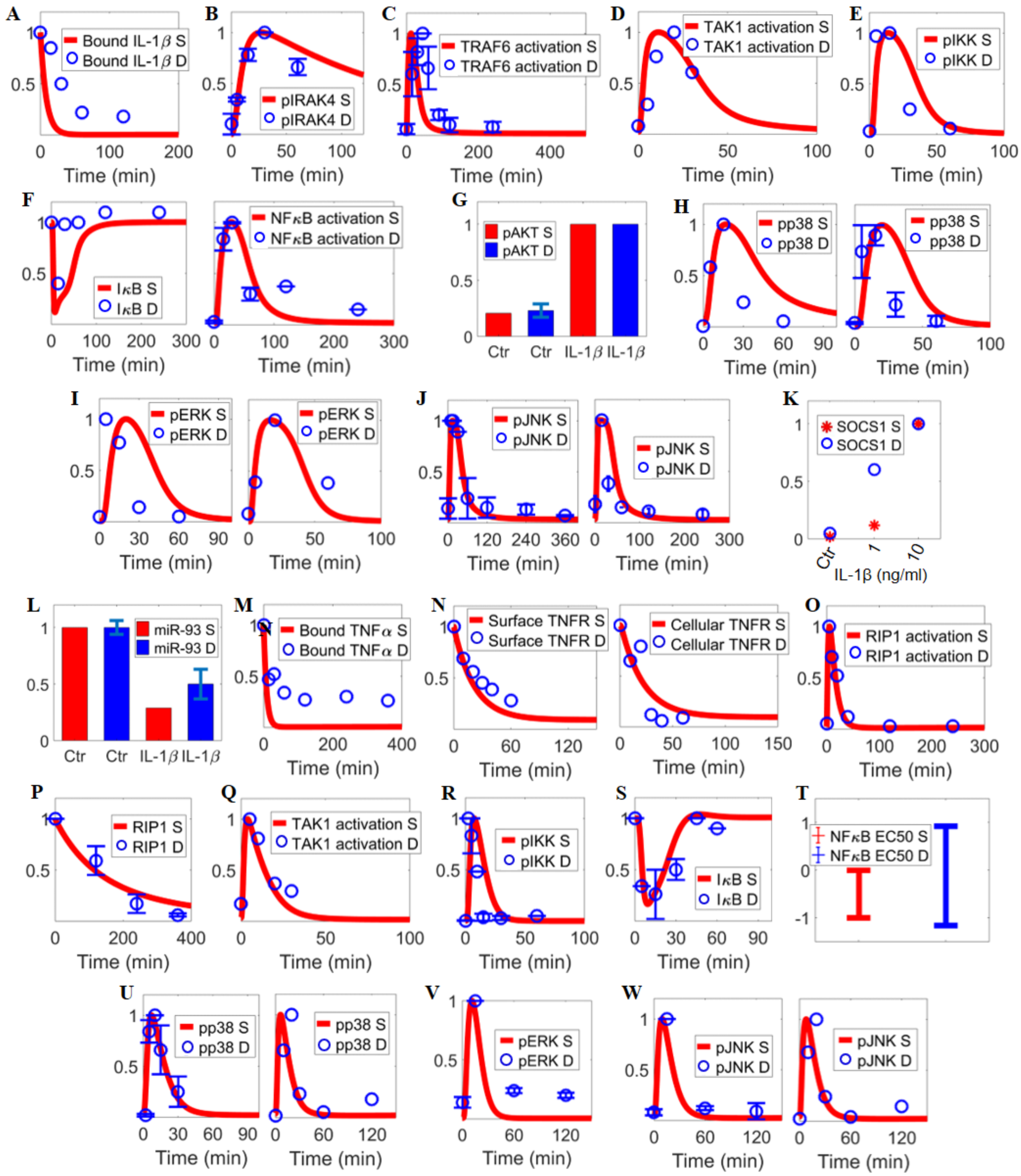

Figure S2. Additional quantitative model calibration of pathway signal transduction (part 2); related to Figure 2. Model simulations and corresponding experimental data are shown together (references are listed in the order of the data mentioned). (A) Cell surface receptor-bound IL-1β

undergoes internalization (Kilian et al., 1991). (B) IL-1β induces activation of IRAK4 (Vollmer et al., 2017), (C) TRAF6 (Shembade et al., 2010), (D) TAK1 (Cheung et al., 2003), (E) IKK (Funakoshi-Tago et al., 2009), (F) transient downregulation of IκB (Shembade et al., 2007) and subsequent NFκB activation (Funakoshi-Tago et al., 2009; Suzuki et al., 2002; Shembade et al., 2008), and (G) AKT activation (Neumann et al., 2002; Gulen et al., 2012). (H) IL-1β also induces activation of p38 (5 ng/ml (Vollmer et al., 2017) and 10 ng/ml (Suzuki et al., 2002; Funakoshi-Tago et al., 2009)), (I) ERK (10 ng/ml (Funakoshi-Tago et al., 2009) and 100 ng/ml (Hu et al., 2005)), (J) JNK (10 ng/ml (Suzuki et al., 2002; Shembade et al., 2008) and 20 ng/ml (Shembade et al., 2007)), and (K) upregulates SOCS1 (Choi et al., 2013); IL-1β can downregulate miR-93 expression (Xue et al., 2019). (M) Cell surface receptor-bound TNFα undergoes internalization (Imamura et al., 1987). (N) TNFα induces internalization (left) and degradation (right) of TNFR (Fischer et al., 2011). (O) TNFα induces activation of RIP1 (Shembade et al., 2010), (Q) TAK1 (Cheung et al., 2003), (R) IKK (Waterfield et al., 2004; Geng et al., 2017), (S) transient downregulation of IκB (Ermolaeva et al., 2008; Lo et al., 2011) and (T) dose-dependent activation of NFκB (simulated range of EC50 is within the experimental range EC50) (Trask, 2004). (P) Cellular expression of RIP1 is downregulated by A20 overexpression (simulated as 100x A20 initial condition) with cycloheximide treatment (simulated as 0x protein production rates) (Shembade et al., 2009). (U) TNFα also induces activation of p38 (40 ng/ml (Winston et al., 1997) and 100 ng/ml (Dzamko et al., 2012)), (V) ERK (Etemadi et al., 2015), and (W) JNK (20 ng/ml (Etemadi et al., 2015) and 100 ng/ml (Dzamko et al., 2012)). (A-W) All values are for protein levels unless noted otherwise and are normalized (y-axes are relative expression, except in T as described below). For normalization and display of results (simulation and data in A-W): A, IκB in F, L-N, P, S normalized to the respective t=0 values; G normalized to the maximum pAKT levels in response to IL-1β; y-axis in T is the log10 transformed TNFα concentrations in ng/ml; all others normalized to their respective maximum values. S–simulation, D–experimental data, Ctr–control/untreated condition.

Figure S3

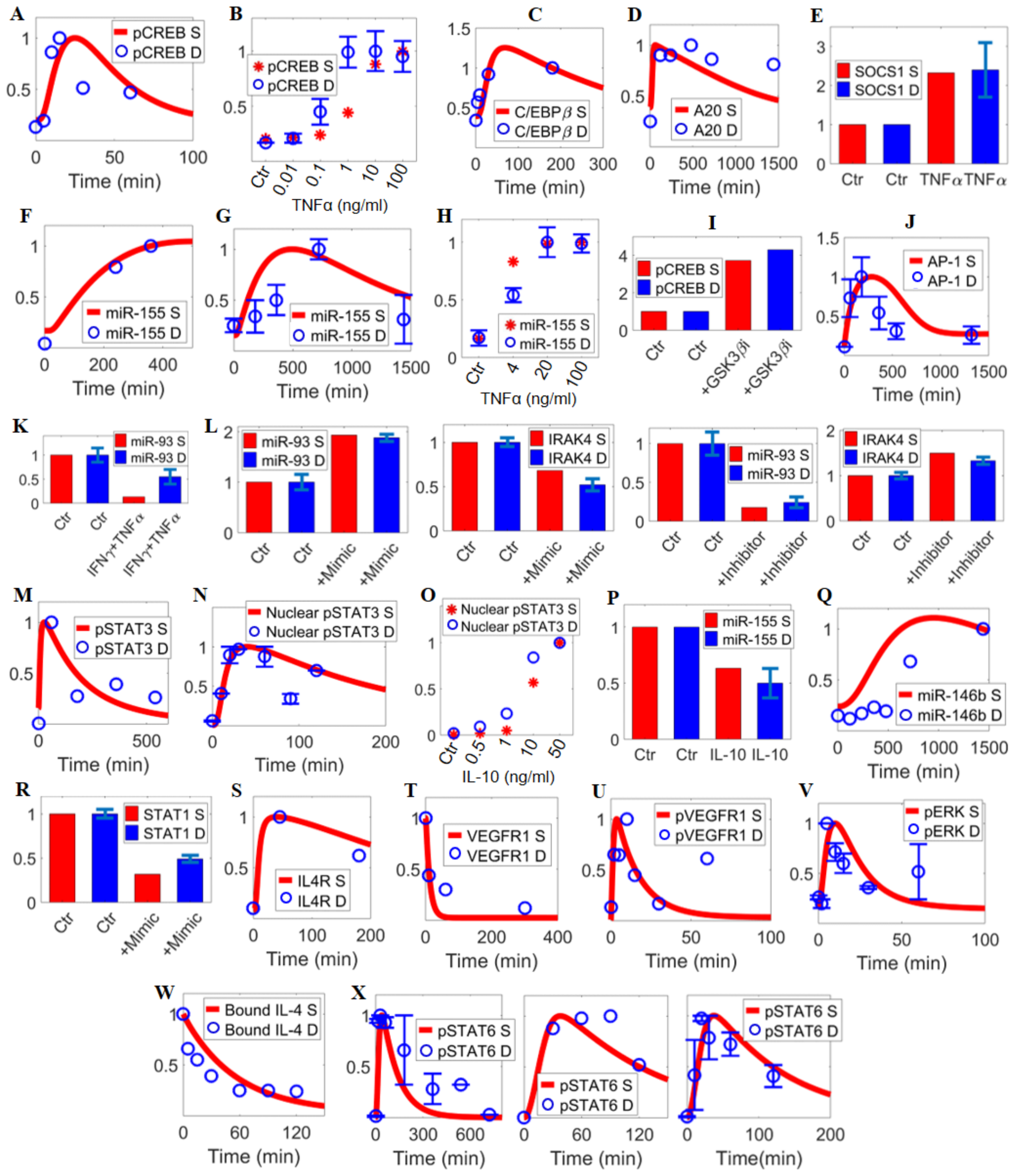

Figure S3. Additional quantitative model calibration of pathway signal transduction (part 3); related to Figure 2. Model simulations and corresponding experimental data are shown together (references are listed in the order of the data mentioned). (A-B) TNFα induces time-course (Koga et al.,

2016) and dose-dependent activation (Ono et al., 2006) of CREB. (C) TNFα induces expression of C/EBPβ (Yin et al., 1996), (D) A20 (Tran et al., 2009), (E) SOCS1 (simulated SOCS1 protein level is compared to SOCS1 mRNA data) (Ueki et al., 2004), (F-G) time-course (10 ng/ml (O'Connell et al., 2007) and 50 ng/ml (Migita et al., 2017)) and (H) dose-dependent expression of miR-155 (Migita et al., 2017). (I-J) Inhibition of GSK3β (simulated using the IC50 value from (Zhang et al., 2003)) results in activation of CREB and AP-1 (simulated AP-1 protein level is compared to c-Jun mRNA data) (Gotschel et al., 2008). (K) TNFα plus IFNγ treatments can downregulate miR-93 expression (Yee et al., 2017). (L) Overexpression ('mimic') of miR-93 inhibits IRAK4 and inhibition of miR-93 ('inhibitor') promotes IRAK4 expression (Tian et al., 2017). (M-N) IL-10 induces time-course (in whole-cell (Yasukawa et al., 2003) and nucleus (Niemand et al., 2003)) and (O) dose-dependent activation of STAT3 (Niemand et al., 2003). (P) IL-10 downregulates miR-155 (McCoy et al., 2010) and (Q) induces miR-146b (Curtale et al., 2013), and (R) miR-146b overexpression ('mimic') can inhibit STAT1 protein expression (He et al., 2016). (S) IL-10 also induces expression of IL4R (simulated IL4R protein production rate is compared to IL4R mRNA data) (Lang et al., 2002). (T) VEGF induces ligand-mediated VEGFR1 degradation (Kobayashi et al., 2004), (U) VEGFR1 phosphorylation and (V) ERK activation (Feliers et al., 2005). (W) Cell surface receptor-bound IL-4 undergoes internalization (Kawakami et al., 2002). (X) IL-4 (at 10 ng/ml (Covarrubias et al., 2016; Dickensheets et al., 2007; Yao et al., 2016), 20 ng/ml (Li et al., 2017) and 50 ng/ml (Rex et al., 2016; Kimura et al., 2016)) induces STAT6 activation. (A-X) All values are for protein levels unless noted otherwise and are normalized (y-axes are relative expression). For normalization and display of results (simulation and data in A-X): E, I, K, L, P, R, T, W normalized to the respective t=0 values; C, F, Q normalized to the respective values at the last experimental timepoints; all others normalized to their respective maximum values. S–simulation, D–experimental data, Ctr–control/untreated condition.

Figure S4

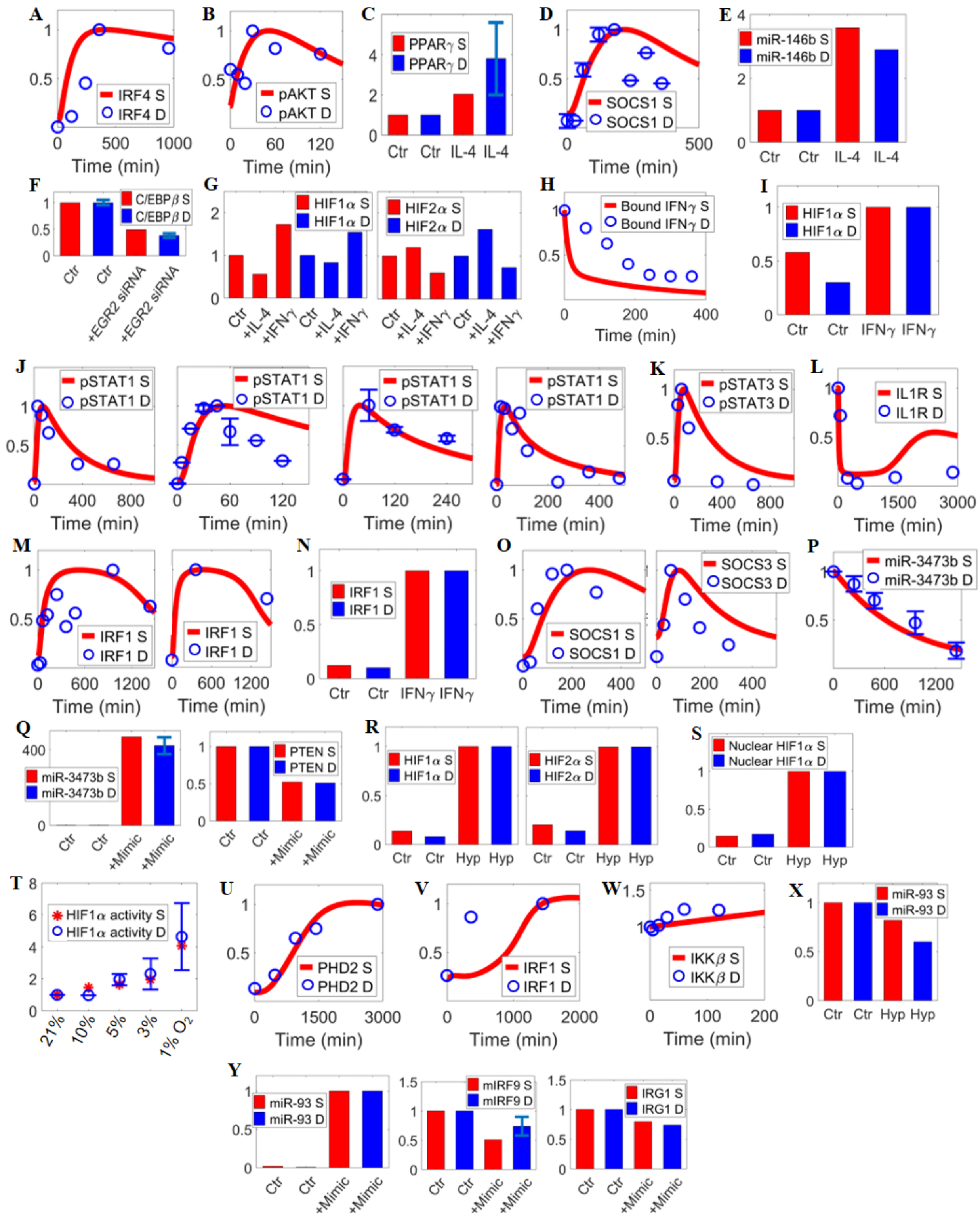

A
IRF4 S
IRF4 D
Time (min)
B
pAKT S
pAKT D
Time (min)
C
PPARγ S
PPARγ D
Ctr Ctr IL-4 IL-4
D
SOCS1 S
SOCS1 D
Time (min)
E
miR-146b S
miR-146b D
Ctr Ctr IL-4 IL-4
F
C/EBPβ S
C/EBPβ D
Ctr Ctr +EGR2 siRNA +EGR2 siRNA
G
HIF1α S
HIF1α D
HIF2α S
HIF2α D
Ctr +IL-4 +IFNγ Ctr +IL-4 +IFNγ
H
Bound IFNγ S
Bound IFNγ D
Time (min)
I
HIF1α S
HIF1α D
Ctr Ctr IFNγ IFNγ
J
pSTAT1 S
pSTAT1 D
Time (min)
K
pSTAT3 S
pSTAT3 D
Time (min)
L
IL1R S
IL1R D
Time (min)
M
IRF1 S
IRF1 D
Time (min)
N
IRF1 S
IRF1 D
Ctr Ctr IFNγ IFNγ
O
SOCS1 S
SOCS1 D
SOCS3 S
SOCS3 D
Time (min)
P
miR-3473b S
miR-3473b D
Time (min)
Q
miR-3473b S
miR-3473b D
PTEN S
PTEN D
Ctr Ctr +Mimic +Mimic
R
HIF1α S
HIF1α D
HIF2α S
HIF2α D
Ctr Ctr Hyp Hyp
S
Nuclear HIF1α S
Nuclear HIF1α D
Ctr Ctr Hyp Hyp
T
HIF1α activity S
HIF1α activity D
21% 10% 5% 3% 1% O2
U
PHD2 S
PHD2 D
Time (min)
V
IRF1 S
IRF1 D
Time (min)
W
IKKβ S
IKKβ D
Time (min)
X
miR-93 S
miR-93 D
Ctr Ctr Hyp Hyp
Y
miR-93 S
miR-93 D
mIRF9 S
mIRF9 D
IRG1 S
IRG1 D
Ctr Ctr +Mimic +Mimic

Figure S4. Additional quantitative model calibration of pathway signal transduction (part 4); related to Figure 2. Model simulations and corresponding experimental data are shown together (references are listed in the order of the data mentioned). (A) IL-4 induces IRF4 expression (Hsu et al., 2018), (B) AKT activation (Kimura et al., 2016), (C) expression of PPARγ (Kim et al., 2018; Date et al., 2014), (D) SOCS1 (simulated SOCS1 protein level is compared to SOCS1 mRNA data) (Dickensheets et al., 2007), and (E) miR-146b (Malik, 2016). (F) C/EBPβ is downregulated by EGR2 siRNA (implemented as 0.1x EGR2 production rate, simulated C/EBPβ protein level is compared to C/EBPβ mRNA data) (Veremeyko et al., 2018). (G) IL-4 and IFNγ selectively regulate HIF1α and HIF2α under hypoxia (Takeda et al., 2010). (H) Cell surface receptor-bound IFNγ undergoes internalization (Celada and Schreiber, 1987). (I) IFNγ induces HIF1α stabilization under normoxia (Takeda et al., 2010). (J) IFNγ (at 10 ng/ml (Kim et al., 2015), 20 ng/ml (Li et al., 2017), 50 ng/ml – original data as shown in Fig.S9A, 125 ng/ml (Wormald et al., 2006)) potently induces STAT1 activation. (K) IFNγ also induces STAT3 activation (Kim et al., 2015). (L) IFNγ represses production of IL1R (simulated IL1R protein production rate is compared to IL1R mRNA data) (Hu et al., 2005). (M-N) IFNγ (at 2.5 ng/ml (Vila-del Sol et al., 2008), 100 U/ml (Carta et al., 2001), 10 ng/ml (Liu et al., 2003)) induces IRF1 expression. (O) IFNγ upregulates SOCS1 and SOCS3 expression (simulated SOCS1/3 protein levels are compared to SOCS1/3 mRNA data) (Dickensheets et al., 2007). (P) IFNγ downregulates miR-3473b; (Q) overexpression (‘mimic’) of miR-3473b inhibits PTEN abundance (Wu et al., 2014). (R) Hypoxia stabilizes cellular HIF1α and HIF2α (Imtiyaz et al., 2010); (S) hypoxia promotes HIF1α activation in nucleus (Ramanathan et al., 2007) and (T) in dose-dependent manners (Cummins et al., 2006). (U) Hypoxia induces PHD2 expression (Berra et al., 2003), (V) IRF1 expression (Carta et al., 2001), and (W) IKKβ expression (Cummins et al., 2006). (X) Hypoxia downregulates miR-93; (Y) overexpression of miR-93 (‘mimic’) decreases IRF9 mRNA expression and IRG1 (simulated IRG1 protein level is compared to IRG1 mRNA data) (Ganta et al., 2017). (A-Y) All values are for protein levels unless noted otherwise and are normalized (y-axes are relative expression). For normalization and display of results (simulation and data in A-Y): C, E, F, H, L, P, Q, W, X, mIRF9 and IRG1 in Y normalized to the respective t=0 (Ctr condition) values; U-V normalized to the respective values at the last experimental timepoints; G normalized to the values at the Ctr (hypoxia-treated) condition; I normalized to the values at 48 h of IFNγ treatment; N normalized to the values at 16 h of IFNγ treatment; R normalized to the values at 24 h of hypoxia; S normalized to the values at 12 h of hypoxia; T normalized to the values at 21% O_2; miR-93 in Y normalized to the values at 24 h of mimic transfection; all others normalized to their respective maximum values. S–simulation, D–experimental data, Hyp–hypoxia; Ctr–control/untreated condition unless otherwise noted.

Figure S5

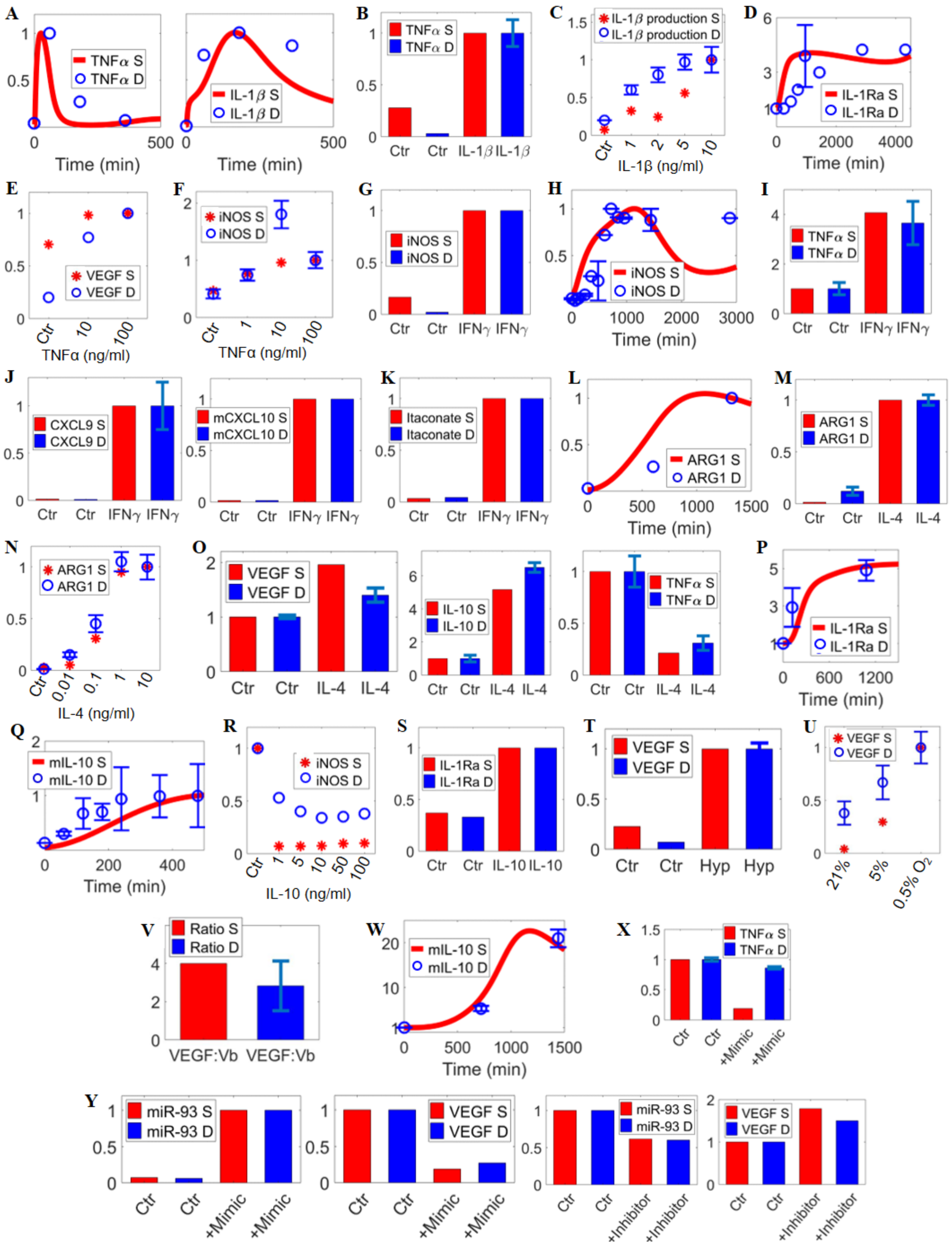

A
TNFα S
TNFα D
Time (min)
IL-1β S
IL-1β D
Time (min)
B
TNFα S
TNFα D
Ctr
Ctr
IL-1β
IL-1β
C
IL-1β production S
IL-1β production D
IL-1β (ng/ml)
D
IL-1Ra S
IL-1Ra D
Time (min)
E
VEGF S
VEGF D
TNFα (ng/ml)
F
iNOS S
iNOS D
TNFα (ng/ml)
G
iNOS S
iNOS D
IFNγ
H
iNOS S
iNOS D
Time (min)
I
TNFα S
TNFα D
J
CXCL9 S
CXCL9 D
mCXCL10 S
mCXCL10 D
K
Itaconate S
Itaconate D
L
ARG1 S
ARG1 D
Time (min)
M
ARG1 S
ARG1 D
IL-4
N
ARG1 S
ARG1 D
IL-4 (ng/ml)
O
VEGF S
VEGF D
IL-10 S
IL-10 D
TNFα S
TNFα D
P
IL-1Ra S
IL-1Ra D
Time (min)
Q
mIL-10 S
mIL-10 D
Time (min)
R
iNOS S
iNOS D
IL-10 (ng/ml)
S
IL-1Ra S
IL-1Ra D
IL-10
T
VEGF S
VEGF D
Hyp
U
VEGF S
VEGF D
21%
5%
0.5% O2
V
Ratio S
Ratio D
VEGF:Vb
W
mIL-10 S
mIL-10 D
Time (min)
X
TNFα S
TNFα D
+Mimic
Y
miR-93 S
miR-93 D
+Mimic
VEGF S
VEGF D
miR-93 S
miR-93 D
+Inhibitor
VEGF S
VEGF D
+Inhibitor

Figure S5. Additional quantitative model calibration of M1-M2 marker regulation (part 2); related to Figure 3. Model simulations and corresponding experimental data are shown together (references are listed in the order of the data mentioned). (A) IL-1β induces production of TNFα and IL-1β (simulated protein production rates are compared to the respective mRNA data) (Hu et al., 2005). (B) IL-1β induces TNFα secretion (Jayaraman et al., 2013), (C) IL-1β production in a dose-dependent manner (simulated IL-1β protein production rates are compared to data of IL-1β promoter activities) (Toda et al., 2002), and (D) secretion of IL-1Ra (Gabay et al., 1997; Kovalovsky et al., 1998). (E-F) TNFα dose-dependently induces VEGF secretion (Lu et al., 2012) and iNOS activity (simulated iNOS protein levels are compared to data of NO concentration) (Peiheng He, 2016). (G-H) IFNγ induces iNOS expression (Ohata et al., 1998; Vila-del Sol et al., 2007), (I) TNFα secretion (Davis et al., 2013), (J) CXCL9 secretion and CXCL10 mRNA expression (Mundra et al., 2016), and (K) itaconate production (Naujoks et al., 2016). (L-M) IL-4 induces ARG1 expression (Sheldon et al., 2013; Zanin et al., 2012) and (N) its dose-response relationship (Tachdjian et al., 2010); results in M and N compare simulated ARG1 protein levels to data of urea levels. (O) IL-4 induces VEGF secretion (Lim et al., 2017), IL-10 secretion, and downregulation of TNFα secretion (Zhang et al., 2011); combined IL-4 plus IL-13 treatments in data is simplified as IL-4 treatment only in simulations. (P) IL-4 induces IL-1Ra secretion over time (Liu et al., 1998; O'Connor et al., 2007). (Q) IL-10 induces IL-10 mRNA production (Staples et al., 2007), (R) inhibits iNOS production (Villalta et al., 2011), and (S) increases IL-1Ra secretion (Liu et al., 1998). (T-U) Hypoxia increases VEGF secretion (Mei et al., 2014) and its dose response (Eubank et al., 2011); (W) hypoxia increases IL-10 mRNA production (Meng et al., 2018). (V) Ratio of total VEGF to VEGF165b at control condition (Nowak et al., 2008). (X) Overexpression of miR-93 ('mimic') decreases hypoxia-induced TNFα secretion (Ganta et al., 2017). (Y) Overexpression of miR-93 decreases VEGF protein levels and inhibition of miR-93 ('inhibitor') increases VEGF protein levels (Lv et al., 2015). (A-Y) All values are for protein levels unless noted otherwise and are normalized (y-axes are relative expression, except for V as described above). For normalization and display of results (simulation and data in A-Y): D, I, O, P, R, W normalized to the respective t=0 (Ctr condition) values; C, E, F, N, U normalized to the values at the highest treatment concentration; L, Q normalized to the respective values at the last experimental timepoints; X normalized to the values at the Ctr (hypoxia-treated) condition; B normalized to the values at 24 h of IL-1β treatment; J normalized to the values at 24 h (for CXCL10) and 72 h (for CXCL9) of IFNγ treatment; K normalized to the values at 18 h of IFNγ treatment; M normalized to the values at 24 h of IL-4 treatment; S normalized to the values at 24 h of IL-10 treatment; T normalized to the values at 16 h of hypoxia; X normalized to the values at Ctr (hypoxia-treated) condition; miR-93 (mimic panel) in Y normalized to the values at 48 h of mimic transfection, the other 3 panels in Y normalized to the respective t=0 values; all others normalized to their respective maximum values. S–simulation, D–experimental data, Hyp–hypoxia; Ctr–control/untreated condition unless otherwise noted.

Figure S6

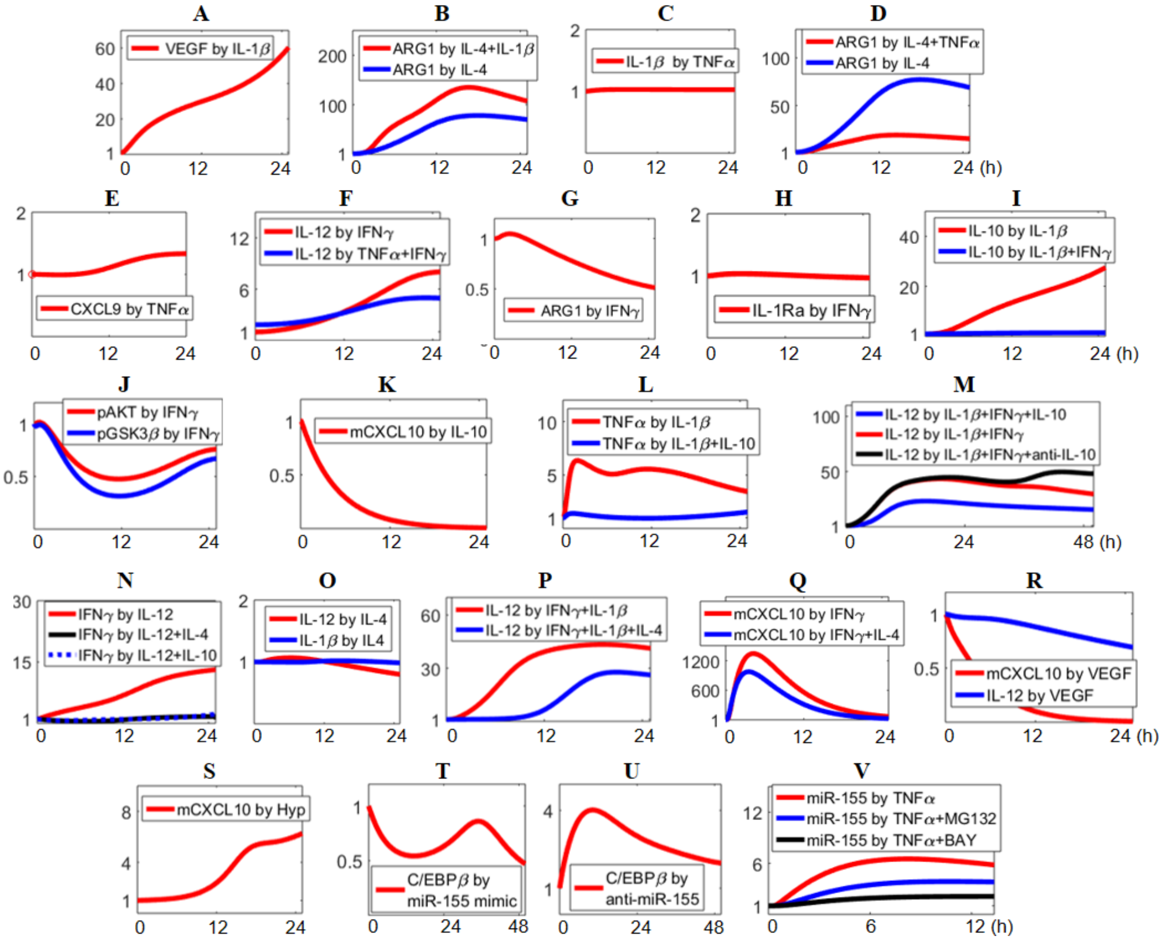

Figure S6. Constraining the model with additional qualitative experimental data; related to Figure 3. In addition to the quantitative datasets used in calibration, a number of qualitative macrophage data from literature was also incorporated into our model formulation and calibration. (A) IL-1β induces VEGF production (Lu et al., 2012). (B) IL-1β enhances IL-4-induced ARG1 expression (Sato et al., 2012). (C) TNFα does not significantly upregulate IL-1β secretion (Jayaraman et al., 2013; Lawlor et al., 2015). (D) TNFα inhibits IL-4-induced ARG1 expression. (Schleicher et al., 2016). (E) TNFα does not significantly upregulate CXCL9 secretion (Mundra et al., 2016). (F) TNFα can negatively regulate IL-12 secretion (Hodge-Dufour et al., 1998). (G) IFNγ inhibits ARG1 expression (Piccolo et al., 2017). (H) IFNγ does not significantly upregulate IL-1Ra (Liu et al., 1998). (I) IFNγ can negatively regulate IL-10 secretion (Hu et al., 2006). (J) IFNγ negatively regulates AKT activation and inhibitory phosphorylation of GSK3β (Hu et al., 2006). (K) IL-10 can downregulate CXCL10 expression (Cheeran et al., 2003). (L) IL-10 can negatively regulate TNFα secretion (Denys et al., 2002). (M) IL-10 can negatively regulate IL-12 secretion while inhibition of IL-10 signaling can promote IL-12 secretion (Rahim et al., 2005). (N) IL-

4 and IL-10 can decrease IFNγ secretion (Schindler et al., 2001). (O) IL-4 does not significantly upregulate secretion of IL-1β or IL-12 (Zhang et al., 2011). (P) IL-4 can inhibit IL-12 secretion (Bonder et al., 1999) and (Q) CXCL10 expression (Piccolo et al., 2017). (R) VEGF can reduce IL-12 secretion and CXCL10 expression (Wheeler et al., 2018). (S) Hypoxia increases CXCL10 expression (Danielsson et al., 2008). (T-U) Overexpression (mimic) of miR-155 reduces C/EBPβ expression and inhibition of miR-155 promotes C/EBPβ expression (Arranz et al., 2012). (V) Inhibition of NFκB signaling (through two types of inhibitors) can reduce miR-155 expression (Bala et al., 2011). (A-V) All results are normalized (y-axes, relative expression) to respective values at the t=0/control/untreated condition.

Figure S7

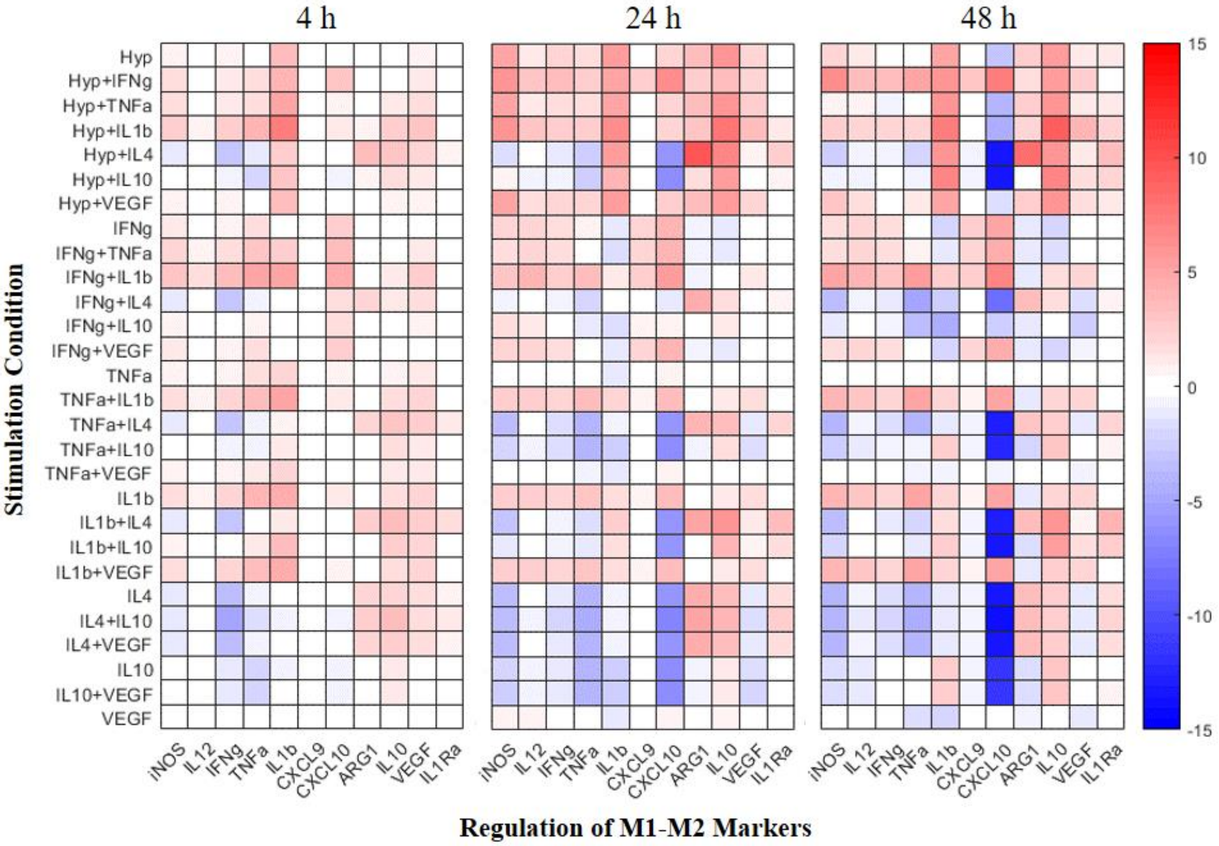

Figure S7. Macrophage polarization map under simulated *in vivo* stimulation conditions; related to Figure 5. A model-generated map of M1-M2 marker regulation (at 4, 24 and 48 h of stimulation) by macrophages under different stimulation conditions *in vivo* (7 cases of single stimulus, 21 cases of pairwise combined stimuli). For the 6 cytokines, their *in vivo* stimulation concentrations are assumed to be at 100 pg/ml; *in vivo* hypoxia is assumed to be 0.5% O2 (McKeown, 2014). Among the M1-M2 markers described, iNOS, ARG1, IL-12, IL-1Ra, CXCL9 are protein levels, CXCL10 is mRNA level, and the remaining ones are the respective protein production rates calculated by the model. All results are normalized to the untreated/t=0 values and then log2 transformed. Hyp–hypoxia.

Figure S8

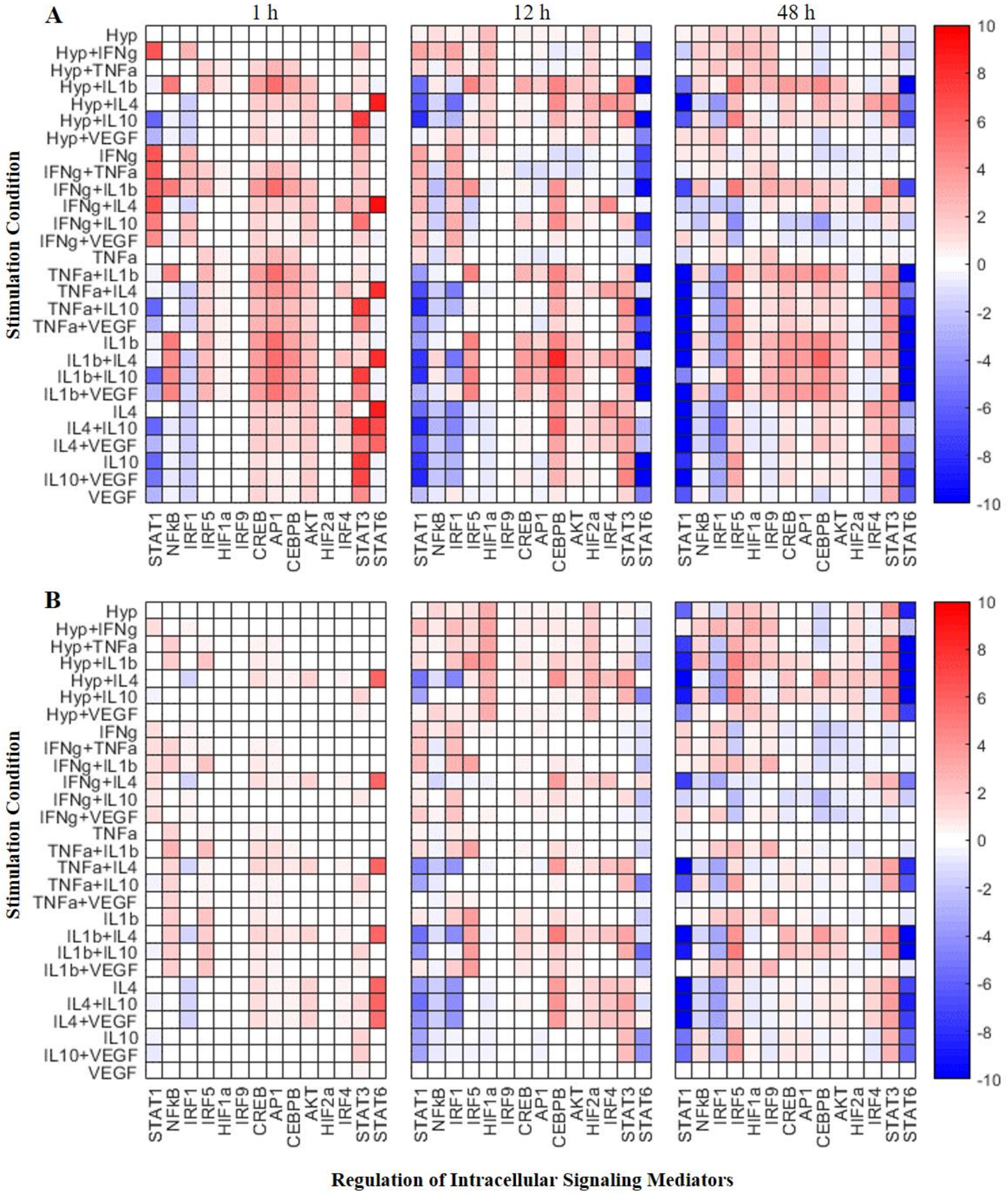

Figure S8. Macrophage transcriptional regulation map under simulated (A) *in vitro* and (B) *in vivo* stimulation conditions; related to Figure 5. A model-generated map of macrophage transcriptional regulation (at 1, 12 and 48 h of stimulation) under different stimulation conditions *in vitro and in vivo* (7

cases of single stimulus, 21 cases of pairwise combined stimuli, all conditions are the same as in Figs.5A and S7). For all the transcriptional mediators described, STAT1/3/6 refer to the respective activated dimers in nucleus, HIF1/2α refer to the respective HIFα/HIF1β heterodimers in nucleus, NFκB refer to free NFκB molecules in nucleus, CREB and AKT refer to respective phosphorylated forms, all others refer to respective protein expression levels in cell. All results are normalized to the untreated/t=0 values and then log2 transformed. Hyp–hypoxia.

Figure S9

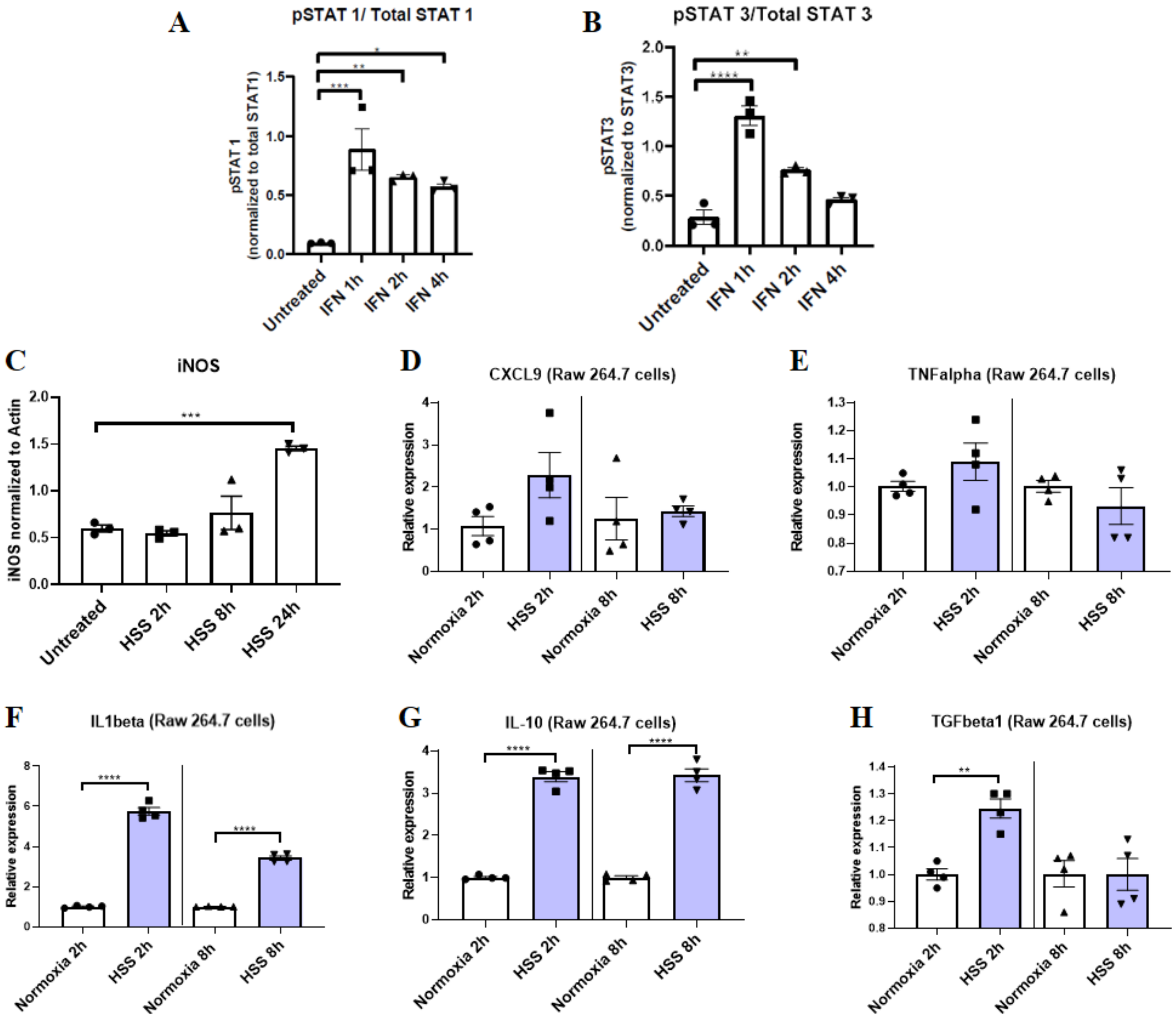

Figure S9. Experimental analysis of STAT signaling and HSS-induced M1-M2 marker regulation; related to Figures 2 and 6. Data shown here were used in model calibration (in Figs.2, S4) and analysis (in Fig.6). **(**A-B) Western Blotting analysis of pSTAT1 and pSTAT3 expression normalized to STAT1 and STAT3 respectively in macrophages treated with interferon gamma (IFN) for the times indicated. (C) Western Blotting analysis of iNOS expression normalized to actin in macrophages exposed to hypoxia serum starvation (HSS) for the times indicated. (D, F, G) qPCR analysis of CXCL9, IL-1β and IL-10 expression normalized to Rplp0 in macrophages under normoxia or HSS for the times indicated. (E and H) qPCR analysis of TNFα and TGFβ1 normalized to GAPDH in macrophages under normoxia or HSS for the times indicated. (A-H) Mean values, standard deviations and individual datapoints are displayed (A-C: n=3; D-H: n=4; * $p<0.05$; ** $p<0.01$; *** $p<0.001$; **** $p<0.0001$).

Figure S10

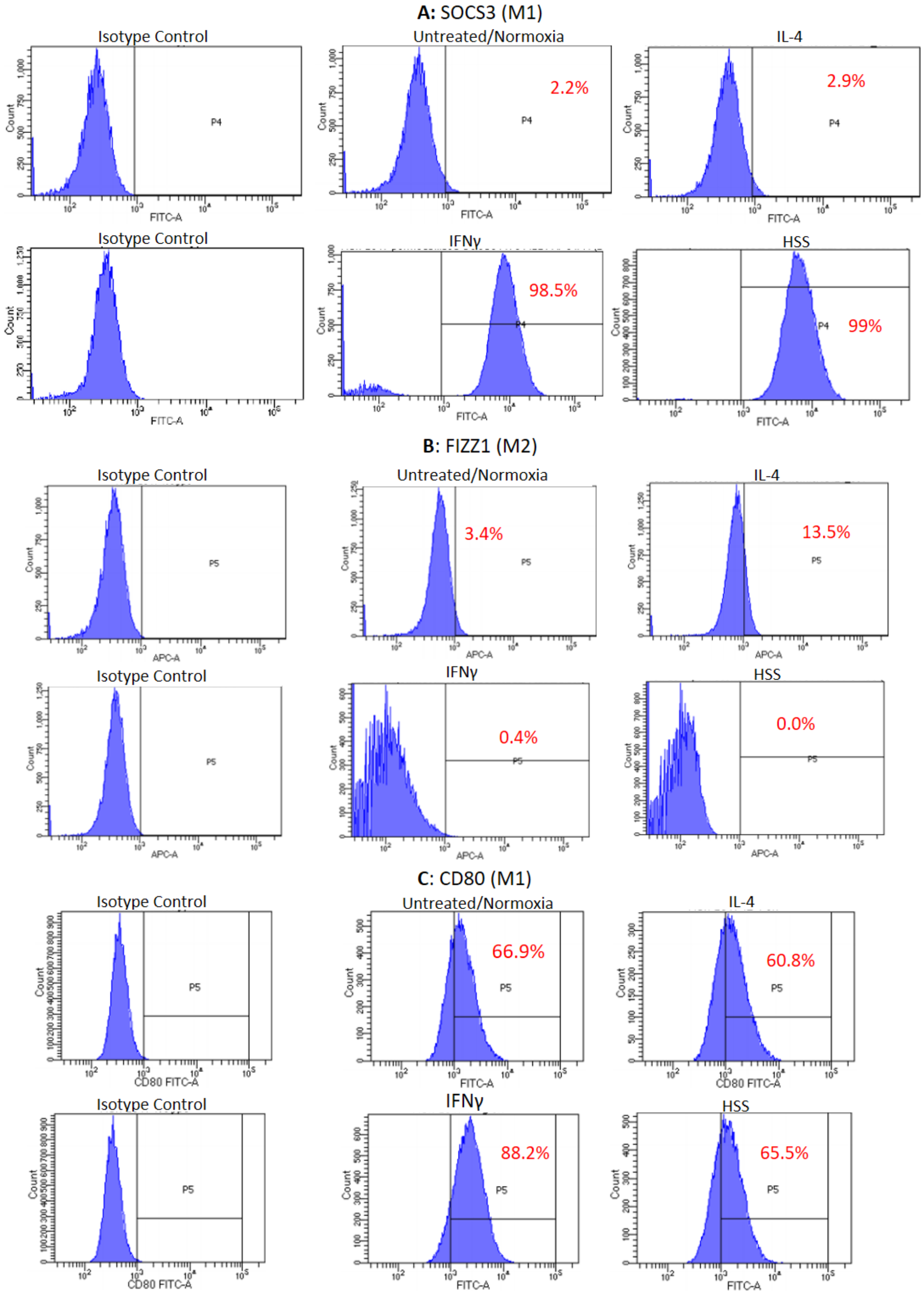

Figure S10. Flow cytometry analysis of macrophage markers; related to Figure 6. Data shown here were used in model analysis (in Fig.6). (A-C) Percentages of parent events sorting SOCS3, FIZZ1 or CD80 as M1-M2 markers in macrophages under normoxia, treated with 10 ng/ml of IL-4, treated with 50 ng/ml of IFNγ, and under hypoxia serum starvation (HSS) for 6 hours.

Figure S11

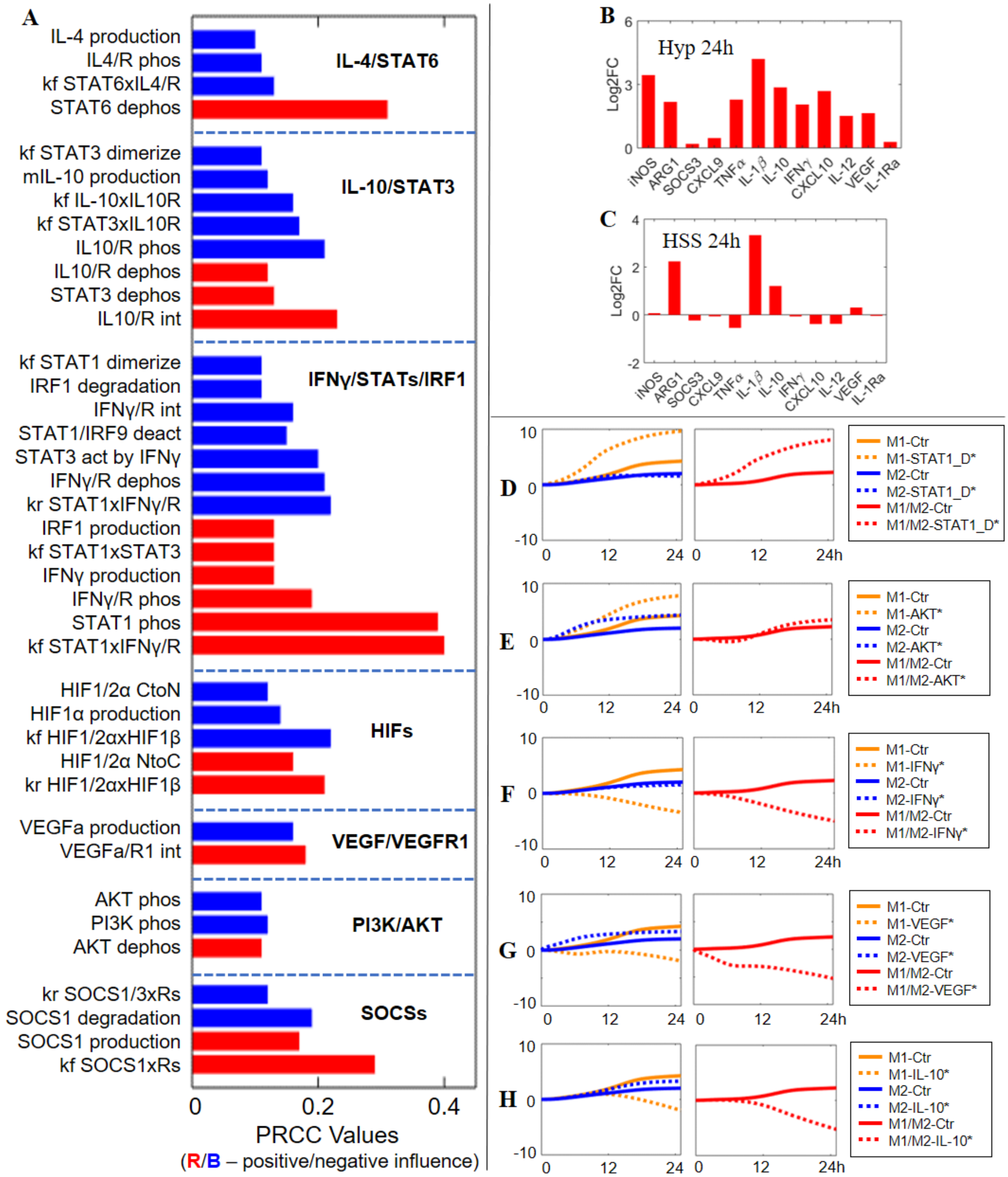

Figure S11. Macrophage response under HSS and *in silico* targeted interventions to promote M2-like phenotypes; related to Figure 6. (A) Sensitivity indices (red – positive, blue – negative) of the most influential model parameters (e.g. absolute PRCC values greater than 0.1) that control M1 and M2 marker expression in terms of the 24h time-course integral of M1/M2 score in simulated HSS* (represented in

model as 2% O_2 plus global reduction in protein production rates). The identified parameters are categorized into 7 modules and their functions are briefly described. (B) Model-derived profile of macrophage M1-M2 marker expression at 24 h under HSS (simulated as hypoxia only). (C) Model-derived profile of macrophage M1-M2 marker expression at 24 h under simulated HSS*. (B-C) Expression levels are normalized to the t=0 (control condition) values and then log2 transformed. (D-H) Simulated M1 (orange), M2 (blue) profiles (e.g. M1 and M2 scores) and overall M1/M2 scores (red) over time under hypoxia (2% O_2) with various targeted interventions (label as 'species*') proposed by the sensitivity analysis. Results are normalized to the respective values at t=0 and then log10 transformed (y-axes). Implementation of targeted interventions: (D) inhibition of STAT1 dimerization – 0.01x kf5; (E) increased AKT activation – 10x k138; (F) Inhibition of IFNγ production – 0.1x k73; (G) Increased VEGF (pro-angiogenic form) production – 10x k72; (H) Increased IL-10 production – 10x k44. (A) More details about the listed parameters are described in Table S1 (parameter labels from top to bottom are k104, kf102, kf24, k26; kf125, k44, kf144, kf146, kf145, kr145, k127, k148; kf5, k85, k12, k117, k134, kr2, kr3, k52, kf131, k73, kf2, k4, kf3; kf58, k54, kf60, kr58, kr60; k72, k201; k138, k136, k140; kr11, k101, k135, kf11).

Figure S12

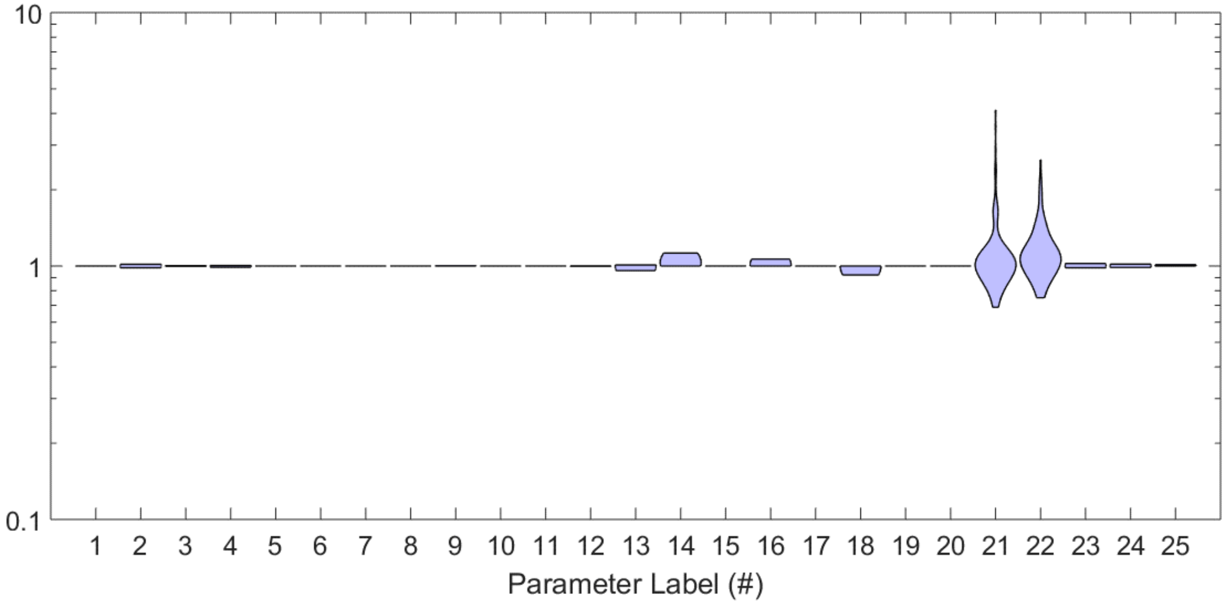

Figure S12. Parameter estimate distributions after bootstrapping; related to Figure 6. Parameter estimate distributions (represented by violin plots) of the top 25 most influential parameters. All parameter values were allowed to vary from 0.1x to 10x during bootstrapping and are here normalized to their respective reference values for display (y-axis in log scale). The parameters labeled #1-25 are kf3, kr2, k4, kr3, kf2, kf146, k117, kf11, k148, k12, k134, kf131, k52, k201, kf207, k209, k202, k203, k127, k135, k150, k82, kf145, k73, kf73 (see Table S1 for more details of these parameters).

Figure S13

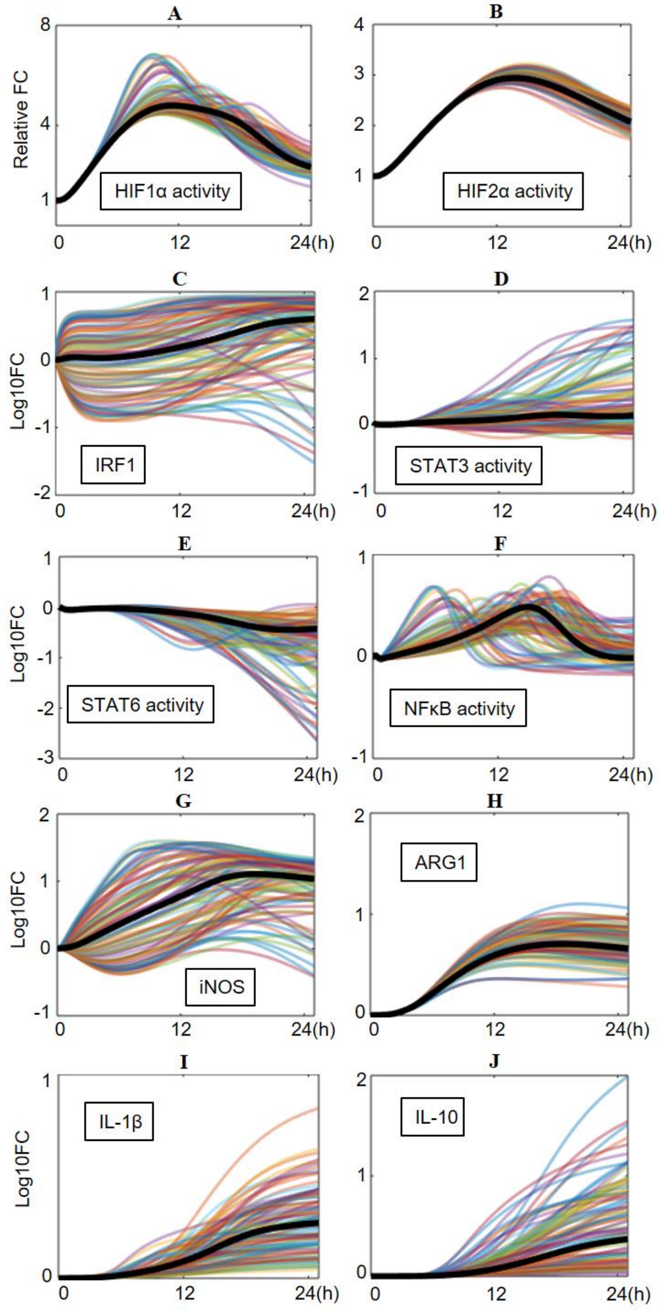

Figure S13. In silico analysis of hypoxia-driven transcriptional and marker regulation at the single-cell level; related to Figure 7. Simulated time-course responses (under hypoxia, 2% O_2) of 100 model-generated virtual macrophages in terms of their transcriptional activities of (A) HIF1α, (B) HIF2α, (C) IRF1, (D) STAT3, (E) STAT6, (F) NFκB, and M1-M2 marker regulation of (G-H) cellular iNOS and ARG1 expression, and (I-J) IL-1β and IL-10 secretion. (A-J) Results are normalized to the respective values at t=0 for A-B and then log10 transformed for C-J (y-axes). Black bolded lines are the trajectories of the reference model.

Figure S14

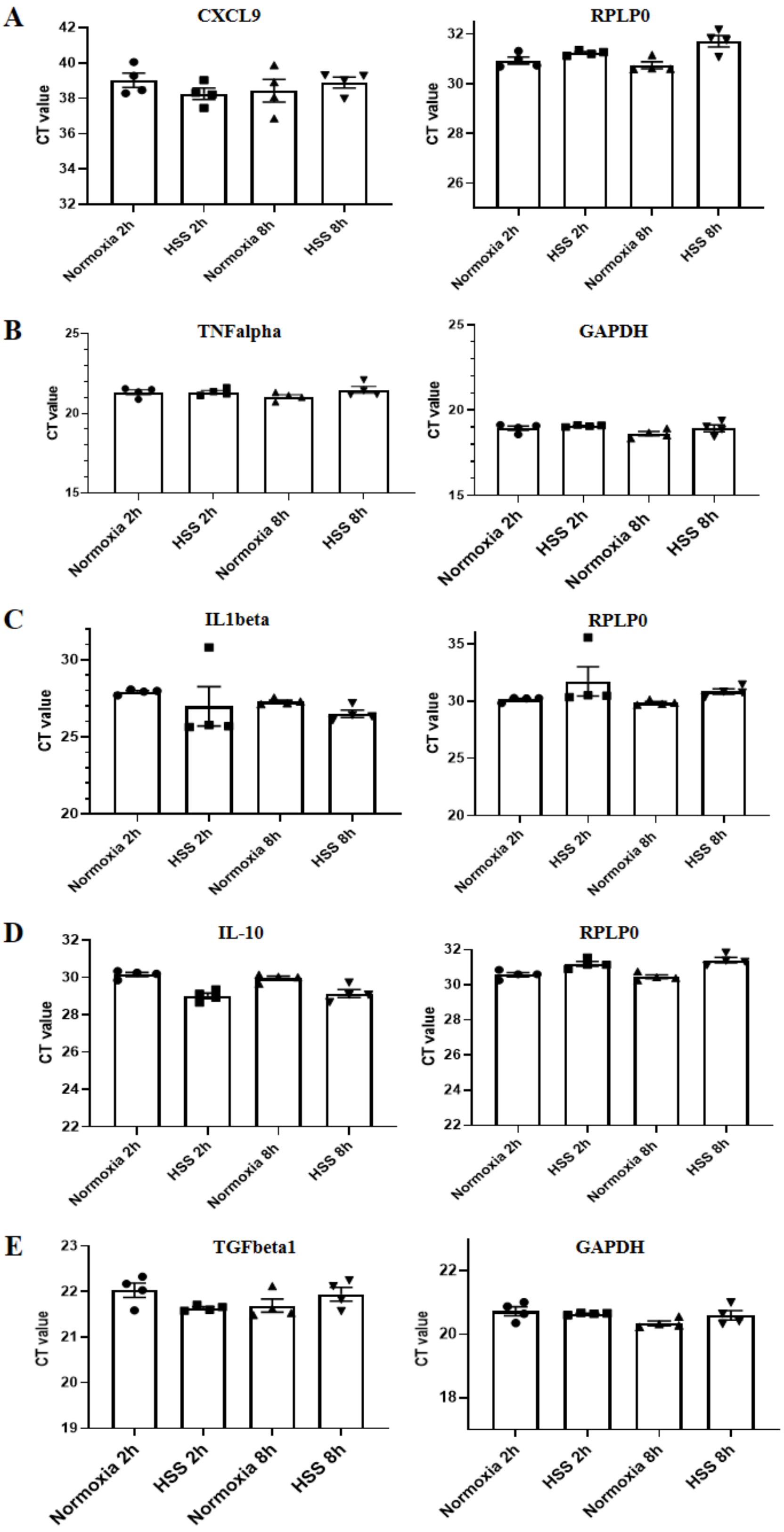
A
CXCL9
RPLP0
B
TNFalpha
GAPDH
C
IL1beta
RPLP0
D
IL-10
RPLP0
E
TGFbeta1
GAPDH
CT value
Normoxia 2h
HSS 2h
Normoxia 8h
HSS 8h

Figure S14. Raw data for the qPCR analysis presented in Fig.S9; related to Figure 6. (A, C, D) qPCR CT values under normoxia and hypoxia serum starvation (HSS), for the times indicated, for CXCL9, IL-1β, IL-10 and Rplp0. (B, E) qPCR CT values under normoxia and HSS, for the times indicated, for TNFα, TGFβ1 and GAPDH. (A-E) Mean levels, standard deviations and individual datapoints are displayed (n=4).

Figure S15

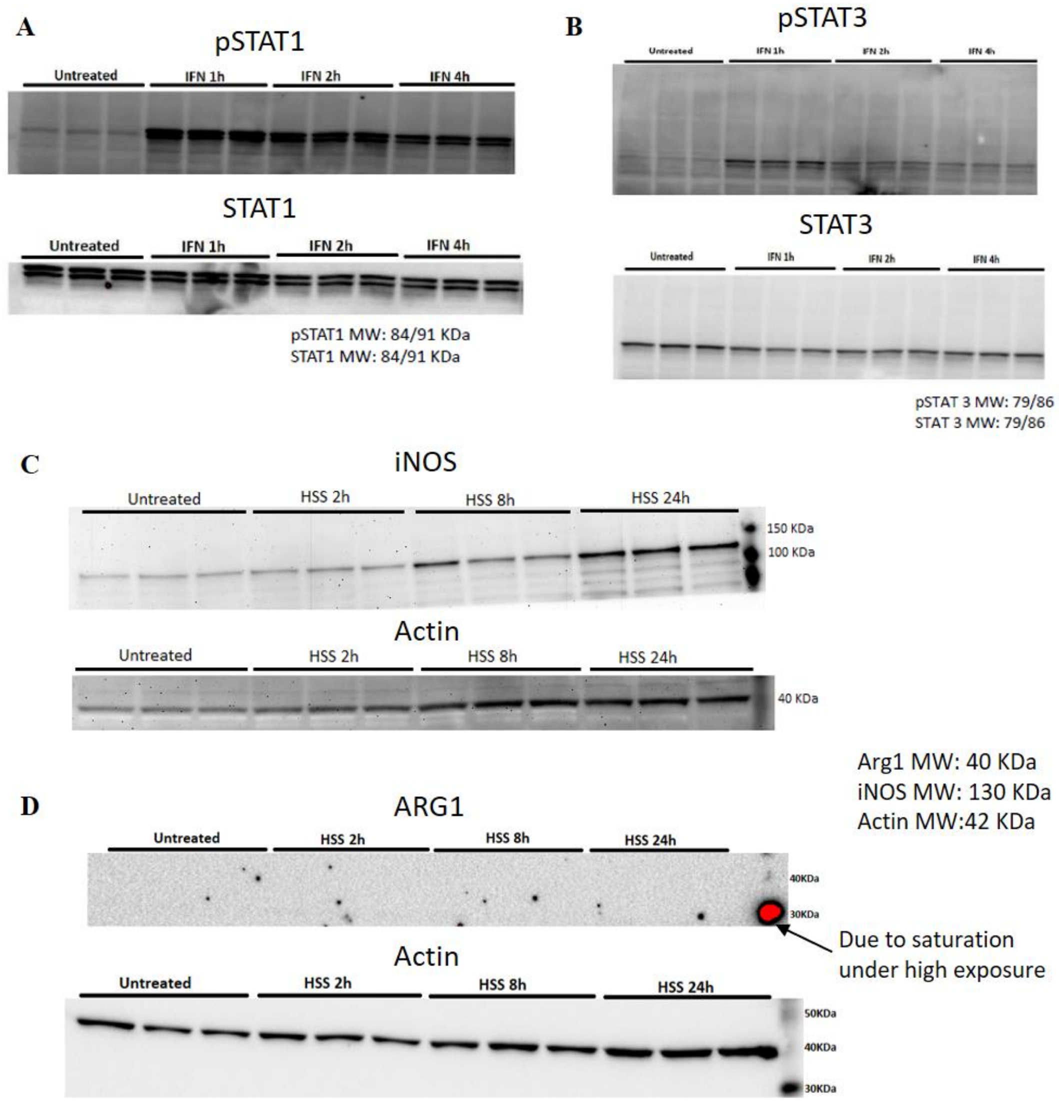

Figure S15. Raw data for the Western Blot analysis presented in Fig.S9; related to Figures 2 and 6. (A-B) Western Blots (n=3) of pSTAT1/pSTAT3 and total STAT1/STAT3 expression in macrophages treated with interferon gamma (IFN) for the times indicated. (C-D) Western Blots (n=3) of iNOS, ARG1 (below detection limit) and Actin expression in macrophages exposed to hypoxia serum starvation (HSS) for the times indicated.

Figure S16

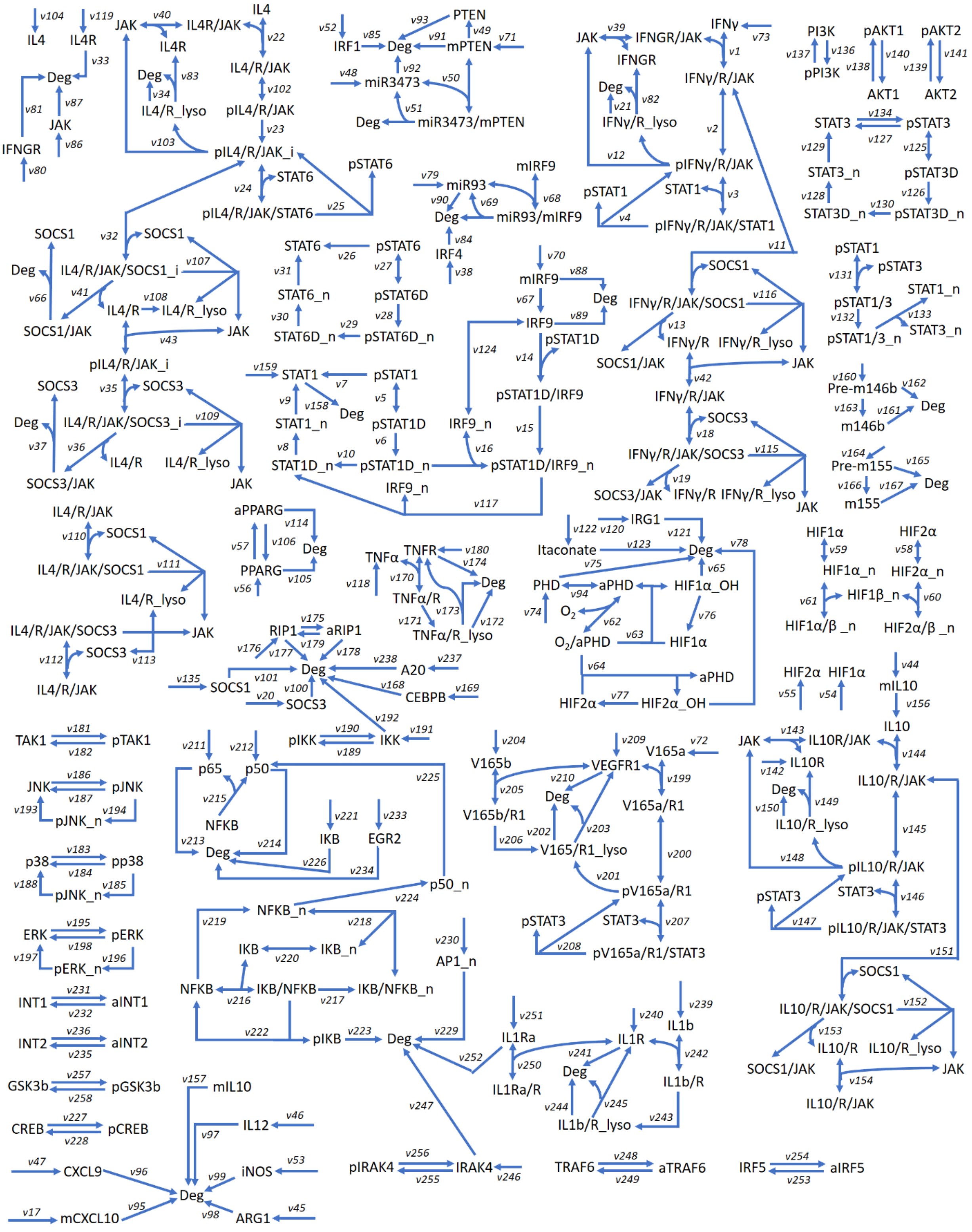
IL4
IL4R
JAK
IL4R/JAK
IL4R
Deg
IL4/R/JAK
IL4/R_lyso
pIL4/R/JAK
pIL4/R/JAK_i
STAT6
pSTAT6
pIL4/R/JAK/STAT6
IFNGR
IRF1
PTEN
mPTEN
miR3473
miR3473/mPTEN
miR93
mIRF9
miR93/mIRF9
IFNγ
IFNGR/JAK
IFNγ/R/JAK
IFNγ/R_lyso
pIFNγ/R/JAK
pSTAT1
STAT1
pIFNγ/R/JAK/STAT1
PI3K
pPI3K
pAKT1
AKT1
pAKT2
AKT2
STAT3
pSTAT3
STAT3_n
pSTAT3D
STAT3D_n
pSTAT3D_n
SOCS1
IL4/R/JAK/SOCS1_i
IL4/R
SOCS1/JAK
STAT6_n
pSTAT6D
STAT6D_n
pSTAT6D_n
IRF4
mIRF9
IRF9
pSTAT1D
IFNγ/R/JAK/SOCS1
IFNγ/R
IFNγ/R_lyso
pSTAT1/3
STAT1_n
pSTAT1/3_n
STAT3_n
SOCS3
IL4/R/JAK/SOCS3_i
SOCS3/JAK
STAT1_n
STAT1D_n
pSTAT1D_n
IRF9_n
pSTAT1D/IRF9
pSTAT1D/IRF9_n
IFNγ/R/JAK/SOCS3
Pre-m146b
m146b
Pre-m155
m155
aPPARG
PPARG
TNFα
TNFR
TNFα/R
TNFα/R_lyso
IRG1
Itaconate
PHD
aPHD
O_2
O_2/aPHD
HIF1α_OH
HIF1α
HIF2α
HIF2α_OH
HIF1α_n
HIF2α_n
HIF1β_n
HIF1α/β _n
HIF2α/β _n
IL4/R/JAK/SOCS1
IL4/R/JAK/SOCS3
RIP1
aRIP1
A20
CEBPB
mIL10
IL10
TAK1
pTAK1
JNK
pJNK
pJNK_n
p38
pp38
ERK
pERK
pERK_n
INT1
aINT1
INT2
aINT2
GSK3b
pGSK3b
CREB
pCREB
p65
p50
NFKB
IKB
EGR2
pIKK
IKK
p50_n
NFKB_n
IKB_n
IKB/NFKB
IKB/NFKB_n
AP1_n
pIKB
V165b
VEGFR1
V165a
V165b/R1
V165a/R1
V165/R1_lyso
pV165a/R1
pV165a/R1/STAT3
IL10R/JAK
IL10R
IL10/R/JAK
IL10/R_lyso
pIL10/R/JAK
pIL10/R/JAK/STAT3
IL10/R/JAK/SOCS1
IL10/R
IL10/R/JAK
IL1Ra
IL1R
IL1b
IL1Ra/R
IL1b/R
IL1b/R_lyso
IL12
CXCL9
mCXCL10
iNOS
ARG1
pIRAK4
IRAK4
TRAF6
aTRAF6
IRF5
aIRF5

Figure S16. Detailed model diagram; related to Figure 1. A more detailed model diagram with all model species and reaction fluxes (labeled). Note that not all regulatory mechanisms were depicted here due to the size limit of the figure (e.g. transcriptional regulations were not explicitly drawn here); please refer to Table S1 for a full mechanistic description of the model.

References for Supplemental Information

ARRANZ, A., DOXAKI, C., VERGADI, E., MARTINEZ DE LA TORRE, Y., VAPORIDI, K., LAGOUDAKI, E. D., IERONYMAKI, E., ANDROULIDAKI, A., VENIHAKI, M., MARGIORIS, A. N., STATHOPOULOS, E. N., TSICHLIS, P. N. & TSATSANIS, C. 2012. Akt1 and Akt2 protein kinases differentially contribute to macrophage polarization. *Proc Natl Acad Sci U S A,* 109**,** 9517-22.

BALA, S., MARCOS, M., KODYS, K., CSAK, T., CATALANO, D., MANDREKAR, P. & SZABO, G. 2011. Up-regulation of microRNA-155 in macrophages contributes to increased tumor necrosis factor {alpha} (TNF{alpha}) production via increased mRNA half-life in alcoholic liver disease. *J Biol Chem,* 286**,** 1436-44.

BERRA, E., BENIZRI, E., GINOUVES, A., VOLMAT, V., ROUX, D. & POUYSSEGUR, J. 2003. HIF prolyl-hydroxylase 2 is the key oxygen sensor setting low steady-state levels of HIF-1alpha in normoxia. *EMBO J,* 22**,** 4082-90.

BLOUIN, C. M. & LAMAZE, C. 2013. Interferon gamma receptor: the beginning of the journey. *Front Immunol,* 4**,** 267.

BONDER, C. S., FINLAY-JONES, J. J. & HART, P. H. 1999. Interleukin-4 regulation of human monocyte and macrophage interleukin-10 and interleukin-12 production. Role of a functional interleukin-2 receptor gamma-chain. *Immunology,* 96**,** 529-36.

BOUCHER, J. M., CLARK, R. P., CHONG, D. C., CITRIN, K. M., WYLIE, L. A. & BAUTCH, V. L. 2017. Dynamic alterations in decoy VEGF receptor-1 stability regulate angiogenesis. *Nat Commun,* 8**,** 15699.

CARTA, L., PASTORINO, S., MELILLO, G., BOSCO, M. C., MASSAZZA, S. & VARESIO, L. 2001. Engineering of macrophages to produce IFN-gamma in response to hypoxia. *J Immunol,* 166**,** 5374-80.

CASALETTO, J. B. & MCCLATCHEY, A. I. 2012. Spatial regulation of receptor tyrosine kinases in development and cancer. *Nat Rev Cancer,* 12**,** 387-400.

CELADA, A. & SCHREIBER, R. D. 1987. Internalization and degradation of receptor-bound interferon-gamma by murine macrophages. Demonstration of receptor recycling. *J Immunol,* 139**,** 147-53.

CENDROWSKI, J., MAMINSKA, A. & MIACZYNSKA, M. 2016. Endocytic regulation of cytokine receptor signaling. *Cytokine Growth Factor Rev,* 32**,** 63-73.

CHEERAN, M. C., HU, S., SHENG, W. S., PETERSON, P. K. & LOKENSGARD, J. R. 2003. CXCL10 production from cytomegalovirus-stimulated microglia is regulated by both human and viral interleukin-10. *J Virol,* 77**,** 4502-15.

CHEUNG, P. C., CAMPBELL, D. G., NEBREDA, A. R. & COHEN, P. 2003. Feedback control of the protein kinase TAK1 by SAPK2a/p38alpha. *EMBO J,* 22**,** 5793-805.

CHOI, Y. S., PARK, J. K., KANG, E. H., LEE, Y. K., KIM, T. K., CHUNG, J. H., ZIMMERER, J. M., CARSON, W. E., SONG, Y. W. & LEE, Y. J. 2013. Cytokine signaling-1 suppressor is inducible by IL-1beta and inhibits the catabolic effects of IL-1beta in chondrocytes: its implication in the paradoxical joint-protective role of IL-1beta. *Arthritis Res Ther,* 15**,** R191.

COVARRUBIAS, A. J., AKSOYLAR, H. I., YU, J., SNYDER, N. W., WORTH, A. J., IYER, S. S., WANG, J., BEN-SAHRA, I., BYLES, V., POLYNNE-STAPORNKUL, T., ESPINOSA, E. C., LAMMING, D., MANNING, B. D., ZHANG, Y., BLAIR, I. A. & HORNG, T. 2016. Akt-mTORC1 signaling regulates Acly to integrate metabolic input to control of macrophage activation. *Elife,* 5.

CROKER, B. A., KIU, H. & NICHOLSON, S. E. 2008. SOCS regulation of the JAK/STAT signalling pathway. *Semin Cell Dev Biol,* 19**,** 414-22.

CUMMINS, E. P., BERRA, E., COMERFORD, K. M., GINOUVES, A., FITZGERALD, K. T., SEEBALLUCK, F., GODSON, C., NIELSEN, J. E., MOYNAGH, P., POUYSSEGUR, J. & TAYLOR, C. T. 2006. Prolyl hydroxylase-1 negatively regulates IkappaB kinase-beta, giving insight into hypoxia-induced NFkappaB activity. *Proc Natl Acad Sci U S A,* 103**,** 18154-9.

CURTALE, G., MIROLO, M., RENZI, T. A., ROSSATO, M., BAZZONI, F. & LOCATI, M. 2013. Negative regulation of Toll-like receptor 4 signaling by IL-10-dependent microRNA-146b. *Proc Natl Acad Sci U S A,* 110**,** 11499-504.

DANIELSSON, K. N., RYDBERG, E. K., INGELSTEN, M., AKYUREK, L. M., JIRHOLT, P., ULLSTROM, C., FORSBERG, G. B., BOREN, J., WIKLUND, O. & HULTEN, L. M. 2008. 15-Lipoxygenase-2 expression in human macrophages induces chemokine secretion and T cell migration. *Atherosclerosis,* 199**,** 34-40.

DATE, D., DAS, R., NARLA, G., SIMON, D. I., JAIN, M. K. & MAHABELESHWAR, G. H. 2014. Kruppel-like transcription factor 6 regulates inflammatory macrophage polarization. *J Biol Chem,* 289**,** 10318-29.

DAVIS, M. J., TSANG, T. M., QIU, Y., DAYRIT, J. K., FREIJ, J. B., HUFFNAGLE, G. B. & OLSZEWSKI, M. A. 2013. Macrophage M1/M2 polarization dynamically adapts to changes in cytokine microenvironments in Cryptococcus neoformans infection. *mBio,* 4**,** e00264-13.

DELGOFFE, G. M. & VIGNALI, D. A. 2013. STAT heterodimers in immunity: A mixed message or a unique signal? *JAKSTAT,* 2**,** e23060.

DENYS, A., UDALOVA, I. A., SMITH, C., WILLIAMS, L. M., CIESIELSKI, C. J., CAMPBELL, J., ANDREWS, C., KWAITKOWSKI, D. & FOXWELL, B. M. 2002. Evidence for a dual mechanism for IL-10 suppression of TNF-alpha production that does not involve inhibition of p38 mitogen-activated protein kinase or NF-kappa B in primary human macrophages. *J Immunol,* 168**,** 4837-45.

DICKENSHEETS, H., VAZQUEZ, N., SHEIKH, F., GINGRAS, S., MURRAY, P. J., RYAN, J. J. & DONNELLY, R. P. 2007. Suppressor of cytokine signaling-1 is an IL-4-inducible gene in macrophages and feedback inhibits IL-4 signaling. *Genes Immun,* 8**,** 21-7.

DRIPPS, D. J., BRANDHUBER, B. J., THOMPSON, R. C. & EISENBERG, S. P. 1991. Interleukin-1 (IL-1) receptor antagonist binds to the 80-kDa IL-1 receptor but does not initiate IL-1 signal transduction. *J Biol Chem,* 266**,** 10331-6.

DZAMKO, N., INESTA-VAQUERA, F., ZHANG, J., XIE, C., CAI, H., ARTHUR, S., TAN, L., CHOI, H., GRAY, N., COHEN, P., PEDRIOLI, P., CLARK, K. & ALESSI, D. R. 2012. The IkappaB kinase family phosphorylates the Parkinson's disease kinase LRRK2 at Ser935 and Ser910 during Toll-like receptor signaling. *PLoS One,* 7**,** e39132.

ENDO, Y., BLINOVA, K., ROMANTSEVA, T., GOLDING, H. & ZAITSEVA, M. 2014. Differences in PGE2 production between primary human monocytes and differentiated macrophages: role of IL-1beta and TRIF/IRF3. *PLoS One,* 9**,** e98517.

ERMOLAEVA, M. A., MICHALLET, M. C., PAPADOPOULOU, N., UTERMOHLEN, O., KRANIDIOTI, K., KOLLIAS, G., TSCHOPP, J. & PASPARAKIS, M. 2008. Function of TRADD in tumor necrosis factor receptor 1 signaling and in TRIF-dependent inflammatory responses. *Nat Immunol,* 9**,** 1037-46.

ETEMADI, N., CHOPIN, M., ANDERTON, H., TANZER, M. C., RICKARD, J. A., ABEYSEKERA, W., HALL, C., SPALL, S. K., WANG, B., XIONG, Y., HLA, T., PITSON, S. M., BONDER, C. S., WONG, W. W., ERNST, M., SMYTH, G. K., VAUX, D. L., NUTT, S. L., NACHBUR, U. & SILKE, J. 2015. TRAF2 regulates TNF and NF-kappaB signalling to suppress apoptosis and skin inflammation independently of Sphingosine kinase 1. *Elife,* 4.

EUBANK, T. D., RODA, J. M., LIU, H., O'NEIL, T. & MARSH, C. B. 2011. Opposing roles for HIF-1alpha and HIF-2alpha in the regulation of angiogenesis by mononuclear phagocytes. *Blood,* 117**,** 323-32.

FELIERS, D., CHEN, X., AKIS, N., CHOUDHURY, G. G., MADAIO, M. & KASINATH, B. S. 2005. VEGF regulation of endothelial nitric oxide synthase in glomerular endothelial cells. *Kidney Int,* 68**,** 1648-59.

FISCHER, R., MAIER, O., NAUMER, M., KRIPPNER-HEIDENREICH, A., SCHEURICH, P. & PFIZENMAIER, K. 2011. Ligand-induced internalization of TNF receptor 2 mediated by a di-leucin motif is dispensable for activation of the NFkappaB pathway. *Cell Signal,* 23**,** 161-70.

FUNAKOSHI-TAGO, M., KAMADA, N., SHIMIZU, T., HASHIGUCHI, Y., TAGO, K., SONODA, Y. & KASAHARA, T. 2009. TRAF6 negatively regulates TNFalpha-induced NF-kappaB activation. *Cytokine,* 45**,** 72-9.

GABAY, C., SMITH, M. F., EIDLEN, D. & AREND, W. P. 1997. Interleukin 1 receptor antagonist (IL-1Ra) is an acute-phase protein. *J Clin Invest,* 99**,** 2930-40.

GANTA, V. C., CHOI, M. H., KUTATELADZE, A., FOX, T. E., FARBER, C. R. & ANNEX, B. H. 2017. A MicroRNA93-Interferon Regulatory Factor-9-Immunoresponsive Gene-1-Itaconic Acid Pathway Modulates M2-Like Macrophage Polarization to Revascularize Ischemic Muscle. *Circulation,* 135**,** 2403-2425.

GENG, J., ITO, Y., SHI, L., AMIN, P., CHU, J., OUCHIDA, A. T., MOOKHTIAR, A. K., ZHAO, H., XU, D., SHAN, B., NAJAFOV, A., GAO, G., AKIRA, S. & YUAN, J. 2017. Regulation of RIPK1 activation by TAK1-mediated phosphorylation dictates apoptosis and necroptosis. *Nat Commun,* 8**,** 359.

GOTSCHEL, F., KERN, C., LANG, S., SPARNA, T., MARKMANN, C., SCHWAGER, J., MCNELLY, S., VON WEIZSACKER, F., LAUFER, S., HECHT, A. & MERFORT, I. 2008. Inhibition of GSK3 differentially modulates NF-kappaB, CREB, AP-1 and beta-catenin signaling in hepatocytes, but fails to promote TNF-alpha-induced apoptosis. *Exp Cell Res,* 314**,** 1351-66.

GREEN, D. S., YOUNG, H. A. & VALENCIA, J. C. 2017. Current prospects of type II interferon gamma signaling and autoimmunity. *J Biol Chem,* 292**,** 13925-13933.

GULEN, M. F., BULEK, K., XIAO, H., YU, M., GAO, J., SUN, L., BEUREL, E., KAIDANOVICH-BEILIN, O., FOX, P. L., DICORLETO, P. E., WANG, J. A., QIN, J., WALD, D. N., WOODGETT, J. R., JOPE, R. S., CARMAN, J., DONGRE, A. & LI, X. 2012. Inactivation of the enzyme GSK3alpha by the kinase IKKi promotes AKT-mTOR signaling pathway that mediates interleukin-1-induced Th17 cell maintenance. *Immunity,* 37**,** 800-12.

HANSEN, B., DITTRICH-BREIHOLZ, O., KRACHT, M. & WINDHEIM, M. 2013. Regulation of NF-kappaB-dependent gene expression by ligand-induced endocytosis of the interleukin-1 receptor. *Cell Signal,* 25**,** 214-28.

HE, X., TANG, R., SUN, Y., WANG, Y. G., ZHEN, K. Y., ZHANG, D. M. & PAN, W. Q. 2016. MicroR-146 blocks the activation of M1 macrophage by targeting signal transducer and activator of transcription 1 in hepatic schistosomiasis. *EBioMedicine,* 13**,** 339-347.

HO, H. H. & IVASHKIV, L. B. 2006. Role of STAT3 in type I interferon responses. Negative regulation of STAT1-dependent inflammatory gene activation. *J Biol Chem,* 281**,** 14111-8.

HODGE-DUFOUR, J., MARINO, M. W., HORTON, M. R., JUNGBLUTH, A., BURDICK, M. D., STRIETER, R. M., NOBLE, P. W., HUNTER, C. A. & PURE, E. 1998. Inhibition of interferon gamma induced interleukin 12 production: a potential mechanism for the anti-inflammatory activities of tumor necrosis factor. *Proc Natl Acad Sci U S A,* 95**,** 13806-11.

HSU, A. T., LUPANCU, T. J., LEE, M. C., FLEETWOOD, A. J., COOK, A. D., HAMILTON, J. A. & ACHUTHAN, A. 2018. Epigenetic and transcriptional regulation of IL4-induced CCL17 production in human monocytes and murine macrophages. *J Biol Chem,* 293**,** 11415-11423.

HU, X., HO, H. H., LOU, O., HIDAKA, C. & IVASHKIV, L. B. 2005. Homeostatic role of interferons conferred by inhibition of IL-1-mediated inflammation and tissue destruction. *J Immunol,* 175**,** 131-8.

HU, X., PAIK, P. K., CHEN, J., YARILINA, A., KOCKERITZ, L., LU, T. T., WOODGETT, J. R. & IVASHKIV, L. B. 2006. IFN-gamma suppresses IL-10 production and synergizes with TLR2 by regulating GSK3 and CREB/AP-1 proteins. *Immunity,* 24**,** 563-74.

IMAMURA, K., SPRIGGS, D. & KUFE, D. 1987. Expression of tumor necrosis factor receptors on human monocytes and internalization of receptor bound ligand. *J Immunol,* 139**,** 2989-92.

IMTIYAZ, H. Z., WILLIAMS, E. P., HICKEY, M. M., PATEL, S. A., DURHAM, A. C., YUAN, L. J., HAMMOND, R., GIMOTTY, P. A., KEITH, B. & SIMON, M. C. 2010. Hypoxia-inducible factor 2alpha regulates macrophage function in mouse models of acute and tumor inflammation. *J Clin Invest,* 120**,** 2699-714.

JAYARAMAN, P., SADA-OVALLE, I., NISHIMURA, T., ANDERSON, A. C., KUCHROO, V. K., REMOLD, H. G. & BEHAR, S. M. 2013. IL-1beta promotes antimicrobial immunity in macrophages by regulating TNFR signaling and caspase-3 activation. *J Immunol,* 190**,** 4196-204.
KAWAKAMI, K., KAWAKAMI, M., LELAND, P. & PURI, R. K. 2002. Internalization property of interleukin-4 receptor alpha chain increases cytotoxic effect of interleukin-4 receptor-targeted cytotoxin in cancer cells. *Clin Cancer Res,* 8**,** 258-66.
KILIAN, P. L., KAFFKA, K. L., BIONDI, D. A., LIPMAN, J. M., BENJAMIN, W. R., FELDMAN, D. & CAMPEN, C. A. 1991. Antiproliferative effect of interleukin-1 on human ovarian carcinoma cell line (NIH:OVCAR-3). *Cancer Res,* 51**,** 1823-8.
KIM, G. D., DAS, R., RAO, X., ZHONG, J., DEIULIIS, J. A., RAMIREZ-BERGERON, D. L., RAJAGOPALAN, S. & MAHABELESHWAR, G. H. 2018. CITED2 Restrains Proinflammatory Macrophage Activation and Response. *Mol Cell Biol,* 38.
KIM, H. S., KIM, D. C., KIM, H. M., KWON, H. J., KWON, S. J., KANG, S. J., KIM, S. C. & CHOI, G. E. 2015. STAT1 deficiency redirects IFN signalling toward suppression of TLR response through a feedback activation of STAT3. *Sci Rep,* 5**,** 13414.
KIMURA, T., NADA, S., TAKEGAHARA, N., OKUNO, T., NOJIMA, S., KANG, S., ITO, D., MORIMOTO, K., HOSOKAWA, T., HAYAMA, Y., MITSUI, Y., SAKURAI, N., SARASHINA-KIDA, H., NISHIDE, M., MAEDA, Y., TAKAMATSU, H., OKUZAKI, D., YAMADA, M., OKADA, M. & KUMANOGOH, A. 2016. Polarization of M2 macrophages requires Lamtor1 that integrates cytokine and amino-acid signals. *Nat Commun,* 7**,** 13130.
KOBAYASHI, S., SAWANO, A., NOJIMA, Y., SHIBUYA, M. & MARU, Y. 2004. The c-Cbl/CD2AP complex regulates VEGF-induced endocytosis and degradation of Flt-1 (VEGFR-1). *FASEB J,* 18**,** 929-31.
KOGA, Y., HISADA, T., ISHIZUKA, T., UTSUGI, M., ONO, A., YATOMI, M., KAMIDE, Y., AOKI-SAITO, H., TSURUMAKI, H., DOBASHI, K. & YAMADA, M. 2016. CREB regulates TNF-alpha-induced GM-CSF secretion via p38 MAPK in human lung fibroblasts. *Allergol Int,* 65**,** 406-413.
KOVALOVSKY, D., PAEZ PEREDA, M., SAUER, J., PEREZ CASTRO, C., NAHMOD, V. E., STALLA, G. K., HOLSBOER, F. & ARZT, E. 1998. The Th1 and Th2 cytokines IFN-gamma and IL-4 antagonize the inhibition of monocyte IL-1 receptor antagonist by glucocorticoids: involvement of IL-1. *Eur J Immunol,* 28**,** 2075-85.
KURGONAITE, K., GANDHI, H., KURTH, T., PAUTOT, S., SCHWILLE, P., WEIDEMANN, T. & BOKEL, C. 2015. Essential role of endocytosis for interleukin-4-receptor-mediated JAK/STAT signalling. *J Cell Sci,* 128**,** 3781-95.
LANG, R., PATEL, D., MORRIS, J. J., RUTSCHMAN, R. L. & MURRAY, P. J. 2002. Shaping gene expression in activated and resting primary macrophages by IL-10. *J Immunol,* 169**,** 2253-63.
LAWLOR, K. E., KHAN, N., MILDENHALL, A., GERLIC, M., CROKER, B. A., D'CRUZ, A. A., HALL, C., KAUR SPALL, S., ANDERTON, H., MASTERS, S. L., RASHIDI, M., WICKS, I. P., ALEXANDER, W. S., MITSUUCHI, Y., BENETATOS, C. A., CONDON, S. M., WONG, W. W., SILKE, J., VAUX, D. L. & VINCE, J. E. 2015. RIPK3 promotes cell death and NLRP3 inflammasome activation in the absence of MLKL. *Nat Commun,* 6**,** 6282.
LI, X., ZHANG, Z., LI, L., GONG, W., LAZENBY, A. J., SWANSON, B. J., HERRING, L. E., ASARA, J. M., SINGER, J. D. & WEN, H. 2017. Myeloid-derived cullin 3 promotes STAT3 phosphorylation by inhibiting OGT expression and protects against intestinal inflammation. *J Exp Med,* 214**,** 1093-1109.
LIM, J. E., CHUNG, E. & SON, Y. 2017. A neuropeptide, Substance-P, directly induces tissue-repairing M2 like macrophages by activating the PI3K/Akt/mTOR pathway even in the presence of IFNgamma. *Sci Rep,* 7**,** 9417.
LIU, J., CAO, S., HERMAN, L. M. & MA, X. 2003. Differential regulation of interleukin (IL)-12 p35 and p40 gene expression and interferon (IFN)-gamma-primed IL-12 production by IFN regulatory factor 1. *J Exp Med,* 198**,** 1265-76.

LIU, J. S., AMARAL, T. D., BROSNAN, C. F. & LEE, S. C. 1998. IFNs are critical regulators of IL-1 receptor antagonist and IL-1 expression in human microglia. *J Immunol,* 161**,** 1989-96.
LIU, X., ZHANG, J., ZEIGLER, A. C., NELSON, A. R., LINDSEY, M. L. & SAUCERMAN, J. J. 2019. Network analysis reveals a distinct axis of macrophage activation in response to conflicting inflammatory cues. *bioRxiv***,** 844464.
LO, S. Z., STEER, J. H. & JOYCE, D. A. 2011. Tumor necrosis factor-alpha promotes survival in methotrexate-exposed macrophages by an NF-kappaB-dependent pathway. *Arthritis Res Ther,* 13**,** R24.
LOPEZ-BERGAMI, P., LAU, E. & RONAI, Z. 2010. Emerging roles of ATF2 and the dynamic AP1 network in cancer. *Nat Rev Cancer,* 10**,** 65-76.
LU, P., LI, L., LIU, G., BABA, T., ISHIDA, Y., NOSAKA, M., KONDO, T., ZHANG, X. & MUKAIDA, N. 2012. Critical role of TNF-alpha-induced macrophage VEGF and iNOS production in the experimental corneal neovascularization. *Invest Ophthalmol Vis Sci,* 53**,** 3516-26.
LV, X., CHEN, P. & LIU, W. 2015. Down regulation of MiR-93 contributes to endometriosis through targeting MMP3 and VEGFA. *Am J Cancer Res,* 5**,** 1706-17.
MAITI, S., DAI, W., ALANIZ, R. C., HAHN, J. & JAYARAMAN, A. 2015. Mathematical Modeling of Pro- and Anti-Inflammatory Signaling in Macrophages. *Processes,* 3**,** 1-18.
MALIK, D. 2016. *MicroRNAs in the regulation of alternatively activated macrophages.* the University of Edinburgh.
MARCHETTI, M., MONIER, M. N., FRADAGRADA, A., MITCHELL, K., BAYCHELIER, F., EID, P., JOHANNES, L. & LAMAZE, C. 2006. Stat-mediated signaling induced by type I and type II interferons (IFNs) is differentially controlled through lipid microdomain association and clathrin-dependent endocytosis of IFN receptors. *Mol Biol Cell,* 17**,** 2896-909.
MARINO, S., HOGUE, I. B., RAY, C. J. & KIRSCHNER, D. E. 2008. A methodology for performing global uncertainty and sensitivity analysis in systems biology. *J Theor Biol,* 254**,** 178-96.
MCCOY, C. E., SHEEDY, F. J., QUALLS, J. E., DOYLE, S. L., QUINN, S. R., MURRAY, P. J. & O'NEILL, L. A. 2010. IL-10 inhibits miR-155 induction by toll-like receptors. *J Biol Chem,* 285**,** 20492-8.
MCKEOWN, S. R. 2014. Defining normoxia, physoxia and hypoxia in tumours-implications for treatment response. *Br J Radiol,* 87**,** 20130676.
MEI, Y., THOMPSON, M. D., SHIRAISHI, Y., COHEN, R. A. & TONG, X. 2014. Sarcoplasmic/endoplasmic reticulum Ca2+ ATPase C674 promotes ischemia- and hypoxia-induced angiogenesis via coordinated endothelial cell and macrophage function. *J Mol Cell Cardiol,* 76**,** 275-82.
MENG, X., GROTSCH, B., LUO, Y., KNAUP, K. X., WIESENER, M. S., CHEN, X. X., JANTSCH, J., FILLATREAU, S., SCHETT, G. & BOZEC, A. 2018. Hypoxia-inducible factor-1alpha is a critical transcription factor for IL-10-producing B cells in autoimmune disease. *Nat Commun,* 9**,** 251.
MIGITA, K., IWANAGA, N., IZUMI, Y., KAWAHARA, C., KUMAGAI, K., NAKAMURA, T., KOGA, T. & KAWAKAMI, A. 2017. TNF-alpha-induced miR-155 regulates IL-6 signaling in rheumatoid synovial fibroblasts. *BMC Res Notes,* 10**,** 403.
MITCHELL, T. J. & JOHN, S. 2005. Signal transducer and activator of transcription (STAT) signalling and T-cell lymphomas. *Immunology,* 114**,** 301-12.
MUNDER, M., MALLO, M., EICHMANN, K. & MODOLELL, M. 1998. Murine macrophages secrete interferon gamma upon combined stimulation with interleukin (IL)-12 and IL-18: A novel pathway of autocrine macrophage activation. *J Exp Med,* 187**,** 2103-8.
MUNDRA, J. J., JIAN, J., BHAGAT, P. & LIU, C. J. 2016. Progranulin inhibits expression and release of chemokines CXCL9 and CXCL10 in a TNFR1 dependent manner. *Sci Rep,* 6**,** 21115.
NAUJOKS, J., TABELING, C., DILL, B. D., HOFFMANN, C., BROWN, A. S., KUNZE, M., KEMPA, S., PETER, A., MOLLENKOPF, H. J., DORHOI, A., KERSHAW, O., GRUBER, A. D., SANDER, L. E., WITZENRATH, M., HEROLD, S., NERLICH, A., HOCKE, A. C., VAN DRIEL, I., SUTTORP, N., BEDOUI, S., HILBI, H., TROST,

M. & OPITZ, B. 2016. IFNs Modify the Proteome of Legionella-Containing Vacuoles and Restrict Infection Via IRG1-Derived Itaconic Acid. *PLoS Pathog,* 12**,** e1005408.
NEGISHI, H., OHBA, Y., YANAI, H., TAKAOKA, A., HONMA, K., YUI, K., MATSUYAMA, T., TANIGUCHI, T. & HONDA, K. 2005. Negative regulation of Toll-like-receptor signaling by IRF-4. *Proc Natl Acad Sci U S A,* 102**,** 15989-94.
NEUMANN, D., LIENENKLAUS, S., ROSATI, O. & MARTIN, M. U. 2002. IL-1beta-induced phosphorylation of PKB/Akt depends on the presence of IRAK-1. *Eur J Immunol,* 32**,** 3689-98.
NIEMAND, C., NIMMESGERN, A., HAAN, S., FISCHER, P., SCHAPER, F., ROSSAINT, R., HEINRICH, P. C. & MULLER-NEWEN, G. 2003. Activation of STAT3 by IL-6 and IL-10 in primary human macrophages is differentially modulated by suppressor of cytokine signaling 3. *J Immunol,* 170**,** 3263-72.
NOWAK, D. G., WOOLARD, J., AMIN, E. M., KONOPATSKAYA, O., SALEEM, M. A., CHURCHILL, A. J., LADOMERY, M. R., HARPER, S. J. & BATES, D. O. 2008. Expression of pro- and anti-angiogenic isoforms of VEGF is differentially regulated by splicing and growth factors. *J Cell Sci,* 121**,** 3487-95.
O'CONNELL, R. M., TAGANOV, K. D., BOLDIN, M. P., CHENG, G. & BALTIMORE, D. 2007. MicroRNA-155 is induced during the macrophage inflammatory response. *Proc Natl Acad Sci U S A,* 104**,** 1604-9.
O'CONNOR, J. C., SHERRY, C. L., GUEST, C. B. & FREUND, G. G. 2007. Type 2 diabetes impairs insulin receptor substrate-2-mediated phosphatidylinositol 3-kinase activity in primary macrophages to induce a state of cytokine resistance to IL-4 in association with overexpression of suppressor of cytokine signaling-3. *J Immunol,* 178**,** 6886-93.
O'SHEA, J. J., SCHWARTZ, D. M., VILLARINO, A. V., GADINA, M., MCINNES, I. B. & LAURENCE, A. 2015. The JAK-STAT pathway: impact on human disease and therapeutic intervention. *Annu Rev Med,* 66**,** 311-28.
OHATA, T., FUKUDA, K., MURAKAMI, A., OHIGASHI, H., SUGIMURA, T. & WAKABAYASHI, K. 1998. Inhibition by 1'-acetoxychavicol acetate of lipopolysaccharide- and interferon-gamma-induced nitric oxide production through suppression of inducible nitric oxide synthase gene expression in RAW264 cells. *Carcinogenesis,* 19**,** 1007-12.
ONO, H., ICHIKI, T., OHTSUBO, H., FUKUYAMA, K., IMAYAMA, I., IINO, N., MASUDA, S., HASHIGUCHI, Y., TAKESHITA, A. & SUNAGAWA, K. 2006. CAMP-response element-binding protein mediates tumor necrosis factor-alpha-induced vascular cell adhesion molecule-1 expression in endothelial cells. *Hypertens Res,* 29**,** 39-47.
PEIHENG HE, F. Y., SHUAI HUANG, YUANQING GUO, HUA WANG, YUTIAN WU 2016. Anti-inflammatory effect of pristimerin on TNFα-induced inflammatory responses in murine macrophages. *Int J Clin Exp Pathol,* 9**,** 1186-1194.
PICCOLO, V., CURINA, A., GENUA, M., GHISLETTI, S., SIMONATTO, M., SABO, A., AMATI, B., OSTUNI, R. & NATOLI, G. 2017. Opposing macrophage polarization programs show extensive epigenomic and transcriptional cross-talk. *Nat Immunol,* 18**,** 530-540.
PLOTNIKOV, A., ZEHORAI, E., PROCACCIA, S. & SEGER, R. 2011. The MAPK cascades: signaling components, nuclear roles and mechanisms of nuclear translocation. *Biochim Biophys Acta,* 1813**,** 1619-33.
RAHIM, S. S., KHAN, N., BODDUPALLI, C. S., HASNAIN, S. E. & MUKHOPADHYAY, S. 2005. Interleukin-10 (IL-10) mediated suppression of IL-12 production in RAW 264.7 cells also involves c-rel transcription factor. *Immunology,* 114**,** 313-21.
RAMANATHAN, M., PINHAL-ENFIELD, G., HAO, I. & LEIBOVICH, S. J. 2007. Synergistic up-regulation of vascular endothelial growth factor (VEGF) expression in macrophages by adenosine A2A receptor agonists and endotoxin involves transcriptional regulation via the hypoxia response element in the VEGF promoter. *Mol Biol Cell,* 18**,** 14-23.

REN, J., CHEN, X. & CHEN, Z. J. 2014. IKKbeta is an IRF5 kinase that instigates inflammation. *Proc Natl Acad Sci U S A,* 111**,** 17438-43.
REX, J., ALBRECHT, U., EHLTING, C., THOMAS, M., ZANGER, U. M., SAWODNY, O., HAUSSINGER, D., EDERER, M., FEUER, R. & BODE, J. G. 2016. Model-Based Characterization of Inflammatory Gene Expression Patterns of Activated Macrophages. *PLoS Comput Biol,* 12**,** e1005018.
SATO, A., OHTAKI, H., TSUMURAYA, T., SONG, D., OHARA, K., ASANO, M., IWAKURA, Y., ATSUMI, T. & SHIODA, S. 2012. Interleukin-1 participates in the classical and alternative activation of microglia/macrophages after spinal cord injury. *J Neuroinflammation,* 9**,** 65.
SCHINDLER, H., LUTZ, M. B., ROLLINGHOFF, M. & BOGDAN, C. 2001. The production of IFN-gamma by IL-12/IL-18-activated macrophages requires STAT4 signaling and is inhibited by IL-4. *J Immunol,* 166**,** 3075-82.
SCHLEICHER, U., PADUCH, K., DEBUS, A., OBERMEYER, S., KONIG, T., KLING, J. C., RIBECHINI, E., DUDZIAK, D., MOUGIAKAKOS, D., MURRAY, P. J., OSTUNI, R., KORNER, H. & BOGDAN, C. 2016. TNF-Mediated Restriction of Arginase 1 Expression in Myeloid Cells Triggers Type 2 NO Synthase Activity at the Site of Infection. *Cell Rep,* 15**,** 1062-1075.
SHELDON, K. E., SHANDILYA, H., KEPKA-LENHART, D., POLJAKOVIC, M., GHOSH, A. & MORRIS, S. M., JR. 2013. Shaping the murine macrophage phenotype: IL-4 and cyclic AMP synergistically activate the arginase I promoter. *J Immunol,* 191**,** 2290-8.
SHEMBADE, N., HARHAJ, N. S., LIEBL, D. J. & HARHAJ, E. W. 2007. Essential role for TAX1BP1 in the termination of TNF-alpha-, IL-1- and LPS-mediated NF-kappaB and JNK signaling. *EMBO J,* 26**,** 3910-22.
SHEMBADE, N., HARHAJ, N. S., PARVATIYAR, K., COPELAND, N. G., JENKINS, N. A., MATESIC, L. E. & HARHAJ, E. W. 2008. The E3 ligase Itch negatively regulates inflammatory signaling pathways by controlling the function of the ubiquitin-editing enzyme A20. *Nat Immunol,* 9**,** 254-62.
SHEMBADE, N., MA, A. & HARHAJ, E. W. 2010. Inhibition of NF-kappaB signaling by A20 through disruption of ubiquitin enzyme complexes. *Science,* 327**,** 1135-9.
SHEMBADE, N., PARVATIYAR, K., HARHAJ, N. S. & HARHAJ, E. W. 2009. The ubiquitin-editing enzyme A20 requires RNF11 to downregulate NF-kappaB signalling. *EMBO J,* 28**,** 513-22.
STAPLES, K. J., SMALLIE, T., WILLIAMS, L. M., FOEY, A., BURKE, B., FOXWELL, B. M. & ZIEGLER-HEITBROCK, L. 2007. IL-10 induces IL-10 in primary human monocyte-derived macrophages via the transcription factor Stat3. *J Immunol,* 178**,** 4779-85.
SUPRUNENKO, T. & HOFER, M. J. 2016. The emerging role of interferon regulatory factor 9 in the antiviral host response and beyond. *Cytokine Growth Factor Rev,* 29**,** 35-43.
SUZUKI, N., SUZUKI, S., DUNCAN, G. S., MILLAR, D. G., WADA, T., MIRTSOS, C., TAKADA, H., WAKEHAM, A., ITIE, A., LI, S., PENNINGER, J. M., WESCHE, H., OHASHI, P. S., MAK, T. W. & YEH, W. C. 2002. Severe impairment of interleukin-1 and Toll-like receptor signalling in mice lacking IRAK-4. *Nature,* 416**,** 750-6.
TACHDJIAN, R., AL KHATIB, S., SCHWINGLSHACKL, A., KIM, H. S., CHEN, A., BLASIOLI, J., MATHIAS, C., KIM, H. Y., UMETSU, D. T., OETTGEN, H. C. & CHATILA, T. A. 2010. In vivo regulation of the allergic response by the IL-4 receptor alpha chain immunoreceptor tyrosine-based inhibitory motif. *J Allergy Clin Immunol,* 125**,** 1128-1136 e8.
TAKEDA, N., O'DEA, E. L., DOEDENS, A., KIM, J. W., WEIDEMANN, A., STOCKMANN, C., ASAGIRI, M., SIMON, M. C., HOFFMANN, A. & JOHNSON, R. S. 2010. Differential activation and antagonistic function of HIF-{alpha} isoforms in macrophages are essential for NO homeostasis. *Genes Dev,* 24**,** 491-501.
TIAN, F., YUAN, C., HU, L. & SHAN, S. 2017. MicroRNA-93 inhibits inflammatory responses and cell apoptosis after cerebral ischemia reperfusion by targeting interleukin-1 receptor-associated kinase 4. *Exp Ther Med,* 14**,** 2903-2910.

TODA, Y., TSUKADA, J., MISAGO, M., KOMINATO, Y., AURON, P. E. & TANAKA, Y. 2002. Autocrine induction of the human pro-IL-1beta gene promoter by IL-1beta in monocytes. *J Immunol,* 168**,** 1984-91.

TRAN, T. M., TEMKIN, V., SHI, B., PAGLIARI, L., DANIEL, S., FERRAN, C. & POPE, R. M. 2009. TNFalpha-induced macrophage death via caspase-dependent and independent pathways. *Apoptosis,* 14**,** 320-32.

TRASK, O. J., JR. 2004. Nuclear Factor Kappa B (NF-kappaB) Translocation Assay Development and Validation for High Content Screening. *In:* SITTAMPALAM, G. S., GROSSMAN, A., BRIMACOMBE, K., ARKIN, M., AULD, D., AUSTIN, C. P., BAELL, J., BEJCEK, B., CAAVEIRO, J. M. M., CHUNG, T. D. Y., COUSSENS, N. P., DAHLIN, J. L., DEVANARYAN, V., FOLEY, T. L., GLICKSMAN, M., HALL, M. D., HAAS, J. V., HOARE, S. R. J., INGLESE, J., IVERSEN, P. W., KAHL, S. D., KALES, S. C., KIRSHNER, S., LAL-NAG, M., LI, Z., MCGEE, J., MCMANUS, O., RISS, T., SARADJIAN, P., TRASK, O. J., JR., WEIDNER, J. R., WILDEY, M. J., XIA, M. & XU, X. (eds.) *Assay Guidance Manual.* Bethesda (MD).

TUCKERMAN, J. R., ZHAO, Y., HEWITSON, K. S., TIAN, Y. M., PUGH, C. W., RATCLIFFE, P. J. & MOLE, D. R. 2004. Determination and comparison of specific activity of the HIF-prolyl hydroxylases. *FEBS Lett,* 576**,** 145-50.

UEKI, K., KONDO, T. & KAHN, C. R. 2004. Suppressor of cytokine signaling 1 (SOCS-1) and SOCS-3 cause insulin resistance through inhibition of tyrosine phosphorylation of insulin receptor substrate proteins by discrete mechanisms. *Mol Cell Biol,* 24**,** 5434-46.

VAREY, A. H., RENNEL, E. S., QIU, Y., BEVAN, H. S., PERRIN, R. M., RAFFY, S., DIXON, A. R., PARASKEVA, C., ZACCHEO, O., HASSAN, A. B., HARPER, S. J. & BATES, D. O. 2008. VEGF 165 b, an antiangiogenic VEGF-A isoform, binds and inhibits bevacizumab treatment in experimental colorectal carcinoma: balance of pro- and antiangiogenic VEGF-A isoforms has implications for therapy. *Br J Cancer,* 98**,** 1366-79.

VEREMEYKO, T., YUNG, A. W. Y., ANTHONY, D. C., STREKALOVA, T. & PONOMAREV, E. D. 2018. Early Growth Response Gene-2 Is Essential for M1 and M2 Macrophage Activation and Plasticity by Modulation of the Transcription Factor CEBPbeta. *Front Immunol,* 9**,** 2515.

VILA-DEL SOL, V., DIAZ-MUNOZ, M. D. & FRESNO, M. 2007. Requirement of tumor necrosis factor alpha and nuclear factor-kappaB in the induction by IFN-gamma of inducible nitric oxide synthase in macrophages. *J Leukoc Biol,* 81**,** 272-83.

VILA-DEL SOL, V., PUNZON, C. & FRESNO, M. 2008. IFN-gamma-induced TNF-alpha expression is regulated by interferon regulatory factors 1 and 8 in mouse macrophages. *J Immunol,* 181**,** 4461-70.

VILLALTA, S. A., RINALDI, C., DENG, B., LIU, G., FEDOR, B. & TIDBALL, J. G. 2011. Interleukin-10 reduces the pathology of mdx muscular dystrophy by deactivating M1 macrophages and modulating macrophage phenotype. *Hum Mol Genet,* 20**,** 790-805.

VOLLMER, S., STRICKSON, S., ZHANG, T., GRAY, N., LEE, K. L., RAO, V. R. & COHEN, P. 2017. The mechanism of activation of IRAK1 and IRAK4 by interleukin-1 and Toll-like receptor agonists. *Biochem J,* 474**,** 2027-2038.

WANG, Z., JI, J., PENG, D., MA, F., CHENG, G. & QIN, F. X. 2016. Complex Regulation Pattern of IRF3 Activation Revealed by a Novel Dimerization Reporter System. *J Immunol,* 196**,** 4322-30.

WATERFIELD, M., JIN, W., REILEY, W., ZHANG, M. & SUN, S. C. 2004. IkappaB kinase is an essential component of the Tpl2 signaling pathway. *Mol Cell Biol,* 24**,** 6040-8.

WEI, S. H., MING-LUM, A., LIU, Y., WALLACH, D., ONG, C. J., CHUNG, S. W., MOORE, K. W. & MUI, A. L. 2006. Proteasome-mediated proteolysis of the interleukin-10 receptor is important for signal downregulation. *J Interferon Cytokine Res,* 26**,** 281-90.

WHEELER, K. C., JENA, M. K., PRADHAN, B. S., NAYAK, N., DAS, S., HSU, C. D., WHEELER, D. S., CHEN, K. & NAYAK, N. R. 2018. VEGF may contribute to macrophage recruitment and M2 polarization in the decidua. *PLoS One,* 13**,** e0191040.

WINSTON, B. W., CHAN, E. D., JOHNSON, G. L. & RICHES, D. W. 1997. Activation of p38mapk, MKK3, and MKK4 by TNF-alpha in mouse bone marrow-derived macrophages. *J Immunol,* 159**,** 4491-7.

WORMALD, S., ZHANG, J. G., KREBS, D. L., MIELKE, L. A., SILVER, J., ALEXANDER, W. S., SPEED, T. P., NICOLA, N. A. & HILTON, D. J. 2006. The comparative roles of suppressor of cytokine signaling-1 and -3 in the inhibition and desensitization of cytokine signaling. *J Biol Chem,* 281**,** 11135-43.

WU, C., XUE, Y., WANG, P., LIN, L., LIU, Q., LI, N., XU, J. & CAO, X. 2014. IFN-gamma primes macrophage activation by increasing phosphatase and tensin homolog via downregulation of miR-3473b. *J Immunol,* 193**,** 3036-44.

XUE, H., TU, Y., MA, T., WEN, T., YANG, T., XUE, L., CAI, M., WANG, F. & GUAN, M. 2019. miR-93-5p attenuates IL-1beta-induced chondrocyte apoptosis and cartilage degradation in osteoarthritis partially by targeting TCF4. *Bone,* 123**,** 129-136.

YAO, Y., WANG, Y., ZHANG, Z., HE, L., ZHU, J., ZHANG, M., HE, X., CHENG, Z., AO, Q., CAO, Y., YANG, P., SU, Y., ZHAO, J., ZHANG, S., YU, Q., NING, Q., XIANG, X., XIONG, W., WANG, C. Y. & XU, Y. 2016. Chop Deficiency Protects Mice Against Bleomycin-induced Pulmonary Fibrosis by Attenuating M2 Macrophage Production. *Mol Ther,* 24**,** 915-25.

YASUKAWA, H., OHISHI, M., MORI, H., MURAKAMI, M., CHINEN, T., AKI, D., HANADA, T., TAKEDA, K., AKIRA, S., HOSHIJIMA, M., HIRANO, T., CHIEN, K. R. & YOSHIMURA, A. 2003. IL-6 induces an anti-inflammatory response in the absence of SOCS3 in macrophages. *Nat Immunol,* 4**,** 551-6.

YEE, D., SHAH, K. M., COLES, M. C., SHARP, T. V. & LAGOS, D. 2017. MicroRNA-155 induction via TNF-alpha and IFN-gamma suppresses expression of programmed death ligand-1 (PD-L1) in human primary cells. *J Biol Chem,* 292**,** 20683-20693.

YIN, M., YANG, S. Q., LIN, H. Z., LANE, M. D., CHATTERJEE, S. & DIEHL, A. M. 1996. Tumor necrosis factor alpha promotes nuclear localization of cytokine-inducible CCAAT/enhancer binding protein isoforms in hepatocytes. *J Biol Chem,* 271**,** 17974-8.

ZANIN, R. F., BRAGANHOL, E., BERGAMIN, L. S., CAMPESATO, L. F., FILHO, A. Z., MOREIRA, J. C., MORRONE, F. B., SEVIGNY, J., SCHETINGER, M. R., DE SOUZA WYSE, A. T. & BATTASTINI, A. M. 2012. Differential macrophage activation alters the expression profile of NTPDase and ecto-5'-nucleotidase. *PLoS One,* 7**,** e31205.

ZETTERBERG, A. & SKOLD, O. 1969. The effect of serum starvation on DNA, RNA and protein synthesis during interphase in L-cells. *Exp Cell Res,* 57**,** 114-8.

ZHANG, H. C., WHITE, K. B., YE, H., MCCOMSEY, D. F., DERIAN, C. K., ADDO, M. F., ANDRADE-GORDON, P., ECKARDT, A. J., CONWAY, B. R., WESTOVER, L., XU, J. Z., LOOK, R., DEMAREST, K. T., EMANUEL, S. & MARYANOFF, B. E. 2003. Macrocyclic bisindolylmaleimides as inhibitors of protein kinase C and glycogen synthase kinase-3. *Bioorg Med Chem Lett,* 13**,** 3049-53.

ZHANG, W., XU, W. & XIONG, S. 2011. Macrophage differentiation and polarization via phosphatidylinositol 3-kinase/Akt-ERK signaling pathway conferred by serum amyloid P component. *J Immunol,* 187**,** 1764-77.

ZHAO, C., MIRANDO, A. C., SOVE, R. J., MEDEIROS, T. X., ANNEX, B. H. & POPEL, A. S. 2019. A mechanistic integrative computational model of macrophage polarization: Implications in human pathophysiology. *PLoS Comput Biol,* 15**,** e1007468.
